# Supplementary material for: Social determinants of pertussis and influenza vaccine uptake in pregnancy: a national cohort study in England using electronic health records
Source: BMJ Open. 2021 Jun 21;11(6):e046545. doi: 10.1136/bmjopen-2020-046545 (PMC8217954; doi:10.1136/bmjopen-2020-046545)
Supplement: Supplementary data [file bmjopen-2020-046545supp001.pdf]

Supplementary material

Social determinants of pertussis and influenza vaccine uptake in pregnancy: a national cohort study using electronic health records

Authors: Jemma L Walker,* Christopher T Rentsch,* Helen I McDonald, Jeongeun Bak, Caroline Minassian, Gayatri Amirthalingam, Michael Edelstein, Sara L Thomas.

Supplementary Table 1: Hierarchical conceptual framework and interpretation of effect estimates

Supplementary Table 2: Patterns of social factors amongst pregnant women with and without a recorded ethnicity status, 2010-2015

Supplementary Table 3: 'Pertussis BMI Model' complete case analysis additionally excluding 6,666 women with missing BMI for pertussis vaccine uptake amongst pregnant women in the UK, 2012-2015

Supplementary Table 4: 'Influenza BMI Model' complete case analysis additionally excluding 13,874 women with missing BMI for influenza vaccine uptake amongst pregnant women in the UK, 2010-2015

Supplementary Table 5: Sensitivity analyses expanding definition of inclusion criteria for the pertussis vaccine uptake models: registration by end of pregnancy and ImmForm approach compared to primary analyses

Supplementary Table 7: Secondary analysis of subsequent pertussis vaccine uptake among women who had received pertussis vaccination in their first eligible pregnancy and had a second eligible pregnancy within the study period (N=3,111)

Walker JL, *et al. BMJ Open* 2021; 11:e046545. doi: 10.1136/bmjopen-2020-046545

Supplementary Table 1: Hierarchical conceptual framework and interpretation of effect estimates

This table is reproduced from Supplementary Table 6 in Jain A., Walker JL, Forbes H, Langan S, Smeeth L, van Hoek AJ and Thomas SL. Zoster vaccination inequalities: A population based cohort study using linked data from the UK Clinical Practice Research Datalink. PLoS One 2018;13(11):e0207183. doi: 10.1371/journal.pone.0207183.

(based on [1])

Hierarchical models	Explanatory variables	Interpretation of effect estimates
\`Minimally' adjusted model	Each explanatory variable adjusted in-turn for *a priori* confounders: year of birth and gender	Effect estimate of each variable adjusted for *a priori* confounders.
Model-1*^	Ethnicity +immigration status^ with *a priori* confounders	Effects of ethnicity and immigration status adjusted for each other and *a priori* confounders
Model-2*	Model-1+ patient-LSOA-level deprivation#	(i) Effects of ethnicity and immigration status not mediated via deprivation and adjusted for each other and *a priori* confounders<br>(ii) Effect of patient-LSOA-level deprivation adjusted for *a priori* confounders, ethnicity and immigration status
Model-3*	Model-2 + rest of the explanatory variables~	(i) Effect of ethnicity and immigration status not mediated via deprivation and other explanatory variables~ *<br>(ii) Effect of deprivation not mediated via other explanatory variables~*<br>(iii) Effect of other explanatory variables~ *

*all variables in the model adjusted for each other and *a priori* confounders: year of birth, sex and calendar period ^ethnicity and immigration status examined for multicollinearity LSOA Lower-layer Super Output Area # patient-LSOA-level and practice-LSOA-level deprivation were considered to be correlated therefore only patient-LSOA-level deprivation used ~ care home residence, living alone status and cohabitation status (living alone and cohabitation examined for multicollinearity)

1. Victora CG, Huttly SR, Fuchs SC, Olinto MT. The role of conceptual frameworks in epidemiological analysis: a hierarchical approach. Int J Epidemiol. 1997;26(1):224-7. PubMed PMID: 9126524.

Supplementary Table 2: Patterns of social factors amongst pregnant women with and without a recorded ethnicity status, 2010-2015

		Pertussis		Influenza	
		Recorded ethnicity	Missing ethnicity	Recorded ethnicity	Missing ethnicity
		n=62,537	n=5,553	n=140,141	n=11,991
Year/season	2010	-	-	34,373 (24.5%)	2,433 (20.3%)
	2011	-	-	32,258 (23.0%)	2,228 (18.6%)
	2012	6,717 (10.7%)	506 (9.1%)	26,750 (19.1%)	1,791 (14.9%)
	2013	24,657 (39.4%)	1,789 (32.2%)	21,029 (15.0%)	1,730 (14.4%)
	2014	20,148 (32.2%)	1,910 (34.4%)	15,712 (11.2%)	1,882 (15.7%)
	2015	11,015 (17.6%)	1,348 (24.3%)	10,019 (7.1%)	1,927 (16.1%)
Index of multiple deprivation (IMD) quintile	Least deprived	13,285 (21.2%)	1,522 (27.4%)	28,956 (20.7%)	3,203 (26.7%)
	2	11,335 (18.1%)	883 (15.9%)	25,424 (18.1%)	1,896 (15.8%)
	3	12,933 (20.7%)	992 (17.9%)	29,368 (21.0%)	2,245 (18.7%)
	4	12,973 (20.7%)	1,592 (28.7%)	28,520 (20.4%)	3,265 (27.2%)
	Most deprived	12,011 (19.2%)	564 (10.2%)	27,873 (19.9%)	1,382 (11.5%)
Region	London	11,894 (19.0%)	502 (9.0%)	26,171 (18.7%)	1,144 (9.5%)
	North East	1,185 (1.9%)	60 (1.1%)	2,758 (2.0%)	173 (1.4%)
	North West	8,835 (14.1%)	917 (16.5%)	19,060 (13.6%)	1,761 (14.7%)
	Yorkshire & The Humber	1,000 (1.6%)	5 (0.1%)	2,840 (2.0%)	24 (0.2%)
	East Midlands	326 (0.5%)	70 (1.3%)	1,940 (1.4%)	435 (3.6%)
	West Midlands	7,050 (11.3%)	530 (9.5%)	15,846 (11.3%)	1,231 (10.3%)
	East of England	5,568 (8.9%)	464 (8.4%)	13,695 (9.8%)	1,025 (8.5%)
	South West	7,002 (11.2%)	223 (4.0%)	16,546 (11.8%)	574 (4.8%)
	South Central	10,381 (16.6%)	1,692 (30.5%)	21,435 (15.3%)	3,215 (26.8%)
	South East Coast	9,296 (14.9%)	1,090 (19.6%)	19,850 (14.2%)	2,409 (20.1%)
Ethnicity	White	52,598 (84.1%)	-	117,469 (83.8%)	-
	South Asian	4,692 (7.5%)	-	10,827 (7.7%)	-
	Black	2,583 (4.1%)	-	5,853 (4.2%)	-
	Mixed	922 (1.5%)	-	2,094 (1.5%)	-
	Other	1,742 (2.8%)	-	3,898 (2.8%)	-
Maternal age, years	<20	2,079 (3.3%)	218 (3.9%)	5,536 (4.0%)	583 (4.9%)
	20-24	8,848 (14.1%)	914 (16.5%)	21,663 (15.5%)	2,014 (16.8%)
	25-29	16,696 (26.7%)	1,391 (25.0%)	37,985 (27.1%)	3,004 (25.1%)
	30-35	20,294 (32.5%)	1,673 (30.1%)	43,777 (31.2%)	3,639 (30.3%)
	≥35	14,620 (23.4%)	1,357 (24.4%)	31,180 (22.2%)	2,751 (22.9%)
Number of children	0	26,622 (42.6%)	2,645 (47.6%)	66,112 (47.2%)	6,255 (52.2%)
	1	22,132 (35.4%)	1,675 (30.2%)	45,969 (32.8%)	3,312 (27.6%)
	2	8,645 (13.8%)	679 (12.2%)	18,192 (13.0%)	1,431 (11.9%)
	≥3	5,138 (8.2%)	554 (10.0%)	9,868 (7.0%)	993 (8.3%)
Clinical risk group	No	-	-	130,160 (92.9%)	11,238 (93.7%)
	Yes	-	-	9,981 (7.1%)	753 (6.3%)
Body mass index (BMI)	<18.5	2,063 (3.3%)	201 (3.6%)	4,865 (3.5%)	434 (3.6%)
	18.5-24.9	29,045 (46.4%)	2,489 (44.8%)	66,405 (47.4%)	5,571 (46.5%)
	25.0-29.9	14,211 (22.7%)	1,203 (21.7%)	31,855 (22.7%)	2,563 (21.4%)
	≥30	10,552 (16.9%)	785 (14.1%)	23,142 (16.5%)	1,747 (14.6%)
	Missing	6,666 (10.7%)	875 (15.8%)	13,874 (9.9%)	1,676 (14.0%)
Note: all p<0.001					

Walker JL, *et al. BMJ Open* 2021; 11:e046545. doi: 10.1136/bmjopen-2020-046545

Supplementary Table 3: 'Pertussis BMI Model' complete case analysis additionally excluding 6,666 women with missing BMI for pertussis vaccine uptake amongst pregnant women in the UK, 2012-2015

		**Minimally adjusted** for year	**Model 3 (fully adjusted in main analysis)** Adjusted for year, IMD, region, ethnicity, maternal age and number of children	**BMI Model** As Model 3 and additionally adjusted for BMI
N		55,871	55,871	55,871
Year	2012	1	1	1
	2013	1.65 (1.56, 1.75)	1.74 (1.63, 1.84)	1.74 (1.63, 1.84)
	2014	1.63 (1.54, 1.73)	1.70 (1.60, 1.81)	1.70 (1.60, 1.81)
	2015	1.95 (1.82, 2.08)	2.04 (1.90, 2.19)	2.04 (1.91, 2.19)
Index of multiple deprivation (IMD) quintile	Least deprived	1	1	1
	2	0.78 (0.73, 0.83)	0.81 (0.76, 0.86)	0.81 (0.76, 0.86)
	3	0.67 (0.64, 0.71)	0.72 (0.68, 0.77)	0.72 (0.68, 0.77)
	4	0.54 (0.51, 0.58)	0.63 (0.59, 0.67)	0.63 (0.59, 0.67)
	Most deprived	0.44 (0.41, 0.46)	0.53 (0.50, 0.57)	0.54 (0.50, 0.57)
Region	London	1	1	1
	North East	0.96 (0.84, 1.10)	1.12 (0.98, 1.29)	1.12 (0.97, 1.28)
	North West	1.30 (1.22, 1.38)	1.36 (1.28, 1.46)	1.36 (1.28, 1.46)
	Yorkshire & The Humber	1.49 (1.29, 1.72)	1.51 (1.30, 1.76)	1.51 (1.30, 1.76)
	East Midlands	1.87 (1.44, 2.42)	2.33 (1.78, 3.04)	2.31 (1.77, 3.02)
	West Midlands	1.60 (1.50, 1.71)	1.70 (1.59, 1.83)	1.70 (1.58, 1.82)
	East of England	1.73 (1.60, 1.86)	1.56 (1.44, 1.68)	1.56 (1.44, 1.68)
	South West	1.41 (1.32, 1.51)	1.42 (1.33, 1.53)	1.42 (1.33, 1.53)
	South Central	1.46 (1.37, 1.55)	1.29 (1.21, 1.37)	1.29 (1.21, 1.37)
	South East Coast	1.33 (1.26, 1.42)	1.18 (1.11, 1.26)	1.18 (1.11, 1.26)
Ethnicity	White	1	1	1
	South Asian	0.74 (0.70, 0.79)	0.83 (0.77, 0.89)	0.83 (0.77, 0.89)
	Black	0.45 (0.41, 0.49)	0.62 (0.57, 0.68)	0.62 (0.56, 0.67)
	Mixed	0.69 (0.60, 0.79)	0.75 (0.65, 0.87)	0.75 (0.65, 0.87)
	Other	0.63 (0.57, 0.70)	0.67 (0.60, 0.75)	0.68 (0.61, 0.75)
Maternal age, years	<20	0.85 (0.75, 0.96)	0.84 (0.74, 0.96)	0.85 (0.75, 0.97)
	20-24	1	1	1
	25-29	1.27 (1.20, 1.34)	1.28 (1.20, 1.36)	1.27 (1.20, 1.35)
	30-35	1.49 (1.41, 1.58)	1.51 (1.42, 1.60)	1.49 (1.41, 1.58)
	≥35	1.32 (1.24, 1.40)	1.37 (1.29, 1.46)	1.36 (1.28, 1.45)
Number of children	0	1	1	1
	1	0.67 (0.65, 0.70)	0.66 (0.63, 0.68)	0.66 (0.63, 0.68)
	2	0.47 (0.44, 0.49)	0.47 (0.45, 0.50)	0.47 (0.45, 0.50)
	≥3	0.35 (0.33, 0.38)	0.37 (0.35, 0.40)	0.37 (0.35, 0.40)
Body mass index (BMI)	<18.5	0.71 (0.64, 0.77)		0.77 (0.70, 0.85)
	18.5-24.9	1		1
	25.0-29.9	1.01 (0.96, 1.05)		1.10 (1.05, 1.15)
	≥30	0.82 (0.78, 0.86)		0.96 (0.91, 1.00)
Note: Model inclusion as per the main analysis but additionally excluding 6,666 women with missing BMI				

Supplementary Table 4: 'Influenza BMI Model' complete case analysis additionally excluding 13,874 women with missing BMI for influenza vaccine uptake amongst pregnant women in the UK, 2010-2015

		**Minimally adjusted** for year	**Model 4** adjusted for year, IMD,region, ethnicity, maternal age, number of children and clinical risk group	**BMI Model** as Model 4 and additionally adjusted for BMI
N		126,267	126,267	126,267
Year	2010	1	1	1
	2011	0.90 (0.87, 0.93)	0.90 (0.87, 0.93)	0.90 (0.87, 0.93)
	2012	1.63 (1.57, 1.68)	1.64 (1.59, 1.70)	1.64 (1.59, 1.70)
	2013	1.41 (1.36, 1.46)	1.41 (1.35, 1.46)	1.40 (1.35, 1.46)
	2014	1.70 (1.63, 1.77)	1.70 (1.63, 1.77)	1.70 (1.63, 1.77)
	2015	1.66 (1.58, 1.74)	1.67 (1.60, 1.76)	1.67 (1.59, 1.76)
Index of multiple deprivation (IMD) quintile	Least deprived	1	1	1
	2	0.90 (0.87, 0.94)	0.92 (0.89, 0.95)	0.92 (0.88, 0.95)
	3	0.83 (0.80, 0.86)	0.87 (0.84, 0.90)	0.86 (0.83, 0.90)
	4	0.71 (0.69, 0.74)	0.76 (0.73, 0.79)	0.75 (0.73, 0.78)
	Most deprived	0.68 (0.65, 0.70)	0.72 (0.69, 0.75)	0.71 (0.69, 0.74)
Region	London	1	1	1
	North East	1.17 (1.07, 1.28)	1.26 (1.15, 1.37)	1.25 (1.14, 1.37)
	North West	1.40 (1.34, 1.45)	1.43 (1.37, 1.49)	1.43 (1.37, 1.49)
	Yorkshire & The Humber	1.28 (1.18, 1.39)	1.26 (1.16, 1.37)	1.25 (1.15, 1.36)
	East Midlands	1.29 (1.17, 1.43)	1.35 (1.22, 1.49)	1.34 (1.21, 1.49)
	West Midlands	1.41 (1.35, 1.47)	1.44 (1.37, 1.50)	1.43 (1.37, 1.49)
	East of England	1.30 (1.24, 1.36)	1.23 (1.17, 1.28)	1.22 (1.17, 1.28)
	South West	1.29 (1.23, 1.34)	1.28 (1.22, 1.34)	1.27 (1.22, 1.33)
	South Central	1.45 (1.39, 1.51)	1.35 (1.30, 1.41)	1.35 (1.29, 1.40)
	South East Coast	1.08 (1.04, 1.12)	1.03 (0.99, 1.07)	1.03 (0.99, 1.07)
Ethnicity	White	1	1	1
	South Asian	0.90 (0.87, 0.94)	0.99 (0.95, 1.03)	0.99 (0.95, 1.04)
	Black	0.66 (0.62, 0.70)	0.83 (0.78, 0.88)	0.82 (0.77, 0.87)
	Mixed	0.85 (0.78, 0.94)	0.92 (0.84, 1.01)	0.92 (0.84, 1.01)
	Other	0.78 (0.73, 0.84)	0.86 (0.80, 0.92)	0.87 (0.80, 0.93)
Maternal age, years	<20	0.90 (0.84, 0.98)	0.90 (0.83, 0.97)	0.91 (0.84, 0.98)
	20-24	1	1	1
	25-29	1.12 (1.08, 1.16)	1.11 (1.07, 1.15)	1.11 (1.07, 1.15)
	30-35	1.20 (1.16, 1.24)	1.19 (1.15, 1.23)	1.19 (1.14, 1.23)
	≥35	1.14 (1.10, 1.18)	1.15 (1.11, 1.20)	1.15 (1.10, 1.19)
Number of children	0	1	1	1
	1	0.80 (0.78, 0.82)	0.79 (0.77, 0.81)	0.79 (0.77, 0.81)
	2	0.70 (0.68, 0.73)	0.71 (0.68, 0.73)	0.70 (0.68, 0.73)
	≥3	0.61 (0.58, 0.64)	0.62 (0.59, 0.66)	0.62 (0.59, 0.65)
Clinical risk group	No	1	1	1
	Yes	1.69 (1.62, 1.76)	1.69 (1.62, 1.77)	1.68 (1.61, 1.76)
Body mass index (BMI)	<18.5	0.85 (0.80, 0.90)		0.89 (0.84, 0.95)
	18.5-24.9	1		1
	25.0-29.9	1.04 (1.01, 1.07)		1.07 (1.04, 1.10)
	≥30	1.00 (0.97, 1.03)		1.06 (1.03, 1.09)
Note: Model inclusion as per the main analysis but additionally excluding 13,874 women with missing BMI				

Supplementary Table 5: Sensitivity analyses expanding definition of inclusion criteria for the pertussis vaccine uptake models: registration by end of pregnancy and ImmForm approach compared to primary analyses

		Primary analyses		Registered by end of pregnancy		ImmForm approach	
		**Minimally adjusted**	**Fully adjusted**	**Minimally adjusted**	**Fully adjusted**	**Minimally adjusted**	**Fully adjusted**
N		62,537	62,537	80,831	80,831	90,720	90,720
Year	2012	1	1	1	1	1	1
	2013	1.62 (1.53, 1.71)	1.69 (1.60, 1.79)	1.59 (1.52, 1.67)	1.65 (1.58, 1.73)	1.55 (1.48, 1.62)	1.60 (1.53, 1.67)
	2014	1.60 (1.51, 1.69)	1.66 (1.57, 1.76)	1.69 (1.61, 1.77)	1.72 (1.64, 1.81)	1.64 (1.57, 1.72)	1.67 (1.60, 1.75)
	2015	1.94 (1.82, 2.07)	2.03 (1.90, 2.17)	2.09 (1.98, 2.21)	2.13 (2.02, 2.26)	2.04 (1.94, 2.15)	2.07 (1.96, 2.19)
Index of multiple deprivation (IMD) quintile	Least deprived	1	1	1	1	1	1
	2	0.78 (0.74, 0.83)	0.81 (0.76, 0.86)	0.79 (0.76, 0.83)	0.83 (0.79, 0.87)	0.79 (0.76, 0.83)	0.83 (0.79, 0.87)
	3	0.68 (0.64, 0.71)	0.73 (0.69, 0.77)	0.71 (0.68, 0.74)	0.78 (0.74, 0.81)	0.70 (0.67, 0.73)	0.76 (0.73, 0.80)
	4	0.54 (0.52, 0.57)	0.64 (0.60, 0.67)	0.58 (0.56, 0.61)	0.69 (0.66, 0.73)	0.58 (0.56, 0.61)	0.69 (0.66, 0.72)
	Most deprived	0.43 (0.41, 0.46)	0.54 (0.51, 0.57)	0.46 (0.44, 0.49)	0.59 (0.56, 0.62)	0.46 (0.44, 0.48)	0.58 (0.55, 0.60)
Region	London	1	1	1	1	1	1
	North East	0.91 (0.81, 1.03)	1.04 (0.92, 1.19)	1.01 (0.90, 1.13)	1.17 (1.05, 1.32)	1.03 (0.93, 1.15)	1.21 (1.08, 1.35)
	North West	1.29 (1.22, 1.36)	1.36 (1.27, 1.44)	1.31 (1.25, 1.38)	1.41 (1.34, 1.49)	1.30 (1.24, 1.36)	1.40 (1.34, 1.48)
	Yorkshire & The Humber	1.51 (1.31, 1.74)	1.54 (1.33, 1.79)	1.48 (1.31, 1.68)	1.55 (1.37, 1.76)	1.44 (1.28, 1.62)	1.53 (1.35, 1.73)
	East Midlands	2.18 (1.69, 2.81)	2.38 (1.84, 3.09)	2.12 (1.70, 2.65)	2.36 (1.88, 2.96)	2.16 (1.75, 2.67)	2.43 (1.96, 3.02)
	West Midlands	1.64 (1.54, 1.75)	1.72 (1.61, 1.84)	1.61 (1.53, 1.70)	1.73 (1.63, 1.83)	1.55 (1.47, 1.63)	1.67 (1.58, 1.76)
	East of England	1.75 (1.63, 1.88)	1.57 (1.46, 1.69)	1.65 (1.55, 1.75)	1.49 (1.40, 1.58)	1.65 (1.56, 1.74)	1.49 (1.41, 1.58)
	South West	1.43 (1.34, 1.52)	1.43 (1.33, 1.52)	1.48 (1.41, 1.56)	1.49 (1.41, 1.57)	1.49 (1.42, 1.57)	1.51 (1.43, 1.59)
	South Central	1.45 (1.37, 1.53)	1.28 (1.21, 1.36)	1.54 (1.47, 1.62)	1.41 (1.34, 1.48)	1.51 (1.44, 1.58)	1.38 (1.32, 1.45)
	South East Coast	1.33 (1.26, 1.41)	1.19 (1.12, 1.26)	1.33 (1.26, 1.39)	1.24 (1.17, 1.30)	1.30 (1.24, 1.36)	1.21 (1.15, 1.27)
Ethnicity	White	1	1	1	1	1	1
	South Asian	0.76 (0.71, 0.81)	0.83 (0.78, 0.88)	0.78 (0.74, 0.83)	0.84 (0.79, 0.88)	0.78 (0.75, 0.82)	0.84 (0.80, 0.89)
	Black	0.45 (0.41, 0.48)	0.61 (0.56, 0.67)	0.46 (0.43, 0.50)	0.61 (0.57, 0.66)	0.47 (0.44, 0.51)	0.63 (0.59, 0.67)
	Mixed	0.65 (0.57, 0.74)	0.72 (0.63, 0.83)	0.64 (0.57, 0.71)	0.70 (0.62, 0.79)	0.63 (0.57, 0.70)	0.69 (0.62, 0.77)
	Other	0.65 (0.59, 0.72)	0.68 (0.62, 0.75)	0.66 (0.61, 0.71)	0.69 (0.63, 0.74)	0.65 (0.61, 0.70)	0.68 (0.63, 0.74)
Maternal age, years	<20	0.79 (0.72, 0.87)	0.81 (0.73, 0.89)	0.73 (0.67, 0.79)	0.73 (0.68, 0.79)	0.74 (0.68, 0.79)	0.74 (0.69, 0.80)
	20-24	1	1	1	1	1	1
	25-29	1.27 (1.21, 1.34)	1.29 (1.22, 1.36)	1.28 (1.23, 1.34)	1.30 (1.25, 1.37)	1.26 (1.21, 1.32)	1.28 (1.23, 1.34)
	30-35	1.54 (1.46, 1.62)	1.55 (1.47, 1.64)	1.55 (1.49, 1.62)	1.57 (1.50, 1.65)	1.54 (1.47, 1.60)	1.55 (1.48, 1.62)
	≥35	1.36 (1.29, 1.44)	1.42 (1.34, 1.51)	1.41 (1.35, 1.48)	1.48 (1.41, 1.55)	1.38 (1.32, 1.44)	1.44 (1.37, 1.51)
Number of children	0	1	1	1	1	1	1
	1	0.67 (0.65, 0.70)	0.65 (0.63, 0.68)	0.69 (0.66, 0.71)	0.67 (0.65, 0.69)	0.70 (0.67, 0.72)	0.68 (0.66, 0.70)
	2	0.47 (0.45, 0.49)	0.47 (0.45, 0.50)	0.50 (0.48, 0.52)	0.49 (0.47, 0.51)	0.50 (0.48, 0.52)	0.50 (0.48, 0.52)
	≥3	0.35 (0.33, 0.37)	0.37 (0.35, 0.40)	0.38 (0.36, 0.40)	0.39 (0.37, 0.42)	0.39 (0.37, 0.41)	0.40 (0.38, 0.42)
Body mass index (BMI)	<18.5	0.71 (0.64, 0.77)		0.69 (0.64, 0.75)		0.71 (0.66, 0.77)	
	18.5-24.9	1		1		1	
	25.0-29.9	1.01 (0.96, 1.05)		0.97 (0.94, 1.01)		0.98 (0.94, 1.01)	
	≥30	0.82 (0.78, 0.86)		0.82 (0.79, 0.85)		0.82 (0.79, 0.85)	

Note: All models include women who registered in first trimester and exclude those with missing ethnicity; minimally adjusted models of BMI excludes women with missing BMI

Abbreviations: UK, United Kingdom; IMD, Index of Multiple Deprivation; BMI, body mass index

Supplementary Table 6: Sensitivity analyses expanding definition of inclusion criteria for the influenza vaccine uptake models: registration by end of pregnancy, including pregnancies without known outcomes, extending influenza season to March, compared to primary analyses

		Primary analyses		Registered by end of pregnancy		Including pregnancies without known outcomes		Extending influenza season through March	
		Minimally adjusted	Fully adjusted	Minimally adjusted	Fully adjusted	Minimally adjusted	Fully adjusted	Minimally adjusted	Fully adjusted
N		140,141	140,141	153,782	153,782	191,950	191,950	140,141	140,141
Season	2010	1	1	1	1	1	1	1	1
	2011	0.89 (0.86, 0.92)	0.89 (0.86, 0.92)	0.89 (0.86, 0.92)	0.89 (0.86, 0.92)	0.92 (0.89, 0.95)	0.92 (0.89, 0.94)	0.93 (0.90, 0.96)	0.93 (0.90, 0.96)
	2012	1.63 (1.58, 1.69)	1.65 (1.60, 1.71)	1.62 (1.57, 1.67)	1.63 (1.58, 1.68)	1.55 (1.51, 1.60)	1.56 (1.52, 1.61)	1.81 (1.76, 1.87)	1.84 (1.78, 1.90)
	2013	1.40 (1.35, 1.45)	1.40 (1.35, 1.45)	1.41 (1.36, 1.45)	1.41 (1.36, 1.46)	1.36 (1.32, 1.41)	1.36 (1.32, 1.41)	1.57 (1.51, 1.62)	1.57 (1.51, 1.62)
	2014	1.69 (1.63, 1.76)	1.70 (1.63, 1.76)	1.71 (1.65, 1.77)	1.72 (1.65, 1.78)	1.64 (1.59, 1.70)	1.63 (1.58, 1.69)	1.88 (1.81, 1.95)	1.89 (1.82, 1.96)
	2015	1.65 (1.58, 1.73)	1.68 (1.60, 1.76)	1.67 (1.60, 1.75)	1.70 (1.63, 1.78)	1.61 (1.54, 1.68)	1.61 (1.55, 1.68)	1.86 (1.78, 1.94)	1.89 (1.81, 1.98)
IMD	Least deprived	1	1	1	1	1	1	1	1
	2	0.90 (0.87, 0.93)	0.92 (0.89, 0.95)	0.88 (0.86, 0.91)	0.90 (0.87, 0.94)	0.87 (0.84, 0.90)	0.89 (0.86, 0.92)	0.90 (0.87, 0.93)	0.91 (0.88, 0.95)
	3	0.84 (0.81, 0.86)	0.88 (0.85, 0.91)	0.83 (0.81, 0.86)	0.87 (0.84, 0.90)	0.83 (0.80, 0.85)	0.87 (0.85, 0.90)	0.83 (0.81, 0.86)	0.87 (0.84, 0.90)
	4	0.71 (0.69, 0.74)	0.76 (0.74, 0.79)	0.71 (0.69, 0.73)	0.76 (0.74, 0.79)	0.71 (0.69, 0.74)	0.78 (0.75, 0.80)	0.70 (0.68, 0.73)	0.75 (0.73, 0.78)
	Most deprived	0.67 (0.65, 0.70)	0.72 (0.70, 0.75)	0.67 (0.65, 0.69)	0.72 (0.69, 0.74)	0.69 (0.67, 0.71)	0.76 (0.73, 0.78)	0.67 (0.65, 0.69)	0.71 (0.69, 0.74)
Region	London	1	1	1	1	1	1	1	1
	North East	1.11 (1.02, 1.21)	1.21 (1.11, 1.31)	1.14 (1.06, 1.24)	1.24 (1.14, 1.34)	1.15 (1.07, 1.24)	1.25 (1.16, 1.35)	1.09 (1.01, 1.18)	1.19 (1.09, 1.29)
	North West	1.37 (1.32, 1.42)	1.42 (1.36, 1.47)	1.39 (1.34, 1.45)	1.44 (1.39, 1.50)	1.42 (1.38, 1.47)	1.48 (1.42, 1.53)	1.38 (1.33, 1.44)	1.44 (1.38, 1.50)
	Yorkshire & The Humber	1.27 (1.18, 1.38)	1.26 (1.16, 1.37)	1.32 (1.22, 1.43)	1.31 (1.21, 1.42)	1.33 (1.24, 1.43)	1.33 (1.23, 1.43)	1.27 (1.17, 1.38)	1.26 (1.17, 1.37)
	East Midlands	1.33 (1.21, 1.47)	1.40 (1.27, 1.55)	1.34 (1.22, 1.47)	1.41 (1.29, 1.55)	1.33 (1.23, 1.45)	1.39 (1.28, 1.52)	1.35 (1.23, 1.49)	1.43 (1.30, 1.58)
	West Midlands	1.41 (1.35, 1.46)	1.43 (1.37, 1.49)	1.42 (1.37, 1.48)	1.45 (1.39, 1.51)	1.43 (1.38, 1.48)	1.45 (1.40, 1.51)	1.47 (1.41, 1.53)	1.50 (1.44, 1.57)
	East of England	1.31 (1.26, 1.37)	1.24 (1.19, 1.30)	1.31 (1.26, 1.37)	1.24 (1.19, 1.30)	1.31 (1.26, 1.36)	1.24 (1.19, 1.29)	1.32 (1.26, 1.37)	1.25 (1.20, 1.31)
	South West	1.25 (1.20, 1.31)	1.25 (1.20, 1.31)	1.29 (1.24, 1.35)	1.29 (1.24, 1.35)	1.34 (1.29, 1.39)	1.34 (1.29, 1.39)	1.27 (1.22, 1.32)	1.27 (1.22, 1.33)
	South Central	1.42 (1.36, 1.47)	1.33 (1.28, 1.38)	1.45 (1.40, 1.50)	1.36 (1.31, 1.41)	1.47 (1.42, 1.52)	1.38 (1.33, 1.43)	1.43 (1.38, 1.48)	1.34 (1.29, 1.40)
	South East Coast	1.06 (1.02, 1.10)	1.02 (0.98, 1.06)	1.07 (1.04, 1.11)	1.03 (0.99, 1.07)	1.11 (1.07, 1.15)	1.07 (1.03, 1.11)	1.05 (1.01, 1.09)	1.01 (0.97, 1.05)
Ethnicity	White	1	1	1	1	1	1	1	1
	South Asian	0.92 (0.88, 0.95)	0.99 (0.95, 1.03)	0.92 (0.88, 0.95)	0.99 (0.95, 1.03)	0.93 (0.90, 0.96)	0.98 (0.95, 1.02)	0.94 (0.91, 0.98)	1.02 (0.98, 1.06)
	Black	0.67 (0.64, 0.71)	0.83 (0.78, 0.88)	0.68 (0.64, 0.71)	0.83 (0.78, 0.88)	0.69 (0.65, 0.72)	0.83 (0.79, 0.87)	0.67 (0.64, 0.71)	0.83 (0.79, 0.88)
	Mixed	0.84 (0.77, 0.92)	0.91 (0.83, 0.99)	0.78 (0.72, 0.85)	0.84 (0.77, 0.92)	0.79 (0.73, 0.86)	0.86 (0.79, 0.93)	0.83 (0.76, 0.91)	0.90 (0.82, 0.99)
	Other	0.79 (0.73, 0.84)	0.85 (0.79, 0.91)	0.75 (0.70, 0.80)	0.81 (0.76, 0.86)	0.77 (0.73, 0.82)	0.83 (0.78, 0.88)	0.80 (0.75, 0.85)	0.86 (0.80, 0.92)
Maternal age, years	<20	0.87 (0.81, 0.92)	0.87 (0.82, 0.93)	0.87 (0.82, 0.93)	0.87 (0.82, 0.93)	0.68 (0.65, 0.72)	0.68 (0.64, 0.71)	0.88 (0.82, 0.93)	0.88 (0.83, 0.94)
	20-24	1	1	1	1	1	1	1	1
	25-29	1.13 (1.09, 1.17)	1.12 (1.08, 1.16)	1.14 (1.10, 1.18)	1.13 (1.10, 1.17)	1.24 (1.20, 1.28)	1.25 (1.21, 1.29)	1.13 (1.09, 1.17)	1.13 (1.09, 1.17)
	30-35	1.22 (1.18, 1.26)	1.21 (1.17, 1.25)	1.24 (1.20, 1.28)	1.23 (1.19, 1.27)	1.36 (1.32, 1.41)	1.38 (1.34, 1.42)	1.23 (1.19, 1.27)	1.22 (1.17, 1.26)
	≥35	1.17 (1.12, 1.21)	1.18 (1.13, 1.22)	1.19 (1.15, 1.23)	1.20 (1.15, 1.24)	1.18 (1.14, 1.21)	1.21 (1.17, 1.25)	1.16 (1.12, 1.21)	1.18 (1.14, 1.23)
	0	1	1	1	1	1	1	1	1

Walker JL, *et al. BMJ Open* 2021; 11:e046545. doi: 10.1136/bmjopen-2020-046545

Number of children	1	0.80 (0.78, 0.82)	0.80 (0.78, 0.82)	0.84 (0.82, 0.86)	0.83 (0.81, 0.85)	0.87 (0.85, 0.89)	0.86 (0.84, 0.88)	0.79 (0.77, 0.80)	0.78 (0.76, 0.80)
	2	0.71 (0.68, 0.73)	0.71 (0.69, 0.74)	0.75 (0.72, 0.77)	0.74 (0.72, 0.77)	0.72 (0.70, 0.75)	0.71 (0.68, 0.73)	0.69 (0.67, 0.71)	0.69 (0.67, 0.72)
	≥3	0.61 (0.58, 0.63)	0.62 (0.59, 0.65)	0.64 (0.61, 0.67)	0.65 (0.62, 0.68)	0.63 (0.61, 0.66)	0.63 (0.60, 0.66)	0.59 (0.56, 0.61)	0.60 (0.57, 0.63)
Clinical risk group	No	1	1	1	1	1	1	1	1
	Yes	1.69 (1.62, 1.76)	1.70 (1.63, 1.77)	1.73 (1.66, 1.80)	1.73 (1.66, 1.80)	1.98 (1.91, 2.06)	2.00 (1.93, 2.07)	1.59 (1.53, 1.66)	1.60 (1.54, 1.67)
BMI	<18.5	0.85 (0.80, 0.90)		0.84 (0.79, 0.89)		0.84 (0.79, 0.88)		0.93 (0.88, 0.98)	
	18.5-24.9	1		1		1		1	
	25.0-29.9	1.04 (1.01, 1.07)		1.03 (1.01, 1.06)		1.04 (1.02, 1.07)		0.98 (0.95, 1.00)	
	≥30	1.00 (0.97, 1.03)		0.99 (0.96, 1.02)		1.03 (1.00, 1.06)		0.90 (0.87, 0.92)	

Note: All models include women who registered in first trimester, and exclude those with outcome unknown and missing ethnicity; minimally adjusted model of BMI excludes women with missing BMI

Abbreviations: UK, United Kingdom; IMD, Index of Multiple Deprivation; BMI, body mass index

Supplementary Table 7: Secondary analysis of subsequent pertussis vaccine uptake among women who had received pertussis vaccination in their first eligible pregnancy and had a second eligible pregnancy within the study period (N=3,111)

		**Total (column %)**	**Received pertussis vaccine in second pregnancy (row %)**	**Minimally adjusted model** OR of receiving vaccine in second pregnancy (95% CI)	**Fully adjusted model** OR of receiving vaccine in second pregnancy (95% CI)
N		3,111	1,877 (60.3)		
Year of first pregnancy	2012	550 (17.7)	380 (69.1)	1	1
	2013	1,912 (61.5)	1,264 (66.1)	0.87 (0.71-1.07)	0.70 (0.56-0.87)
	2014-15	649 (20.9)	233 (35.9)	0.25 (0.20-0.32)	0.14 (0.10-0.18)
Index of multiple deprivation (IMD) quintile	Least deprived	857 (27.6)	539 (62.9)	1	1
	2	539 (17.3)	326 (60.5)	0.90 (0.71-1.13)	0.91 (0.71-1.16)
	3	604 (19.4)	381 (63.1)	1.03 (0.92-1.28)	1.06 (0.83-1.35)
	4	579 (18.6)	337 (58.2)	0.82(0.66-1.02)	0.89 (0.70-1.15)
	Most deprived	532 (17.1)	294 (55.3)	0.72 (0.57-0.90)	0.77 (0.59-1.01)
Region	London	453 (14.6)	260 (57.4)	1	1
	North East	35 (1.1)	22 (62.9)	1.25 (0.65-2.83)	2.08 (0.95-4.58)
	North West	390 (12.5)	240 (61.5)	1.16 (0.87-1.55)	1.29 (0.95-1.77)
	Yorkshire & The Humber	31 (1.0)	14 (45.2)	0.56 (0.27-1.19)	0.73 (0.33-1.62)
	East Midlands	0	0	-	-
	West Midlands	375 (12.1)	229 (61.1)	1.13 (0.85-1.51)	1.33 (0.97-1.81)
	East of England	296 (9.5)	201 (67.9)	1.57 (1.14-2.15)	1.54 (1.10-2.16)
	South West	388 (12.5)	239 (61.6)	1.19 (0.98-1.58)	1.31 (0.96-1.79)
	South Central	562 (18.1)	360 (64.1)	1.33 (1.02-1.73)	1.31 (0.99-1.74)
	South East Coast	581 (18.7)	312 (53.7)	0.90 (0.69-1.16)	0.99 (0.75-1.31)
Ethnicity	White	2,732 (87.8)	1,657 (60.7)	1	1
	South Asian	204 (6.6)	114 (55.9)	0.82 (0.61-1.10)	0.78 (0.57-1.07)
	Black	84 (2.7)	49 (58.3)	1.05 (0.66-1.67)	1.09 (0.66-1.80)
	Mixed	33 (1.1)	20 (60.6)	0.94 (0.46-1.94)	1.14 (0.63-2.07)
	Other	58 (1.9)	37 (63.8)	1.25 (0.71-2.20)	0.97 (0.46-2.06)
Maternal age, years	<20	102 (3.2)	40 (39.2)	0.48 (0.30-0.75)	0.48 (0.30-0.77)
	20-24	505 (16.2)	290 (57.4)	1	1
	25-29	1,002 (32.2)	592 (59.1)	1.07 (0.85-1.34)	1.09 (0.86-1.39)
	30-34	1,048 (33.7)	669 (63.8)	1.32 (1.05-1.65)	1.26 (0.98-1.61)
	≥35	454 (14.6)	286 (63.0)	1.29 (0.99-1.69)	1.26 (0.94-1.69)
Number of children	0	1,936 (62.2)	1,224 (63.2)	1	1
	1	714 (23.0)	405 (56.7)	0.78 (0.65-0.94)	0.75 (0.62-0.91)
	2	264 (8.5)	149 (56.4)	0.72 (0.55-0.95)	0.64 (0.48-0.85)
	≥3	197 (6.3)	99 (50.3)	0.56 (0.42-0.76)	0.50 (0.36-0.69)
Pregnancy interval (days from end of first pregnancy to start of second)	0-179	416 (13.4)	227 (54.6)	1	1
	180-359	749 (24.1)	476 (63.6)	1.25 (0.96-1.63)	1.11 (0.85-1.45)
	360-539	1,004 (32.3)	695 (69.2)	1.33 (1.03-1.71)	1.13 (0.86-1.47)
	540-719	624 (20.1)	373 (59.8)	0.65 (0.49-0.85)	0.54 (0.41-0.73)
	720+	318 (10.2)	106 (33.3)	0.19 (0.14-0.27)	0.16 (0.11-0.22)

Note: Among 3,363 women with two eligible pregnancies during follow up, excluded 2 with implausible (<0 days) spacing between the end of the first pregnancy and start of the second, and 250 with missing ethnicity data.

Walker JL, *et al. BMJ Open* 2021; 11:e046545. doi: 10.1136/bmjopen-2020-046545
