# Supplementary material for: Social determinants of pertussis and influenza vaccine uptake in pregnancy: a national cohort study in England using electronic health records
Source: BMJ Open. 2021 Jun 21;11(6):e046545. doi: 10.1136/bmjopen-2020-046545 (PMC8217954; doi:10.1136/bmjopen-2020-046545)
Supplement: Author's manuscript [file bmjopen-2020-046545.draft_revisions.pdf]

BMJ Open is committed to open peer review. As part of this commitment we make the peer review history of every article we publish publicly available.

When an article is published we post the peer reviewers' comments and the authors' responses online. We also post the versions of the paper that were used during peer review. These are the versions that the peer review comments apply to.

The versions of the paper that follow are the versions that were submitted during the peer review process. They are not the versions of record or the final published versions. They should not be cited or distributed as the published version of this manuscript.

BMJ Open is an open access journal and the full, final, typeset and author-corrected version of record of the manuscript is available on our site with no access controls, subscription charges or pay-per-view fees (http://bmjopen.bmj.com).

If you have any questions on BMJ Open's open peer review process please email info.bmjopen@bmj.com

BMJ Open

Social determinants of pertussis and influenza vaccine uptake in pregnancy: a national cohort study using electronic health records

Journal:	*BMJ Open*
Manuscript ID	bmjopen-2020-046545
Article Type:	Original research
Date Submitted by the Author:	04-Nov-2020
Complete List of Authors:	Walker, Jemma; London School of Hygiene and Tropical Medicine, Faculty of Epidemiology and Population Health; Public Health England, Statistics, Modelling and Economics Department<br>Rentsch, Christopher ; London School of Hygiene & Tropical Medicine, Faculty of Epidemiology and Population Health<br>McDonald, Helen; London School of Hygiene and Tropical Medicine, Faculty of Epidemiology and Population Health<br>Bak, JeongEun; London School of Hygiene and Tropical Medicine, Faculty of Epidemiology and Population Health<br>Minassian, Caroline ; London School of Hygiene & Tropical Medicine, Faculty of Epidemiology and Population Health<br>Amirthalingam, Gayatri; Public Health England Immunisation and Countermeasures Division<br>Edelstein, Michael; Public Health England Immunisation and Countermeasures Division; London School of Hygiene & Tropical Medicine, Faculty of Epidemiology and Population Health<br>Thomas, Sara; London School of Hygiene & Tropical Medicine, Faculty of Epidemiology & Population Health
Keywords:	Public health < INFECTIOUS DISEASES, Maternal medicine < OBSTETRICS, PRIMARY CARE, PUBLIC HEALTH

SCHOLARONE™
Manuscripts

I, the Submitting Author has the right to grant and does grant on behalf of all authors of the Work (as defined in the below author licence), an exclusive licence and/or a non-exclusive licence for contributions from authors who are: i) UK Crown employees; ii) where BMJ has agreed a CC-BY licence shall apply, and/or iii) in accordance with the terms applicable for US Federal Government officers or employees acting as part of their official duties; on a worldwide, perpetual, irrevocable, royalty-free basis to BMJ Publishing Group Ltd ("BMJ") its licensees and where the relevant Journal is co-owned by BMJ to the co-owners of the Journal, to publish the Work in this journal and any other BMJ products and to exploit all rights, as set out in our licence.

The Submitting Author accepts and understands that any supply made under these terms is made by BMJ to the Submitting Author unless you are acting as an employee on behalf of your employer or a postgraduate student of an affiliated institution which is paying any applicable article publishing charge ("APC") for Open Access articles. Where the Submitting Author wishes to make the Work available on an Open Access basis (and intends to pay the relevant APC), the terms of reuse of such Open Access shall be governed by a Creative Commons licence – details of these licences and which Creative Commons licence will apply to this Work are set out in our licence referred to above.

Other than as permitted in any relevant BMJ Author's Self Archiving Policies, I confirm this Work has not been accepted for publication elsewhere, is not being considered for publication elsewhere and does not duplicate material already published. I confirm all authors consent to publication of this Work and authorise the granting of this licence.

For peer review only

Social determinants of pertussis and influenza vaccine uptake in pregnancy: a national cohort study using electronic health records

Authors: Jemma L Walker (0000-0003-3728-9509),[1,2,3*] Christopher T Rentsch (0000-0002-1408-7907),[1,2*] Helen I McDonald (0000-0003-0576-2015),[1,2§] JeongEun Bak (0000-0001-7519-7539),[2] Caroline Minassian (0000-0001-9406-1928),[2] Gayatri Amirthalingam (0000-0003-2078-0975),[1,4] Michael Edelstein (0000-0002-7323-0806),[1,2,4] Sara L Thomas.[1,2]

*Joint first authorship

1. NIHR Health Protection Research Unit in Immunisation
2. Faculty of Epidemiology and Population Health, London School of Hygiene and Tropical Medicine, London, WC1E 7HT, UK
    a. Jemma L Walker research fellow, Christopher T Rentsch research fellow, Helen I McDonald clinical research fellow, JeongEun Bak MSc student, Caroline Minassian assistant professor, Michael Edelstein honorary associate professor, Sara L Thomas professor
3. Statistics, Modelling and Economics Department, Public Health England, London, NW9 5EQ, UK
    a. Jemma L Walker statistician
4. Immunisation and Countermeasures Division, National Infection Service, Public Health England, London, NW9 5EQ, UK
    a. Gayatri Amirthalingam consultant epidemiologist, Michael Edelstein consultant epidemiologist

§Corresponding author:
Helen I McDonald, PhD
London School of Hygiene & Tropical Medicine
Keppel Street
WC1E 7HT
London, UK
Email: Helen.McDonald@lshtm.ac.uk

Article type: Research
Keywords: Influenza vaccine; pertussis vaccine; vaccination coverage; social determinants of health; electronic health records.

Word count: 3,896

For peer review only - http://bmjopen.bmj.com/site/about/guidelines.xhtml

ABSTRACT

Objective To examine the social determinants of influenza and pertussis vaccine uptake among pregnant women in England.

Design Nationwide population-based cohort study

Setting The study used anonymised primary care data from the Clinical Practice Research Datalink and linked Hospital Episode Statistics secondary care data

Participants Pregnant women eligible for pertussis (2012 to 2015, n=68,090) or influenza (2010/11 to 2015/16, n=152,132) vaccination, 2012 to 2015 (pertussis) and 2010/11 to 2015/16 (influenza)

Main outcome measures Influenza and pertussis vaccine uptake

Results

Vaccine uptake in the first eligible pregnancy was 67.3% for pertussis, and 39.1% for influenza. Uptake of both vaccines varied by region, with lowest uptakes in London and the North East. Lower vaccine uptake was associated with greater deprivation: almost 10% lower in the most deprived quintiles compared with the least deprived for influenza (44.0% vs 34.5%), and almost 20% lower for pertussis (76.0% vs 57.7%). Lower uptake for both vaccines was also associated with non-white ethnicity (lowest among women of Black ethnicity), maternal age under 20 years, and a greater number of children in the household. The associations between all social factors and vaccine uptake were substantially unchanged in fully adjusted models, suggesting the social determinants of uptake were largely independent of one another.

For peer review only

For peer review only - http://bmjopen.bmj.com/site/about/guidelines.xhtml

Among 3,111 women vaccinated against pertussis in their first eligible pregnancy and pregnant again, 1,234 (40%) were not vaccinated in their second eligible pregnancy.

Conclusions

Targeting promotional campaigns to pregnant women who are younger, of non-white ethnicity, with more children, living in areas of greater deprivation or the London or North East regions, has potential to reduce vaccine-preventable disease among infants and pregnant women, and to reduce health inequalities. Vaccination promotion needs to be sustained across successive pregnancies. Further research is needed into whether the effectiveness of vaccine promotion strategies may vary according to social factors.

For peer review only

Article Summary

Strengths and limitations of this study

- This large cohort study explored the social determinants of influenza and pertussis vaccination among pregnant women across England
- It considered a range of social determinants including maternal age, ethnicity, socio-economic status, number of children in the household and region.
- The CPRD/LSHTM pregnancy register was used to ascertain pregnancies and their timing from primary care records using detailed algorithms
- The study is not able to distinguish inequalities in vaccine uptake according to different settings such as secondary care maternity services

For peer review only

For peer review only - http://bmjopen.bmj.com/site/about/guidelines.xhtml

INTRODUCTION

Pertussis (whooping cough) and seasonal influenza can have severe outcomes among pregnant women and young infants, including hospitalisation and death.[1-3] A pertussis outbreak in 2012 resulted in 14 infant deaths, most of whom were too young to be vaccinated directly.[4] Vaccination in pregnancy reduces influenza-associated hospitalisation among pregnant women,[5] and provides 'passive immunity' to protect infants in the first months of life.[6, 7] In England, pertussis vaccination has been offered to women in later stages of pregnancy since 2010 and seasonal influenza vaccination at any stage of pregnancy during influenza season since 2012.[4, 8]

Low vaccine uptake during pregnancy is a major public health challenge for high-income countries.[9] According to routine surveillance in 2018/19, vaccine uptake amongst pregnant women in England was 68.8% for pertussis and 45.2% for influenza.[10, 11] Although comparatively high for a high-income country, this suboptimal uptake still limits the programme's impact and results in vaccine-preventable deaths among infants of unvaccinated mothers. Studies of determinants of maternal influenza vaccine uptake to date have largely focused on health beliefs,[12] but less is known about the role of social factors. During the 2009 influenza pandemic, higher vaccine uptake in pregnancy was associated with higher maternal age, previous deliveries, and underlying health conditions but not deprivation.[13] However, ecological studies suggest that both seasonal influenza and pertussis vaccine uptake in pregnancy vary with ethnicity, and are lower in areas with greater deprivation, and are thus sources of health inequalities in infancy.[14, 15] Smaller studies of pertussis and seasonal influenza vaccines have suggested deprivation, ethnicity, maternal age and parity or number of children may be factors in maternal

For peer review only

vaccine uptake, but have lacked power to describe these associations fully.[16-20] A better understanding of the social determinants of maternal vaccine uptake could inform targeted public health interventions to improve vaccine uptake and reduce health inequalities.

This study aimed to use linked electronic health records to examine the social determinants of influenza and pertussis vaccine uptake among pregnant women in England from programme introduction to 2015 (pertussis) or the 2015/16 influenza season (influenza).

For peer review only

For peer review only - http://bmjopen.bmj.com/site/about/guidelines.xhtml

METHODS

Data sources

This historical cohort study used data from the Clinical Practice Research Datalink (CPRD), a quality-assured anonymised primary care patient dataset covering approximately 7% of general practices in England.[21, 22] Available data include diagnoses and symptoms, prescriptions, immunisations and referrals recorded in primary care. The CPRD/LSHTM Pregnancy Register details all pregnancies recorded in primary care, identified using detailed algorithms to determine their timing and outcomes.[23] For this analysis, we used the Pregnancy Register and CPRD data pre-linked to Hospital Episode Statistics (HES) admissions data (for supplementary ethnicity data),[24] and Office of National Statistics (ONS) small-area-level deprivation data.[25]

Study population

Analysis of pertussis vaccine and seasonal influenza vaccine uptake were conducted separately. For each vaccine, we identified pregnancies eligible for the relevant vaccination among women registered with CPRD, using the Pregnancy Register to identify start and end dates of pregnancies, eligible dates based on gestation, and pregnancy outcomes. Eligible women were registered at one of the 75% of CPRD practices in England which participate in the CPRD data-linkage scheme, for availability of linked HES and ONS data.[21] Vaccine eligibility started on or after 1 October 2012 for the pertussis vaccine analyses, and on or after 1 April 2010 for the seasonal influenza vaccine analyses, reflecting the introduction of vaccination

For peer review only - http://bmjopen.bmj.com/site/about/guidelines.xhtml

programmes.[4, 8] For each vaccine, the first eligible pregnancy for each woman during the follow-up period was used to avoid non-independence in the data.

Vaccination guidelines during the study period suggested women be offered pertussis vaccination in their third trimester of pregnancy (ideally between 28-32 weeks, though it could be given up to delivery).[4, 8] The study period ended before the April 2016 change in guidelines recommending vaccination at 16-32 weeks of pregnancy, or changes in the commissioning arrangements leading to increased delivery through maternity services from 2016.[4] For the pertussis vaccine analyses, we included women who delivered a live-or stillborn child on or after 26 weeks of pregnancy, which allowed for up to 2 weeks imprecision in the Pregnancy Register estimation of third trimester and mirrored the national surveillance approach.

Influenza vaccination is recommended at any stage in pregnancy that overlaps with the influenza season.[8] For the influenza vaccine analyses, all pregnancies for which the Pregnancy Register included a known outcome (such as stillbirth, livebirth, miscarriage, or termination) were included, irrespective of duration of pregnancy, providing the pregnancy overlapped by at least one day with the influenza season (1 September to 31 January of each year).

We limited primary analyses for both maternal vaccines to women who registered as patients at the primary care practice by the end of their first trimester, to reduce misclassification of vaccination status. We conducted sensitivity analyses around the study inclusion criteria, which are described below.

Follow-up period

The study period ranged from 1 October 2012 to 30 September 2015 for pertussis vaccine and 1 September 2010 to 31 January 2016 for influenza vaccine. Start of

For peer review only

For peer review only - http://bmjopen.bmj.com/site/about/guidelines.xhtml

follow-up was considered the latest date of: start of the study period, practice meeting CPRD quality standards, patient registration at the practice, 11th birthday (dates of birth based on the mid-point of year of birth), 26 weeks gestation of pregnancy (for pertussis), the start of pregnancy plus 2 weeks (for influenza), or 1st September of each year (for influenza). End of follow-up was the earliest date of: last data collection from the practice, end of linkage to HES, patient transfer out of the practice, 49th birthday, death, receipt of the vaccine of interest, the 40th week of pregnancy (for pertussis), end of pregnancy (for influenza), end of the study period, or 31 January of each year (for influenza).

Vaccine uptake

Vaccination status for both maternal pertussis and influenza vaccines was extracted from CPRD. For the primary analysis of pertussis vaccine uptake, women were considered vaccinated if they received the vaccine between 26 and 40 weeks of pregnancy gestation, which is similar to the national vaccination guidelines of 28 to 38 weeks but allows for up to two weeks discrepancy in the Pregnancy Register estimation of gestation. For the primary analysis of influenza vaccine uptake, women were considered vaccinated if they received the vaccine on any day between 1 September and 31 January during their follow-up period. Women with a pregnancy that spanned two influenza seasons (n=19,963, 14%) were counted in the denominator of the latter season and considered vaccinated if vaccinated in either season.

Social characteristics and clinical conditions

For peer review only - http://bmjopen.bmj.com/site/about/guidelines.xhtml

We defined social determinants using previously published detailed algorithms.[26] Index of multiple deprivation (IMD, a composite measure of relative deprivation) was assigned in quintiles (1 representing least deprived, 5 most deprived) based on the Lower Super Output Area of the patient's residential address using ONS national statistics data.[25] Ethnicity (White, South Asian, Black, Mixed, Other) was defined using primary care records supplemented with linked HES data.[24] Other social factors of interest were defined using CPRD primary care data and comprised: region of residence (London, North East, North West, Yorkshire & The Humber, East Midlands, West Midlands, East of England, South West, South Central, and South East Coast), maternal age (based on midpoint of year of birth), and number of children in the household.

For influenza vaccine uptake analyses, whether the individual was in a clinical risk group indicated to receive influenza vaccine was defined according to national guidance,[8] and comprised the following conditions: chronic renal disease, chronic heart disease, chronic respiratory disease, chronic liver disease, diabetes, immunosuppression, chronic neurological disease, asplenia, and morbid obesity. Clinical risk groups were identified using Read codes, primary care prescription records (for immunosuppression and asthma), and height and weight records. Body mass index (BMI) was defined using height and weight records using validated methods,[27] and defined based on the record closest to the beginning of pregnancy, allowing measures during the first trimester of pregnancy. Asthma was defined as an asthma diagnosis and either any history of an emergency hospital admission for asthma, or any inhaled or oral steroid prescription in the previous 12 months. The algorithms used for immunosuppression are described in previous studies;[28]

For peer review only - http://bmjopen.bmj.com/site/about/guidelines.xhtml

codelists for other conditions are available from https://doi.org/10.17037/DATA.00001907.

Statistical analysis

Parallel analyses were conducted for pertussis and influenza vaccine uptake. For each vaccine, a complete case analysis (excluding women with no ethnicity recorded in the main analysis) using multivariable logistic regression was used to estimate associations between vaccine uptake and social determinants. Our modelling strategy followed a previously adapted version[29] of a conceptual framework to analyse the hierarchical inter-relationships between distal and proximate social determinants with vaccine uptake **(Supplementary Table 1)**.[30] We first fitted a 'minimally adjusted' model to estimate associations between each social determinant and vaccine uptake adjusted for year (calendar year for pertussis, financial year for influenza to reflect the influenza season) to adjust for secular trends as an *a priori* confounder. We then fitted five further sequential models. Models 1 to 3 explored the social determinants of uptake from distal to proximal. Model 4 and the BMI Model explored the extent to which these were mediated by clinical conditions (for influenza), and mediated and/or confounded by BMI (for both vaccines).

In Model 1 we assessed associations between vaccine uptake and the distal determinants IMD, region, and ethnicity, mutually adjusted and adjusted for year. In Model 2 the intermediate variable maternal age was added alongside the variables in Model 1 to determine to what extent this explained any effect of the distal variables. Model 3 comprised the variables in Model 2 and the proximate variable number of children, to investigate whether this mediated the effect of the distal and intermediate variables. For influenza uptake modelling, we further added clinical risk group as a

For peer review only - http://bmjopen.bmj.com/site/about/guidelines.xhtml

potential mediator of the social characteristics (Model 4). Finally, we repeated complete case analyses additionally excluding women with no recorded BMI for all four models, adding a further model (BMI Model) that additionally adjusted for BMI, which may both mediate and confound the effect of social characteristics and clinical conditions.

All analyses were conducted using Stata 15 (StataCorp, College Station, TX, USA).

Missing data and sensitivity analyses

Primary analyses were conducted on women who had non-missing ethnicity and who were registered with an up-to-standard CPRD practice by the end of their first trimester. Other than ethnicity, only BMI had missing data.

We performed descriptive and sensitivity analyses to understand how estimates of vaccine uptake and associations with social determinants might be affected by missing data or study inclusion criteria. First, we examined the distribution of social determinants among women with and without recorded ethnicity. Second, we compared estimates from minimally and fully adjusted models from the primary analyses with sensitivity analyses including women who registered with an up-to-standard practice by the end of pregnancy (instead of end of first trimester) for both vaccines. For the pertussis analyses, we further ran minimally and fully adjusted models that mirrored national surveillance criteria of immunisation at 28-38 weeks' gestation, to assess the impact of allowing a two-week window for imprecise estimation of gestation in our primary analysis. For the influenza analyses, we further ran models that included pregnancies with no recorded outcome, as well as models that extended the influenza season through 31 March of each year. Finally, for both

For peer review only - http://bmjopen.bmj.com/site/about/guidelines.xhtml

pertussis and influenza analyses, we fitted random effects models to test for clustering by general practice.

Secondary analysis of sequential pregnancies

In response to the finding that vaccine uptake declined with greater number of children in the household, a *post-hoc* secondary analysis was added investigating the social determinants associated with vaccination in a second eligible pregnancy among women who had received pertussis vaccination in their first eligible pregnancy. This analysis focused on pertussis vaccination, as influenza vaccination uptake may depend upon the extent and timing of the overlap of pregnancy with the influenza season, severity of the influenza season and timing of vaccine availability, reducing the number of eligible sequential pregnancies and increasing the complexity of external factors which may affect a women's vaccine uptake across sequential pregnancies. Logistic regression with likelihood ratio tests were used to model and test minimally adjusted and fully adjusted (Model 3) associations between the outcome (vaccination in the second eligible pregnancy) and social determinants measured at baseline of the first eligible pregnancy, as well as additionally adjusting for the time interval between the end of the first pregnancy and the start of the next.

Ethics

The study was approved by the Independent Scientific Advisory Group of the CPRD (ISAC, Reference: 17_030) with an amendment to include the secondary analysis (ISAC reference 17_030RA2) and the London School of Hygiene and Tropical

For peer review only - http://bmjopen.bmj.com/site/about/guidelines.xhtml

Medicine Ethics Committee (Reference: 16265). The amended ISAC protocol was made available to reviewers.

Patient and public involvement

Findings from this study were discussed at a public engagement event to inform priorities for future research by the NIHR Health Protection Research Unit in Immunisation.

For peer review only

For peer review only - http://bmjopen.bmj.com/site/about/guidelines.xhtml

RESULTS

Sample characteristics

A total of 68,090 women from 402 general practices and 152,132 from 456 general practices were eligible for uptake of the pertussis and influenza vaccine, respectively, during the study period. Many women were eligible to be offered both pertussis and influenza vaccinations during the study: 66,143 women were included in both analytic samples. There were 5,553 (8.9%) and 11,991 (7.9%) women from the pertussis and influenza vaccine analyses, respectively, who had missing ethnicity and were excluded from analysis. Compared to women with recorded ethnicity, women with missing ethnicity were more likely to have an eligible pregnancy later in the study period, reside in South Central or South East Coast regions of England, have no children living in their household, and to have missing BMI information (all p<0.001, **Supplementary Table 2**). Vaccine uptake was similar between women with recorded versus missing ethnicity for pertussis (67.3% vs. 68.2) and influenza (39.1% vs. 40.4%).

Primary analyses – pertussis vaccination

Among 62,537 eligible women with recorded ethnicity, maternal pertussis vaccine uptake increased each year, reaching 71.7% in 2015 (**Table 1**). Uptake was also highest in the least deprived areas (76.0%) and East and West Midlands (74.5% and 72.9%, respectively), and among women of white ethnicity (69.0%), aged 30-35 years (70.8%), who had no other children living in household (74.4%), who were of normal weight or overweight (69.2% and 69.3%, respectively).

For peer review only - http://bmjopen.bmj.com/site/about/guidelines.xhtml

After adjusting for calendar year, those who resided in the most deprived areas had less than half the odds of vaccine uptake compared to those in the least deprived areas, and those in all regions of England apart from the North East had increased odds of uptake compared to London (**Table 1**). Pertussis vaccination uptake was appreciably lower among all non-white ethnic groups, with reduced odds of between 24% (South Asian) and 55% (Black ethnicity) compared to those of White ethnicity. The odds of vaccination increased non-linearly with maternal age; compared to women aged 20-24 years, women who were <20 years had 21% lower odds of receiving vaccination and there was an increased likelihood of vaccination among women aged ≥25 years, reaching 54% increased odds of uptake among those aged 30-35 years. Uptake decreased linearly with increasing numbers of children living in the household; 33% less likely among women with one child, 53% less likely among women with two children, and 65% less likely among women with three or more children (Table 1). Among the 55,871 women with available BMI data, calendar-year adjusted uptake was 29% less likely among women whose BMI was classified as underweight and 18% less likely among women classified as obese, compared to women with normal BMI (**Table 1**).

Associations in the minimally adjusted models were largely unchanged after additionally adjusting for IMD, region, and ethnicity (Model 1), maternal age (Model 2), and number of children (Model 3). Associations were slightly attenuated (>10% change) for some regions in England (i.e., East of England, South Central, and South East Coast) in Model 1 and Model 2, but not in Model 3. Similarly, associations of pertussis uptake were marginally attenuated in non-white ethnic groups by adjustment for IMD and region (Model 2). However, strong evidence of all these associations remained. Model estimates were also robust to the additional

For peer review only - http://bmjopen.bmj.com/site/about/guidelines.xhtml

adjustment for BMI in the subset of women with non-missing BMI (**Supplementary Table 3**).

Primary analyses – influenza vaccination

Similar to pertussis vaccination, maternal influenza vaccine uptake was highest (46%) by the end of the study period (the 2015/16 season) among the 140,141 eligible women with recorded ethnicity (**Table 2**). Uptake was also highest in the least deprived areas (44.0%), in the South Central and West Midlands regions (42.6% and 42.2%, respectively), and among women of white ethnicity (39.8%), aged 30-35 years (41.0%), who had no children living in household (43.0%), and who were overweight (40.4%). Women who were classified as being in a clinical risk group had the highest influenza vaccine uptake (50.9%) out of all subgroups.

Findings of associations between social determinants and influenza vaccine uptake were largely the same as those with pertussis uptake (**Table 2**). Women were 65% more likely to receive the influenza vaccination in the 2015/16 season compared to the 2010/11 season. Similarly, in influenza-season adjusted models, women who resided in the most deprived areas had 29% lower odds of receiving vaccination, and women in all regions outside of London were more likely to be vaccinated. Associations with ethnicity, maternal age, number of children, and BMI also mirrored those found in the pertussis uptake models, although the lower uptake seen with women of non-white ethnicity was less marked than that seen for pertussis vaccination. Women identified as being in a clinical risk group for influenza were 69% more likely to be vaccinated than those not in a clinical risk group. Associations were robust throughout all subsequent models except for South Asian ethnicity and

For peer review only - http://bmjopen.bmj.com/site/about/guidelines.xhtml

For peer review only

South East Coast regional residence, and remained after additional adjustment for clinical risk group in Model 4 (**Table 2**). Model estimates were also robust to the additional adjustment for BMI in the model excluding those with missing BMI (**Supplementary Table 4**).

Sensitivity analyses

For the pertussis uptake analysis, associations and conclusions from the primary analysis remained the same after altering study inclusion criteria to include women who registered at any point during pregnancy, and mirroring the national surveillance criteria of immunisation at 28-38 weeks' gestation (**Supplementary Table 5**).

As with the pertussis sensitivity analysis, there were no changes in influenza uptake effect estimates when altering study inclusion criteria to include women who registered at any point during pregnancy (**Supplementary Table 6**). However, in analyses that included pregnancies with no recorded outcomes, younger women aged <20 years were even less likely to receive influenza vaccination than in primary analysis (OR 0.68, 95% CI 0.64, 0.71 in sensitivity analysis vs. OR 0.87, 95% CI 0.82, 0.93 in primary analysis). Conversely, women aged 25-35 years or those identified as being in a clinical risk group were even more likely to be vaccinated than in primary analysis. In another sensitivity analysis, extending the influenza season through 31 March resulted in greater associations between season and vaccine uptake. Nevertheless, conclusions made from models across all sensitivity analyses were largely the same as those made from the primary analysis. Finally, we found no evidence of clustering at the practice level in the primary analysis models

For peer review only - http://bmjopen.bmj.com/site/about/guidelines.xhtml

for either pertussis or influenza uptake (ρ=0.07, 95% CI 0.06-0.09 for pertussis, ρ=0.03, 95% CI 0.03-0.03 for influenza).

Secondary analysis

Among women who were included in the main study, there were 3,111 women who received pertussis vaccination in their first eligible pregnancy and who completed a second eligible pregnancy within the study period. Among these, 1,234 (39.7%) were not vaccinated in their second eligible pregnancy. Social determinants of vaccine uptake among women who had previously received vaccination in pregnancy were similar to those in the main analysis, with lower uptake in the second eligible pregnancy associated with younger maternal age at the first pregnancy, a greater number of children in the household and a longer interval between pregnancies (**Supplementary Table 7**).

For peer review only

For peer review only - http://bmjopen.bmj.com/site/about/guidelines.xhtml

DISCUSSION

Vaccine uptake in pregnancy over the study period was 67.3% for pertussis and 39.1% for influenza. Lower vaccine uptake was associated with greater deprivation: the gap in uptake between the least and most deprived quintiles was almost 10% for influenza, and almost 20% for pertussis. Lower uptake was also associated with non-white ethnicity (particularly Black ethnicity), maternal age under 20 years, and greater number of children in the household. The associations between all social factors and vaccine uptake were largely independent of one another. Among women eligible for pertussis vaccination in two pregnancies and vaccinated in the first, 40% were not vaccinated in their second eligible pregnancy.

Strengths of this study include the use of the CPRD/LSHTM Pregnancy Register with linked hospital and mortality data and detailed algorithms to identify pregnancy timings and a range of individual-level social determinants among a nationally representative population.[26] Key limitations include low representation from some regions (in particular the East Midlands), and that not all potentially relevant social factors were available, such as education and religion. Our study was also limited to vaccination recorded in primary care settings. Maternity-led vaccination services were rare before 2016, and GPs are required to document vaccinations given outside the surgery. To minimise misclassification we ended our study period prior to the introduction of pertussis vaccination in antenatal settings, but we may have slightly under-estimated influenza vaccine uptake if vaccinations in maternity-led services were incompletely recorded in primary care. Further research is needed to explore whether social determinants of vaccine uptake differ for alternative settings such as antenatal care.

For peer review only - http://bmjopen.bmj.com/site/about/guidelines.xhtml

To our knowledge, this is the first large study of fully individual-level social determinants of maternal vaccine uptake of seasonal influenza and pertussis in England. Our findings differ from a large national study which found no association between deprivation and pandemic influenza vaccine uptake in pregnancy (although vaccine uptake did increase with maternal age) but the previous study was in the context of the 2010 influenza pandemic.[13] The regional variation we observed is reassuringly consistent with national surveillance and ecological studies.[10, 11, 14, 15] For seasonal influenza and pertussis vaccines, previous studies have generally suggested associations consistent with those we observed for deprivation, ethnicity, maternal age and parity or number of children, but studies have been ecological or pseudo-individualised, or were underpowered for precise estimates.[14-18, 20] Our findings in a large and nationally representative dataset demonstrate that each of these factors is an independent individual-level determinant of maternal vaccine uptake, outside of a pandemic context.

The novel finding that 40% of women who had been vaccinated in their first eligible pregnancy were not in their second suggests that low vaccine uptake in pregnancy is not fully determined by fixed maternal attitudes to vaccination. The drop-off in uptake is not explained by number of children in the household, and could suggests a need for awareness-raising of the rationale for passive immunisation of infants and the need for vaccination in each pregnancy.

The large differences in vaccine uptake by deprivation and ethnicity indicate a key opportunity to reduce health inequalities. Further research is needed into interventions to reduce inequalities in vaccine uptake,[31] to ensure that future vaccine promotion narrows rather than widens the large and multi-faceted health inequalities in maternal vaccine uptake. Targeting interventions and improving access to

For peer review only - http://bmjopen.bmj.com/site/about/guidelines.xhtml

vaccines through primary care and maternity services for pregnant women who live in more deprived areas, are of non-white ethnicity, younger, or have more children may reduce health inequalities, improve overall vaccine uptake, and reduce vaccine-preventable deaths among women and children.

For peer review only

For peer review only - http://bmjopen.bmj.com/site/about/guidelines.xhtml

Acknowledgements

This work uses data provided by patients and collected by the NHS as part of their care and support and would not have been possible without access to this data. The NIHR recognises and values the role of patient data, securely accessed and stored, both in underpinning and leading to improvements in research and care.

Author Contributions

JLW and SLT conceived the main study, and CTR and HIM conceived the secondary analysis. JLW, CTR, HIM, CM, and SLT designed the study. JLW performed the data extraction and JLW and CTR performed the statistical analyses. JB, CTR and HIM designed the secondary analysis, for which JB and HIM performed the statistical analysis. All authors contributed to the interpretation of results. CTR and HIM drafted the manuscript, which all authors contributed to, revised critically, and approved. HIM is the guarantor. The corresponding author (JLW) attests that all listed authors meet authorship criteria and that no others meeting the criteria have been omitted.

Funding

The research was funded by the National Institute for Health Research (NIHR) Health Protection Research Unit (HPRU) in Immunisation at the London School of Hygiene and Tropical Medicine in partnership with Public Health England (PHE) (grant number HPRU-2012-10096). The views expressed are those of the authors and not necessarily those of the NHS, the NIHR, the Department of Health and Social Care, or PHE.

For peer review only - http://bmjopen.bmj.com/site/about/guidelines.xhtml

Competing interests

All authors have completed the ICMJE uniform disclosure form at www.icmje.org/coi_disclosure.pdf and declare: JLW, CTR, HIM and SLT had financial support from the National Institute for Health Research (NIHR) Health Protection Research Unit (HPRU) in Immunisation for the submitted work; Public Health England Immunisation and Countermeasures Division has provided vaccine manufacturers with post-marketing surveillance reports on pneumococcal and meningococcal infection which the companies are required to submit to the UK Licensing Authority in compliance with their Risk Management Strategy, and a cost recovery charge is made for these reports; no other relationships or activities that could appear to have influenced the submitted work.

Ethics approval

The study was approved by the Independent Scientific Advisory Group of the CPRD (ISAC reference 17_030RA2) and the London School of Hygiene and Tropical Medicine Ethics Committee (LSHTM reference 16265). The study protocol was made available to reviewers.

Data sharing

The data used for this study were obtained from the Clinical Practice Research Datalink (CPRD). All data are available via an application to the Independent

For peer review only - http://bmjopen.bmj.com/site/about/guidelines.xhtml

Scientific Advisory Committee (see https://www.cprd.com/Data-access). Data acquisition is associated with a fee.

Transparency

The manuscript's guarantor (HIM) affirms that the manuscript is an honest, accurate, and transparent account of the study being reported; that no important aspects of the study have been omitted; and that any discrepancies from the study as planned have been explained.

Copyright/license

The Corresponding Author has the right to grant on behalf of all authors and does grant on behalf of all authors, a non-exclusive worldwide licence to the Publishers and its licensees in perpetuity, in all forms, formats and media (whether known now or created in the future), to i) publish, reproduce, distribute, display and store the Contribution, ii) translate the Contribution into other languages, create adaptations, reprints, include within collections and create summaries, extracts and/or, abstracts of the Contribution, iii) create any other derivative work(s) based on the Contribution, iv) to exploit all subsidiary rights in the Contribution, v) the inclusion of electronic links from the Contribution to third party material where-ever it may be located; and, vi) licence any third party to do any or all of the above.

For peer review only

References

1. van Hoek AJ, Campbell H, Amirthalingam G, Andrews N, Miller E. The number of deaths among infants under one year of age in England with pertussis: results of a capture/recapture analysis for the period 2001 to 2011. Euro Surveillance: Bulletin Europeen sur les Maladies Transmissibles = European Communicable Disease Bulletin. 2013;18(9)
2. Amirthalingam G, Gupta S, Campbell H. Pertussis immunisation and control in England and Wales, 1957 to 2012: a historical review. Euro Surveillance: Bulletin Europeen sur les Maladies Transmissibles = European Communicable Disease Bulletin. 2013;18(38)
3. Rasmussen SA, Jamieson DJ, Uyeki TM. Effects of influenza on pregnant women and infants. American journal of obstetrics and gynecology. 2012 Sep;207(3 Suppl):S3-8
4. Public Health England. Immunisation against Infectious Disease (the Green Book). Chapter 24: Pertussis 2016. Available from: https://assets.publishing.service.gov.uk/government/uploads/system/uploads/attachment_data/file/514363/Pertussis_Green_Book_Chapter_24_Ap2016.pdf.
5. Thompson MG, Kwong JC, Regan AK, Katz MA, Drews SJ, Azziz-Baumgartner E, et al. Influenza Vaccine Effectiveness in Preventing Influenza-associated Hospitalizations During Pregnancy: A Multi-country Retrospective Test Negative Design Study, 2010-2016. Clinical infectious diseases : an official publication of the Infectious Diseases Society of America. 2019 Apr 24;68(9):1444-53
6. Amirthalingam G, Campbell H, Ribeiro S, Fry NK, Ramsay M, Miller E, et al. Sustained Effectiveness of the Maternal Pertussis Immunization Program in England 3 Years Following Introduction. Clinical infectious diseases : an official publication of the Infectious Diseases Society of America. 2016 Dec 1;63(suppl 4):S236-s43
7. Dabrera G, Zhao H, Andrews N, Begum F, Green H, Ellis J, et al. Effectiveness of seasonal influenza vaccination during pregnancy in preventing influenza infection in infants, England, 2013/14. Euro Surveill. 2014 Nov 13;19(45):20959
8. Public Health England. Immunisation against Infectious Disease (the Green Book). Chapter 19: Influenza. Available from: https://www.gov.uk/government/publications/influenza-the-green-book-chapter-19.
9. Wiley KE, Leask J. Respiratory vaccine uptake during pregnancy. Lancet Respir Med. 2013 Mar;1(1):9-11
10. Public Health England. Pertussis vaccination programme for pregnant women update: vaccine coverage in England, January to March 2019 and 2018/19 annual coverage. Health Protection Report [Internet]. 2019; 13. Available from: https://assets.publishing.service.gov.uk/government/uploads/system/uploads/attachment_data/file/821145/hpr2619_prntl-prtsss_VC.pdf.
11. Public Health England. Seasonal influenza vaccine uptake in GP patients: winter season 2018 to 20192019. Available from: https://assets.publishing.service.gov.uk/government/uploads/system/uploads/attachment_data/file/804889/Seasonal_influenza_vaccine_uptake_in_GP_patients_1819.pdf.
12. Yuen CY, Tarrant M. Determinants of uptake of influenza vaccination among pregnant women - a systematic review. Vaccine. 2014 Aug 6;32(36):4602-13
13. Sammon CJ, McGrogan A, Snowball J, de Vries CS. Pandemic influenza vaccination during pregnancy: an investigation of vaccine uptake during the 2009/10 pandemic

For peer review only - http://bmjopen.bmj.com/site/about/guidelines.xhtml

vaccination campaign in Great Britain. Human vaccines & immunotherapeutics. 2013 Apr;9(4):917-23
14. Byrne L, Ward C, White JM, Amirthalingam G, Edelstein M. Predictors of coverage of the national maternal pertussis and infant rotavirus vaccination programmes in England. Epidemiol Infect. 2018 Jan;146(2):197-206
15. Tessier E, Warburton F, Tsang C, Rafeeq S, Boddington N, Sinnathamby M, et al. Population-level factors predicting variation in influenza vaccine uptake among adults and young children in England, 2015/16 and 2016/17. Vaccine. 2018 May 31;36(23):3231-8
16. Carlisle N, Seed PT, Gillman L. Can common characteristics be identified as predictors for seasonal influenza vaccine uptake in pregnancy? A retrospective cohort study from a South London Hospital. Midwifery. 2019;72:67-73
17. Wilcox CR, Calvert A, Metz J, Kilich E, MacLeod R, Beadon K, et al. Determinants of Influenza and Pertussis Vaccination Uptake in Pregnancy: A Multicenter Questionnaire Study of Pregnant Women and Healthcare Professionals. The Pediatric infectious disease journal. 2019;38(6):625-30
18. McAuslane H, Utsi L, Wensley A, Coole L. Inequalities in maternal pertussis vaccination uptake: a cross-sectional survey of maternity units. Journal of public health (Oxford, England). 2018;40(1):121-8
19. Maher L, Hope K, Torvaldsen S, Lawrence G, Dawson A, Wiley K, et al. Influenza vaccination during pregnancy: coverage rates and influencing factors in two urban districts in Sydney. Vaccine. 2013 Nov 12;31(47):5557-64
20. Donaldson B, Jain P, Holder BS, Lindsey B, Regan L, Kampmann B. What determines uptake of pertussis vaccine in pregnancy? A cross sectional survey in an ethnically diverse population of pregnant women in London. Vaccine. 2015 Oct 26;33(43):5822-8
21. Herrett E, Gallagher AM, Bhaskaran K, Forbes H, Mathur R, van Staa T, et al. Data Resource Profile: Clinical Practice Research Datalink (CPRD). Int J Epidemiol. 2015 Jun;44(3):827-36
22. Williams T, van Staa T, Puri S, Eaton S. Recent advances in the utility and use of the General Practice Research Database as an example of a UK Primary Care Data resource. Ther Adv Drug Saf. 2012 Apr;3(2):89-99
23. Minassian C, Williams R, Meeraus WH, Smeeth L, Campbell OMR, Thomas SL. Methods to generate and validate a Pregnancy Register in the UK Clinical Practice Research Datalink primary care database. Pharmacoepidemiol Drug Saf. 2019 Jul;28(7):923-33
24. Mathur R, Bhaskaran K, Chaturvedi N, Leon DA, vanStaa T, Grundy E, et al. Completeness and usability of ethnicity data in UK-based primary care and hospital databases. Journal of public health (Oxford, England). 2014 Dec;36(4):684-92
25. Department for Communities and Local Government. The English Index of Multiple Deprivation 2015 – Frequently Asked Questions 2016 [23 January 2020]. Available from: https://assets.publishing.service.gov.uk/government/uploads/system/uploads/attachment_data/file/579151/English_Indices_of_Deprivation_2015_-_Frequently_Asked_Questions_Dec_2016.pdf
26. Jain A, van Hoek AJ, Walker JL, Mathur R, Smeeth L, Thomas SL. Identifying social factors amongst older individuals in linked electronic health records: An assessment in a population based study. PLoS One. 2017;12(11):e0189038
27. Bhaskaran K, Forbes HJ, Douglas I, Leon DA, Smeeth L. Representativeness and optimal use of body mass index (BMI) in the UK Clinical Practice Research Datalink (CPRD). BMJ Open. 2013 Sep 13;3(9):e003389

For peer review only - http://bmjopen.bmj.com/site/about/guidelines.xhtml

28. Walker JL, Andrews NJ, Amirthalingam G, Forbes H, Langan SM, Thomas SL. Effectiveness of herpes zoster vaccination in an older United Kingdom population. Vaccine. 2018 04 19;36(17):2371-7
29. Jain A, Walker JL, Mathur R, Forbes HJ, Langan SM, Smeeth L, et al. Zoster vaccination inequalities: A population based cohort study using linked data from the UK Clinical Practice Research Datalink. PLoS One. 2018;13(11):e0207183
30. Victora CG, Huttly SR, Fuchs SC, Olinto MT. The role of conceptual frameworks in epidemiological analysis: a hierarchical approach. Int J Epidemiol. 1997 Feb;26(1):224-7
31. Crocker-Buque T, Edelstein M, Mounier-Jack S. Interventions to reduce inequalities in vaccine uptake in children and adolescents aged <19 years: a systematic review. J Epidemiol Community Health. 2017 Jan;71(1):87-97

For peer review only

For peer review only - http://bmjopen.bmj.com/site/about/guidelines.xhtml

Table 1. Pertussis vaccine uptake by social characteristics amongst pregnant women in England, 2012 to 2015
N=62,537 from 402 practices. Overall vaccine uptake 42,099 (67.3%)

	**Total** (column %)	**Received pertussis vaccine** (unadjusted coverage) (row %)	**Minimally adjusted for year** ("minimally adjusted")	**Model 1** Additionally adjusted for IMD, region, and ethnicity	**Model 2** Additionally adjusted for maternal age	**Model 3** Additionally adjusted for number of children ("fully adjusted")
**Year**						
2012	6,717 (10.7%)	3,809 (56.7%)	1	1	1	1
2013	24,657 (39.4%)	16,749 (67.9%)	1.62 (1.53, 1.71)	1.66 (1.57, 1.75)	1.66 (1.57, 1.75)	1.69 (1.60, 1.79)
2014	20,148 (32.2%)	13,638 (67.7%)	1.60 (1.51, 1.69)	1.63 (1.54, 1.73)	1.63 (1.54, 1.73)	1.66 (1.57, 1.76)
2015	11,015 (17.6%)	7,903 (71.7%)	1.94 (1.82, 2.07)	2.00 (1.87, 2.13)	2.00 (1.87, 2.13)	2.03 (1.90, 2.17)
**Index of Multiple Deprivation (IMD) quintile**						
Least deprived	13,285 (21.2%)	10,090 (76.0%)	1	1	1	1
2	11,335 (18.1%)	8,064 (71.1%)	0.78 (0.74, 0.83)	0.79 (0.74, 0.83)	0.80 (0.75, 0.85)	0.81 (0.76, 0.86)
3	12,933 (20.7%)	8,807 (68.1%)	0.68 (0.64, 0.71)	0.68 (0.64, 0.72)	0.70 (0.66, 0.74)	0.73 (0.69, 0.77)
4	12,973 (20.7%)	8,205 (63.2%)	0.54 (0.52, 0.57)	0.56 (0.53, 0.59)	0.59 (0.56, 0.62)	0.64 (0.60, 0.67)
Most deprived	12,011 (19.2%)	6,933 (57.7%)	0.43 (0.41, 0.46)	0.45 (0.42, 0.47)	0.48 (0.45, 0.51)	0.54 (0.51, 0.57)
**Region**						
London	11,894 (19.0%)	7,239 (60.9%)	1	1	1	1
North East	1,185 (1.9%)	687 (58.0%)	0.91 (0.81, 1.03)	0.96 (0.85, 1.09)	1.00 (0.88, 1.13)	1.04 (0.92, 1.19)
North West	8,835 (14.1%)	5,873 (66.5%)	1.29 (1.22, 1.36)	1.28 (1.20, 1.35)	1.30 (1.22, 1.38)	1.36 (1.27, 1.44)
Yorkshire & The Humber	1,000 (1.6%)	699 (69.9%)	1.51 (1.31, 1.74)	1.46 (1.27, 1.69)	1.51 (1.30, 1.74)	1.54 (1.33, 1.79)
East Midlands	326 (0.5%)	243 (74.5%)	2.18 (1.69, 2.81)	2.24 (1.73, 2.90)	2.30 (1.78, 2.98)	2.38 (1.84, 3.09)
West Midlands	7,050 (11.3%)	5,046 (71.6%)	1.64 (1.54, 1.75)	1.58 (1.48, 1.69)	1.62 (1.52, 1.73)	1.72 (1.61, 1.84)
East of England	5,568 (8.9%)	4,058 (72.9%)	1.75 (1.63, 1.88)	1.50 (1.40, 1.61)	1.52 (1.41, 1.63)	1.57 (1.46, 1.69)
South West	7,002 (11.2%)	4,800 (68.6%)	1.43 (1.34, 1.52)	1.32 (1.24, 1.41)	1.35 (1.26, 1.44)	1.43 (1.33, 1.52)
South Central	10,381 (16.6%)	7,185 (69.2%)	1.45 (1.37, 1.53)	1.19 (1.12, 1.26)	1.21 (1.15, 1.29)	1.28 (1.21, 1.36)
South East Coast	9,296 (14.9%)	6,269 (67.4%)	1.33 (1.26, 1.41)	1.10 (1.04, 1.17)	1.12 (1.06, 1.19)	1.19 (1.12, 1.26)
**Ethnicity**						
White	52,598 (84.1%)	36,272 (69.0%)	1	1	1	1
South Asian	4,692 (7.5%)	2,951 (62.9%)	0.76 (0.71, 0.81)	0.83 (0.78, 0.88)	0.79 (0.74, 0.85)	0.83 (0.78, 0.88)
Black	2,583 (4.1%)	1,294 (50.1%)	0.45 (0.41, 0.48)	0.58 (0.54, 0.64)	0.56 (0.52, 0.61)	0.61 (0.56, 0.67)
Mixed	922 (1.5%)	549 (59.5%)	0.65 (0.57, 0.74)	0.72 (0.63, 0.82)	0.71 (0.62, 0.82)	0.72 (0.63, 0.83)

Other	1,742 (2.8%)	1,033 (59.3%)	0.65 (0.59, 0.72)	0.73 (0.66, 0.80)	0.70 (0.63, 0.77)	0.68 (0.62, 0.75)
**Maternal age, years**						
<20	2,079 (3.3%)	1,153 (55.5%)	0.79 (0.72, 0.87)		0.80 (0.73, 0.89)	0.81 (0.73, 0.89)
20-24	8,848 (14.1%)	5,416 (61.2%)	1		1	1
25-29	16,696 (26.7%)	11,166 (66.9%)	1.27 (1.21, 1.34)		1.24 (1.18, 1.31)	1.29 (1.22, 1.36)
30-35	20,294 (32.5%)	14,376 (70.8%)	1.54 (1.46, 1.62)		1.43 (1.35, 1.51)	1.55 (1.47, 1.64)
≥35	14,620 (23.4%)	9,988 (68.3%)	1.36 (1.29, 1.44)		1.25 (1.18, 1.32)	1.42 (1.34, 1.51)
**Number of children**						
0	26,622 (42.6%)	19,814 (74.4%)	1			1
1	22,132 (35.4%)	14,673 (66.3%)	0.67 (0.65, 0.70)			0.65 (0.63, 0.68)
2	8,645 (13.8%)	5,009 (57.9%)	0.47 (0.45, 0.49)			0.47 (0.45, 0.50)
≥3	5,138 (8.2%)	2,603 (50.7%)	0.35 (0.33, 0.37)			0.37 (0.35, 0.40)
**Body Mass Index (BMI)**						
<18.5 underweight	2,063 (3.3%)	1,265 (61.3%)	0.71 (0.64, 0.77)			
18.5-24.9	29,045 (46.4%)	20,095 (69.2%)	1			
25.0-29.9 overweight	14,211 (22.7%)	9,852 (69.3%)	1.01 (0.96, 1.05)			
≥30 obese	10,552 (16.9%)	6,833 (64.8%)	0.82 (0.78, 0.86)			
Missing	6,666 (10.7%)	4,054 (60.8%)				

Note: All models include women who registered before the end of the first trimester and delivered a live-or stillborn child on or after 26 weeks of pregnancy and exclude those with missing ethnicity; minimally adjusted model of BMI additionally excludes 6,666 women with missing BMI

For peer review only - http://bmjopen.bmj.com/site/about/guidelines.xhtml

Table 2. Influenza vaccine uptake by social characteristics amongst pregnant women in England, 2010/11 to 2015/16
N=140,141 from 456 practices. Overall vaccine uptake 54,837 (39.1%)

	**Total** (column %)	**Received influenza vaccine** (unadjusted coverage) (row %)	**Minimally adjusted for year** ("minimally adjusted")	**Model 1** Additionally adjusted for IMD, region, and ethnicity	**Model 2** Additionally adjusted for maternal age	**Model 3** Additionally adjusted for number of children	**Model 4** Additionally adjusted for clinical risk group ("fully adjusted")
**Season**							
2010	34,373 (24.5%)	11,703 (34.0%)	1	1	1	1	1
2011	32,258 (23.0%)	10,151 (31.5%)	0.89 (0.86, 0.92)	0.89 (0.86, 0.92)	0.89 (0.86, 0.92)	0.89 (0.86, 0.92)	0.89 (0.86, 0.92)
2012	26,750 (19.1%)	12,236 (45.7%)	1.63 (1.58, 1.69)	1.66 (1.61, 1.72)	1.66 (1.61, 1.72)	1.64 (1.59, 1.70)	1.65 (1.60, 1.71)
2013	21,029 (15.0%)	8,815 (41.9%)	1.40 (1.35, 1.45)	1.43 (1.38, 1.48)	1.42 (1.37, 1.47)	1.39 (1.35, 1.45)	1.40 (1.35, 1.45)
2014	15,712 (11.2%)	7,319 (46.6%)	1.69 (1.63, 1.76)	1.74 (1.67, 1.80)	1.73 (1.67, 1.80)	1.69 (1.63, 1.76)	1.70 (1.63, 1.76)
2015	10,019 (7.1%)	4,613 (46.0%)	1.65 (1.58, 1.73)	1.72 (1.65, 1.80)	1.72 (1.64, 1.80)	1.68 (1.60, 1.76)	1.68 (1.60, 1.76)
**Index of Multiple Deprivation (IMD) quintile**							
Least deprived	28,956 (20.7%)	12,744 (44.0%)	1	1	1	1	1
2	25,424 (18.1%)	10,533 (41.4%)	0.90 (0.87, 0.93)	0.91 (0.88, 0.94)	0.92 (0.89, 0.95)	0.93 (0.89, 0.96)	0.92 (0.89, 0.95)
3	29,368 (21.0%)	11,670 (39.7%)	0.84 (0.81, 0.86)	0.84 (0.82, 0.87)	0.86 (0.83, 0.89)	0.88 (0.85, 0.91)	0.88 (0.85, 0.91)
4	28,520 (20.4%)	10,278 (36.0%)	0.71 (0.69, 0.74)	0.72 (0.69, 0.74)	0.74 (0.71, 0.77)	0.77 (0.74, 0.79)	0.76 (0.74, 0.79)
Most deprived	27,873 (19.9%)	9,612 (34.5%)	0.67 (0.65, 0.70)	0.66 (0.64, 0.68)	0.69 (0.66, 0.71)	0.73 (0.70, 0.76)	0.72 (0.70, 0.75)
**Region**							
London	26,171 (18.7%)	9,146 (34.9%)	1	1	1	1	1
North East	2,758 (2.0%)	989 (35.9%)	1.11 (1.02, 1.21)	1.16 (1.07, 1.27)	1.19 (1.09, 1.29)	1.21 (1.11, 1.31)	1.21 (1.11, 1.31)
North West	19,060 (13.6%)	7,870 (41.3%)	1.37 (1.32, 1.42)	1.39 (1.33, 1.45)	1.40 (1.35, 1.46)	1.43 (1.37, 1.49)	1.42 (1.36, 1.47)
Yorkshire & The Humber	2,840 (2.0%)	1,090 (38.4%)	1.27 (1.18, 1.38)	1.24 (1.15, 1.35)	1.26 (1.16, 1.37)	1.26 (1.16, 1.37)	1.26 (1.16, 1.37)
East Midlands	1,940 (1.4%)	717 (37.0%)	1.33 (1.21, 1.47)	1.37 (1.24, 1.51)	1.39 (1.26, 1.53)	1.41 (1.27, 1.55)	1.40 (1.27, 1.55)
West Midlands	15,846 (11.3%)	6,692 (42.2%)	1.41 (1.35, 1.46)	1.40 (1.34, 1.46)	1.41 (1.36, 1.47)	1.44 (1.38, 1.51)	1.43 (1.37, 1.49)
East of England	13,695 (9.8%)	5,468 (39.9%)	1.31 (1.26, 1.37)	1.23 (1.18, 1.29)	1.24 (1.19, 1.29)	1.25 (1.20, 1.31)	1.24 (1.19, 1.30)
South West	16,546 (11.8%)	6,504 (39.3%)	1.25 (1.20, 1.31)	1.22 (1.17, 1.28)	1.24 (1.19, 1.29)	1.27 (1.21, 1.32)	1.25 (1.20, 1.31)
South Central	21,435 (15.3%)	9,125 (42.6%)	1.42 (1.36, 1.47)	1.30 (1.25, 1.35)	1.31 (1.26, 1.36)	1.34 (1.29, 1.39)	1.33 (1.28, 1.38)
South East Coast	19,850 (14.2%)	7,236 (36.5%)	1.06 (1.02, 1.10)	0.99 (0.95, 1.03)	1.00 (0.96, 1.04)	1.02 (0.98, 1.06)	1.02 (0.98, 1.06)
**Ethnicity**							
White	117,469 (83.8%)	46,781 (39.8%)	1	1	1	1	1
South Asian	10,827 (7.7%)	4,103 (37.9%)	0.92 (0.88, 0.95)	0.98 (0.94, 1.02)	0.96 (0.92, 1.00)	0.98 (0.94, 1.02)	0.99 (0.95, 1.03)
Black	5,853 (4.2%)	1,837 (31.4%)	0.67 (0.64, 0.71)	0.81 (0.76, 0.86)	0.80 (0.75, 0.85)	0.83 (0.78, 0.88)	0.83 (0.78, 0.88)
Mixed	2,094 (1.5%)	757 (36.2%)	0.84 (0.77, 0.92)	0.90 (0.82, 0.99)	0.90 (0.82, 0.99)	0.91 (0.83, 0.99)	0.91 (0.83, 0.99)
Other	3,898 (2.8%)	1,359 (34.9%)	0.79 (0.73, 0.84)	0.85 (0.80, 0.91)	0.84 (0.78, 0.90)	0.83 (0.78, 0.89)	0.85 (0.79, 0.91)

For peer review only - http://bmjopen.bmj.com/site/about/guidelines.xhtml

**Maternal age, years**							
<20	5,536 (4.0%)	1,817 (32.8%)	0.87 (0.81, 0.92)		0.87 (0.82, 0.93)	0.87 (0.82, 0.93)	0.87 (0.82, 0.93)
20-24	21,663 (15.5%)	7,797 (36.0%)	1		1	1	1
25-29	37,985 (27.1%)	14,827 (39.0%)	1.13 (1.09, 1.17)		1.11 (1.07, 1.15)	1.12 (1.09, 1.16)	1.12 (1.08, 1.16)
30-35	43,777 (31.2%)	17,950 (41.0%)	1.22 (1.18, 1.26)		1.18 (1.14, 1.22)	1.21 (1.17, 1.26)	1.21 (1.17, 1.25)
≥35	31,180 (22.2%)	12,446 (39.9%)	1.17 (1.12, 1.21)		1.12 (1.08, 1.16)	1.19 (1.15, 1.24)	1.18 (1.13, 1.22)
**Number of children**							
0	66,112 (47.2%)	28,457 (43.0%)	1			1	1
1	45,969 (32.8%)	17,092 (37.2%)	0.80 (0.78, 0.82)			0.80 (0.78, 0.82)	0.80 (0.78, 0.82)
2	18,192 (13.0%)	6,242 (34.3%)	0.71 (0.68, 0.73)			0.72 (0.69, 0.74)	0.71 (0.69, 0.74)
≥3	9,868 (7.0%)	3,046 (30.9%)	0.61 (0.58, 0.63)			0.63 (0.60, 0.66)	0.62 (0.59, 0.65)
**Clinical risk group recommended for influenza vaccination**							
No	130,160 (92.9%)	49,752 (38.2%)	1				1
Yes	9,981 (7.1%)	5,085 (50.9%)	1.69 (1.62, 1.76)				1.70 (1.63, 1.77)
**Body Mass Index (BMI)**							
<18.5 Underweight	4,865 (3.5%)	1,744 (35.8%)	0.85 (0.80, 0.90)				
18.5-24.9	66,405 (47.4%)	26,331 (39.7%)	1				
25.0-29.9 Overweight	31,855 (22.7%)	12,882 (40.4%)	1.04 (1.01, 1.07)				
≥30 Obese	23,142 (16.5%)	9,222 (39.8%)	1.00 (0.97, 1.03)				
Missing	13,874 (9.9%)	4,658 (33.6%)					

Note: All models include women who registered before the end of the first trimester, and exclude those with no recorded pregnancy outcome or missing ethnicity; minimally adjusted model of BMI additionally excludes 13,874 women with missing BMI

For peer review only - http://bmjopen.bmj.com/site/about/guidelines.xhtml

Supplementary material

Social determinants of pertussis and influenza vaccine uptake in pregnancy: a national cohort study using electronic health records

Authors: Jemma L Walker,* Christopher T Rentsch,* Helen I McDonald, Jeongeun Bak, Caroline Minassian, Gayatri Amirthalingam, Michael Edelstein, Sara L Thomas.

Supplementary Table 1: Hierarchical conceptual framework and interpretation of effect estimates

Supplementary Table 2: Patterns of social factors amongst pregnant women with and without a recorded ethnicity status, 2010-2015

Supplementary Table 3: ‘Pertussis BMI Model’ complete case analysis additionally excluding 6,666 women with missing BMI for pertussis vaccine uptake amongst pregnant women in the UK, 2012-2015

Supplementary Table 4: ‘Influenza BMI Model’ complete case analysis additionally excluding 13,874 women with missing BMI for influenza vaccine uptake amongst pregnant women in the UK, 2010-2015

Supplementary Table 5: Sensitivity analyses expanding definition of inclusion criteria for the pertussis vaccine uptake models: registration by end of pregnancy and ImmForm approach compared to primary analyses

Supplementary Table 7: Secondary analysis of subsequent pertussis vaccine uptake among women who had received pertussis vaccination in their first eligible pregnancy and had a second eligible pregnancy within the study period (N=3,111)

For peer review only - http://bmjopen.bmj.com/site/about/guidelines.xhtml

Supplementary Table 1: Hierarchical conceptual framework and interpretation of effect estimates

This table is reproduced from Supplementary Table 6 in Jain A., Walker JL, Forbes H, Langan S, Smeeth L, van Hoek AJ and Thomas SL. Zoster vaccination inequalities: A population based cohort study using linked data from the UK Clinical Practice Research Datalink. PLoS One 2018;13(11):e0207183. doi: 10.1371/journal.pone.0207183.

(based on [1])

Hierarchical models	Explanatory variables	Interpretation of effect estimates
`Minimally' adjusted model	Each explanatory variable adjusted in-turn for *a priori* confounders: year of birth and gender	Effect estimate of each variable adjusted for *a priori* confounders.
Model-1*^	Ethnicity +immigration status^ with *a priori* confounders	Effects of ethnicity and immigration status adjusted for each other and *a priori* confounders
Model-2*	Model-1+ patient-LSOA-level deprivation#	(i) Effects of ethnicity and immigration status not mediated via deprivation and adjusted for each other and *a priori* confounders<br>(ii) Effect of patient-LSOA-level deprivation adjusted for *a priori* confounders, ethnicity and immigration status
Model-3*	Model-2 + rest of the explanatory variables~	(i) Effect of ethnicity and immigration status not mediated via deprivation and other explanatory variables~ *<br>(ii) Effect of deprivation not mediated via other explanatory variables~*<br>(iii) Effect of other explanatory variables~ *

*all variables in the model adjusted for each other and *a priori* confounders: year of birth, sex and calendar period ^ethnicity and immigration status examined for multicollinearity LSOA Lower-layer Super Output Area # patient-LSOA-level and practice-LSOA-level deprivation were considered to be correlated therefore only patient-LSOA-level deprivation used ~ care home residence, living alone status and cohabitation status (living alone and cohabitation examined for multicollinearity)

1. Victora CG, Huttly SR, Fuchs SC, Olinto MT. The role of conceptual frameworks in epidemiological analysis: a hierarchical approach. Int J Epidemiol. 1997;26(1):224-7. PubMed PMID: 9126524.

For peer review only - http://bmjopen.bmj.com/site/about/guidelines.xhtml

Supplementary Table 2: Patterns of social factors amongst pregnant women with and without a recorded ethnicity status, 2010-2015

		Pertussis		Influenza	
		Recorded ethnicity	Missing ethnicity	Recorded ethnicity	Missing ethnicity
		n=62,537	n=5,553	n=140,141	n=11,991
Year/season	2010	-	-	34,373 (24.5%)	2,433 (20.3%)
	2011	-	-	32,258 (23.0%)	2,228 (18.6%)
	2012	6,717 (10.7%)	506 (9.1%)	26,750 (19.1%)	1,791 (14.9%)
	2013	24,657 (39.4%)	1,789 (32.2%)	21,029 (15.0%)	1,730 (14.4%)
	2014	20,148 (32.2%)	1,910 (34.4%)	15,712 (11.2%)	1,882 (15.7%)
	2015	11,015 (17.6%)	1,348 (24.3%)	10,019 (7.1%)	1,927 (16.1%)
Index of multiple deprivation (IMD) quintile	Least deprived	13,285 (21.2%)	1,522 (27.4%)	28,956 (20.7%)	3,203 (26.7%)
	2	11,335 (18.1%)	883 (15.9%)	25,424 (18.1%)	1,896 (15.8%)
	3	12,933 (20.7%)	992 (17.9%)	29,368 (21.0%)	2,245 (18.7%)
	4	12,973 (20.7%)	1,592 (28.7%)	28,520 (20.4%)	3,265 (27.2%)
	Most deprived	12,011 (19.2%)	564 (10.2%)	27,873 (19.9%)	1,382 (11.5%)
Region	London	11,894 (19.0%)	502 (9.0%)	26,171 (18.7%)	1,144 (9.5%)
	North East	1,185 (1.9%)	60 (1.1%)	2,758 (2.0%)	173 (1.4%)
	North West	8,835 (14.1%)	917 (16.5%)	19,060 (13.6%)	1,761 (14.7%)
	Yorkshire & The Humber	1,000 (1.6%)	5 (0.1%)	2,840 (2.0%)	24 (0.2%)
	East Midlands	326 (0.5%)	70 (1.3%)	1,940 (1.4%)	435 (3.6%)
	West Midlands	7,050 (11.3%)	530 (9.5%)	15,846 (11.3%)	1,231 (10.3%)
	East of England	5,568 (8.9%)	464 (8.4%)	13,695 (9.8%)	1,025 (8.5%)
	South West	7,002 (11.2%)	223 (4.0%)	16,546 (11.8%)	574 (4.8%)
	South Central	10,381 (16.6%)	1,692 (30.5%)	21,435 (15.3%)	3,215 (26.8%)
	South East Coast	9,296 (14.9%)	1,090 (19.6%)	19,850 (14.2%)	2,409 (20.1%)
Ethnicity	White	52,598 (84.1%)	-	117,469 (83.8%)	-
	South Asian	4,692 (7.5%)	-	10,827 (7.7%)	-
	Black	2,583 (4.1%)	-	5,853 (4.2%)	-
	Mixed	922 (1.5%)	-	2,094 (1.5%)	-
	Other	1,742 (2.8%)	-	3,898 (2.8%)	-
Maternal age, years	<20	2,079 (3.3%)	218 (3.9%)	5,536 (4.0%)	583 (4.9%)
	20-24	8,848 (14.1%)	914 (16.5%)	21,663 (15.5%)	2,014 (16.8%)
	25-29	16,696 (26.7%)	1,391 (25.0%)	37,985 (27.1%)	3,004 (25.1%)
	30-35	20,294 (32.5%)	1,673 (30.1%)	43,777 (31.2%)	3,639 (30.3%)
	≥35	14,620 (23.4%)	1,357 (24.4%)	31,180 (22.2%)	2,751 (22.9%)
Number of children	0	26,622 (42.6%)	2,645 (47.6%)	66,112 (47.2%)	6,255 (52.2%)
	1	22,132 (35.4%)	1,675 (30.2%)	45,969 (32.8%)	3,312 (27.6%)
	2	8,645 (13.8%)	679 (12.2%)	18,192 (13.0%)	1,431 (11.9%)
	≥3	5,138 (8.2%)	554 (10.0%)	9,868 (7.0%)	993 (8.3%)
Clinical risk group	No	-	-	130,160 (92.9%)	11,238 (93.7%)
	Yes	-	-	9,981 (7.1%)	753 (6.3%)
Body mass index (BMI)	<18.5	2,063 (3.3%)	201 (3.6%)	4,865 (3.5%)	434 (3.6%)
	18.5-24.9	29,045 (46.4%)	2,489 (44.8%)	66,405 (47.4%)	5,571 (46.5%)
	25.0-29.9	14,211 (22.7%)	1,203 (21.7%)	31,855 (22.7%)	2,563 (21.4%)
	≥30	10,552 (16.9%)	785 (14.1%)	23,142 (16.5%)	1,747 (14.6%)
	Missing	6,666 (10.7%)	875 (15.8%)	13,874 (9.9%)	1,676 (14.0%)
Note: all p<0.001					

For peer review only

Supplementary Table 3: 'Pertussis BMI Model' complete case analysis additionally excluding 6,666 women with missing BMI for pertussis vaccine uptake amongst pregnant women in the UK, 2012-2015

		**Minimally adjusted** for year	**Model 3 (fully adjusted in main analysis)** Adjusted for year, IMD, region, ethnicity, maternal age and number of children	**BMI Model** As Model 3 and additionally adjusted for BMI
N		55,871	55,871	55,871
Year	2012	1	1	1
	2013	1.65 (1.56, 1.75)	1.74 (1.63, 1.84)	1.74 (1.63, 1.84)
	2014	1.63 (1.54, 1.73)	1.70 (1.60, 1.81)	1.70 (1.60, 1.81)
	2015	1.95 (1.82, 2.08)	2.04 (1.90, 2.19)	2.04 (1.91, 2.19)
Index of multiple deprivation (IMD) quintile	Least deprived	1	1	1
	2	0.78 (0.73, 0.83)	0.81 (0.76, 0.86)	0.81 (0.76, 0.86)
	3	0.67 (0.64, 0.71)	0.72 (0.68, 0.77)	0.72 (0.68, 0.77)
	4	0.54 (0.51, 0.58)	0.63 (0.59, 0.67)	0.63 (0.59, 0.67)
	Most deprived	0.44 (0.41, 0.46)	0.53 (0.50, 0.57)	0.54 (0.50, 0.57)
Region	London	1	1	1
	North East	0.96 (0.84, 1.10)	1.12 (0.98, 1.29)	1.12 (0.97, 1.28)
	North West	1.30 (1.22, 1.38)	1.36 (1.28, 1.46)	1.36 (1.28, 1.46)
	Yorkshire & The Humber	1.49 (1.29, 1.72)	1.51 (1.30, 1.76)	1.51 (1.30, 1.76)
	East Midlands	1.87 (1.44, 2.42)	2.33 (1.78, 3.04)	2.31 (1.77, 3.02)
	West Midlands	1.60 (1.50, 1.71)	1.70 (1.59, 1.83)	1.70 (1.58, 1.82)
	East of England	1.73 (1.60, 1.86)	1.56 (1.44, 1.68)	1.56 (1.44, 1.68)
	South West	1.41 (1.32, 1.51)	1.42 (1.33, 1.53)	1.42 (1.33, 1.53)
	South Central	1.46 (1.37, 1.55)	1.29 (1.21, 1.37)	1.29 (1.21, 1.37)
	South East Coast	1.33 (1.26, 1.42)	1.18 (1.11, 1.26)	1.18 (1.11, 1.26)
Ethnicity	White	1	1	1
	South Asian	0.74 (0.70, 0.79)	0.83 (0.77, 0.89)	0.83 (0.77, 0.89)
	Black	0.45 (0.41, 0.49)	0.62 (0.57, 0.68)	0.62 (0.56, 0.67)
	Mixed	0.69 (0.60, 0.79)	0.75 (0.65, 0.87)	0.75 (0.65, 0.87)
	Other	0.63 (0.57, 0.70)	0.67 (0.60, 0.75)	0.68 (0.61, 0.75)
Maternal age, years	<20	0.85 (0.75, 0.96)	0.84 (0.74, 0.96)	0.85 (0.75, 0.97)
	20-24	1	1	1
	25-29	1.27 (1.20, 1.34)	1.28 (1.20, 1.36)	1.27 (1.20, 1.35)
	30-35	1.49 (1.41, 1.58)	1.51 (1.42, 1.60)	1.49 (1.41, 1.58)
	≥35	1.32 (1.24, 1.40)	1.37 (1.29, 1.46)	1.36 (1.28, 1.45)
Number of children	0	1	1	1
	1	0.67 (0.65, 0.70)	0.66 (0.63, 0.68)	0.66 (0.63, 0.68)
	2	0.47 (0.44, 0.49)	0.47 (0.45, 0.50)	0.47 (0.45, 0.50)
	≥3	0.35 (0.33, 0.38)	0.37 (0.35, 0.40)	0.37 (0.35, 0.40)
Body mass index (BMI)	<18.5	0.71 (0.64, 0.77)		0.77 (0.70, 0.85)
	18.5-24.9	1		1
	25.0-29.9	1.01 (0.96, 1.05)		1.10 (1.05, 1.15)
	≥30	0.82 (0.78, 0.86)		0.96 (0.91, 1.00)
Note: Model inclusion as per the main analysis but additionally excluding 6,666 women with missing BMI				

For peer review only - http://bmjopen.bmj.com/site/about/guidelines.xhtml

Supplementary Table 4: 'Influenza BMI Model' complete case analysis additionally excluding 13,874 women with missing BMI for influenza vaccine uptake amongst pregnant women in the UK, 2010-2015

		**Minimally adjusted** for year	**Model 4** adjusted for year, IMD,region, ethnicity, maternal age, number of children and clinical risk group	**BMI Model** as Model 4 and additionally adjusted for BMI
N		126,267	126,267	126,267
Year	2010	1	1	1
	2011	0.90 (0.87, 0.93)	0.90 (0.87, 0.93)	0.90 (0.87, 0.93)
	2012	1.63 (1.57, 1.68)	1.64 (1.59, 1.70)	1.64 (1.59, 1.70)
	2013	1.41 (1.36, 1.46)	1.41 (1.35, 1.46)	1.40 (1.35, 1.46)
	2014	1.70 (1.63, 1.77)	1.70 (1.63, 1.77)	1.70 (1.63, 1.77)
	2015	1.66 (1.58, 1.74)	1.67 (1.60, 1.76)	1.67 (1.59, 1.76)
Index of multiple deprivation (IMD) quintile	Least deprived	1	1	1
	2	0.90 (0.87, 0.94)	0.92 (0.89, 0.95)	0.92 (0.88, 0.95)
	3	0.83 (0.80, 0.86)	0.87 (0.84, 0.90)	0.86 (0.83, 0.90)
	4	0.71 (0.69, 0.74)	0.76 (0.73, 0.79)	0.75 (0.73, 0.78)
	Most deprived	0.68 (0.65, 0.70)	0.72 (0.69, 0.75)	0.71 (0.69, 0.74)
Region	London	1	1	1
	North East	1.17 (1.07, 1.28)	1.26 (1.15, 1.37)	1.25 (1.14, 1.37)
	North West	1.40 (1.34, 1.45)	1.43 (1.37, 1.49)	1.43 (1.37, 1.49)
	Yorkshire & The Humber	1.28 (1.18, 1.39)	1.26 (1.16, 1.37)	1.25 (1.15, 1.36)
	East Midlands	1.29 (1.17, 1.43)	1.35 (1.22, 1.49)	1.34 (1.21, 1.49)
	West Midlands	1.41 (1.35, 1.47)	1.44 (1.37, 1.50)	1.43 (1.37, 1.49)
	East of England	1.30 (1.24, 1.36)	1.23 (1.17, 1.28)	1.22 (1.17, 1.28)
	South West	1.29 (1.23, 1.34)	1.28 (1.22, 1.34)	1.27 (1.22, 1.33)
	South Central	1.45 (1.39, 1.51)	1.35 (1.30, 1.41)	1.35 (1.29, 1.40)
	South East Coast	1.08 (1.04, 1.12)	1.03 (0.99, 1.07)	1.03 (0.99, 1.07)
Ethnicity	White	1	1	1
	South Asian	0.90 (0.87, 0.94)	0.99 (0.95, 1.03)	0.99 (0.95, 1.04)
	Black	0.66 (0.62, 0.70)	0.83 (0.78, 0.88)	0.82 (0.77, 0.87)
	Mixed	0.85 (0.78, 0.94)	0.92 (0.84, 1.01)	0.92 (0.84, 1.01)
	Other	0.78 (0.73, 0.84)	0.86 (0.80, 0.92)	0.87 (0.80, 0.93)
Maternal age, years	<20	0.90 (0.84, 0.98)	0.90 (0.83, 0.97)	0.91 (0.84, 0.98)
	20-24	1	1	1
	25-29	1.12 (1.08, 1.16)	1.11 (1.07, 1.15)	1.11 (1.07, 1.15)
	30-35	1.20 (1.16, 1.24)	1.19 (1.15, 1.23)	1.19 (1.14, 1.23)
	≥35	1.14 (1.10, 1.18)	1.15 (1.11, 1.20)	1.15 (1.10, 1.19)
Number of children	0	1	1	1
	1	0.80 (0.78, 0.82)	0.79 (0.77, 0.81)	0.79 (0.77, 0.81)
	2	0.70 (0.68, 0.73)	0.71 (0.68, 0.73)	0.70 (0.68, 0.73)
	≥3	0.61 (0.58, 0.64)	0.62 (0.59, 0.66)	0.62 (0.59, 0.65)
Clinical risk group	No	1	1	1
	Yes	1.69 (1.62, 1.76)	1.69 (1.62, 1.77)	1.68 (1.61, 1.76)
Body mass index (BMI)	<18.5	0.85 (0.80, 0.90)		0.89 (0.84, 0.95)
	18.5-24.9	1		1
	25.0-29.9	1.04 (1.01, 1.07)		1.07 (1.04, 1.10)
	≥30	1.00 (0.97, 1.03)		1.06 (1.03, 1.09)
Note: Model inclusion as per the main analysis but additionally excluding 13,874 women with missing BMI				

For peer review only - http://bmjopen.bmj.com/site/about/guidelines.xhtml

Supplementary Table 5: Sensitivity analyses expanding definition of inclusion criteria for the pertussis vaccine uptake models: registration by end of pregnancy and ImmForm approach compared to primary analyses

		Primary analyses		Registered by end of pregnancy		ImmForm approach	
		Minimally adjusted	Fully adjusted	Minimally adjusted	Fully adjusted	Minimally adjusted	Fully adjusted
N		62,537	62,537	80,831	80,831	90,720	90,720
Year	2012	1	1	1	1	1	1
	2013	1.62 (1.53, 1.71)	1.69 (1.60, 1.79)	1.59 (1.52, 1.67)	1.65 (1.58, 1.73)	1.55 (1.48, 1.62)	1.60 (1.53, 1.67)
	2014	1.60 (1.51, 1.69)	1.66 (1.57, 1.76)	1.69 (1.61, 1.77)	1.72 (1.64, 1.81)	1.64 (1.57, 1.72)	1.67 (1.60, 1.75)
	2015	1.94 (1.82, 2.07)	2.03 (1.90, 2.17)	2.09 (1.98, 2.21)	2.13 (2.02, 2.26)	2.04 (1.94, 2.15)	2.07 (1.96, 2.19)
Index of multiple deprivation (IMD) quintile	Least deprived	1	1	1	1	1	1
	2	0.78 (0.74, 0.83)	0.81 (0.76, 0.86)	0.79 (0.76, 0.83)	0.83 (0.79, 0.87)	0.79 (0.76, 0.83)	0.83 (0.79, 0.87)
	3	0.68 (0.64, 0.71)	0.73 (0.69, 0.77)	0.71 (0.68, 0.74)	0.78 (0.74, 0.81)	0.70 (0.67, 0.73)	0.76 (0.73, 0.80)
	4	0.54 (0.52, 0.57)	0.64 (0.60, 0.67)	0.58 (0.56, 0.61)	0.69 (0.66, 0.73)	0.58 (0.56, 0.61)	0.69 (0.66, 0.72)
	Most deprived	0.43 (0.41, 0.46)	0.54 (0.51, 0.57)	0.46 (0.44, 0.49)	0.59 (0.56, 0.62)	0.46 (0.44, 0.48)	0.58 (0.55, 0.60)
Region	London	1	1	1	1	1	1
	North East	0.91 (0.81, 1.03)	1.04 (0.92, 1.19)	1.01 (0.90, 1.13)	1.17 (1.05, 1.32)	1.03 (0.93, 1.15)	1.21 (1.08, 1.35)
	North West	1.29 (1.22, 1.36)	1.36 (1.27, 1.44)	1.31 (1.25, 1.38)	1.41 (1.34, 1.49)	1.30 (1.24, 1.36)	1.40 (1.34, 1.48)
	Yorkshire & The Humber	1.51 (1.31, 1.74)	1.54 (1.33, 1.79)	1.48 (1.31, 1.68)	1.55 (1.37, 1.76)	1.44 (1.28, 1.62)	1.53 (1.35, 1.73)
	East Midlands	2.18 (1.69, 2.81)	2.38 (1.84, 3.09)	2.12 (1.70, 2.65)	2.36 (1.88, 2.96)	2.16 (1.75, 2.67)	2.43 (1.96, 3.02)
	West Midlands	1.64 (1.54, 1.75)	1.72 (1.61, 1.84)	1.61 (1.53, 1.70)	1.73 (1.63, 1.83)	1.55 (1.47, 1.63)	1.67 (1.58, 1.76)
	East of England	1.75 (1.63, 1.88)	1.57 (1.46, 1.69)	1.65 (1.55, 1.75)	1.49 (1.40, 1.58)	1.65 (1.56, 1.74)	1.49 (1.41, 1.58)
	South West	1.43 (1.34, 1.52)	1.43 (1.33, 1.52)	1.48 (1.41, 1.56)	1.49 (1.41, 1.57)	1.49 (1.42, 1.57)	1.51 (1.43, 1.59)
	South Central	1.45 (1.37, 1.53)	1.28 (1.21, 1.36)	1.54 (1.47, 1.62)	1.41 (1.34, 1.48)	1.51 (1.44, 1.58)	1.38 (1.32, 1.45)
	South East Coast	1.33 (1.26, 1.41)	1.19 (1.12, 1.26)	1.33 (1.26, 1.39)	1.24 (1.17, 1.30)	1.30 (1.24, 1.36)	1.21 (1.15, 1.27)
Ethnicity	White	1	1	1	1	1	1
	South Asian	0.76 (0.71, 0.81)	0.83 (0.78, 0.88)	0.78 (0.74, 0.83)	0.84 (0.79, 0.88)	0.78 (0.75, 0.82)	0.84 (0.80, 0.89)
	Black	0.45 (0.41, 0.48)	0.61 (0.56, 0.67)	0.46 (0.43, 0.50)	0.61 (0.57, 0.66)	0.47 (0.44, 0.51)	0.63 (0.59, 0.67)
	Mixed	0.65 (0.57, 0.74)	0.72 (0.63, 0.83)	0.64 (0.57, 0.71)	0.70 (0.62, 0.79)	0.63 (0.57, 0.70)	0.69 (0.62, 0.77)
	Other	0.65 (0.59, 0.72)	0.68 (0.62, 0.75)	0.66 (0.61, 0.71)	0.69 (0.63, 0.74)	0.65 (0.61, 0.70)	0.68 (0.63, 0.74)
Maternal age, years	<20	0.79 (0.72, 0.87)	0.81 (0.73, 0.89)	0.73 (0.67, 0.79)	0.73 (0.68, 0.79)	0.74 (0.68, 0.79)	0.74 (0.69, 0.80)
	20-24	1	1	1	1	1	1
	25-29	1.27 (1.21, 1.34)	1.29 (1.22, 1.36)	1.28 (1.23, 1.34)	1.30 (1.25, 1.37)	1.26 (1.21, 1.32)	1.28 (1.23, 1.34)
	30-35	1.54 (1.46, 1.62)	1.55 (1.47, 1.64)	1.55 (1.49, 1.62)	1.57 (1.50, 1.65)	1.54 (1.47, 1.60)	1.55 (1.48, 1.62)
	≥35	1.36 (1.29, 1.44)	1.42 (1.34, 1.51)	1.41 (1.35, 1.48)	1.48 (1.41, 1.55)	1.38 (1.32, 1.44)	1.44 (1.37, 1.51)
Number of children	0	1	1	1	1	1	1
	1	0.67 (0.65, 0.70)	0.65 (0.63, 0.68)	0.69 (0.66, 0.71)	0.67 (0.65, 0.69)	0.70 (0.67, 0.72)	0.68 (0.66, 0.70)
	2	0.47 (0.45, 0.49)	0.47 (0.45, 0.50)	0.50 (0.48, 0.52)	0.49 (0.47, 0.51)	0.50 (0.48, 0.52)	0.50 (0.48, 0.52)
	≥3	0.35 (0.33, 0.37)	0.37 (0.35, 0.40)	0.38 (0.36, 0.40)	0.39 (0.37, 0.42)	0.39 (0.37, 0.41)	0.40 (0.38, 0.42)
Body mass index (BMI)	<18.5	0.71 (0.64, 0.77)		0.69 (0.64, 0.75)		0.71 (0.66, 0.77)	
	18.5-24.9	1		1		1	
	25.0-29.9	1.01 (0.96, 1.05)		0.97 (0.94, 1.01)		0.98 (0.94, 1.01)	
	≥30	0.82 (0.78, 0.86)		0.82 (0.79, 0.85)		0.82 (0.79, 0.85)	
Note: All models include women who registered in first trimester and exclude those with missing ethnicity; minimally adjusted models of BMI excludes women with missing BMI							
Abbreviations: UK, United Kingdom; IMD, Index of Multiple Deprivation; BMI, body mass index							

For peer review only - http://bmjopen.bmj.com/site/about/guidelines.xhtml

Supplementary Table 6: Sensitivity analyses expanding definition of inclusion criteria for the influenza vaccine uptake models: registration by end of pregnancy, including pregnancies without known outcomes, extending influenza season to March, compared to primary analyses

		Primary analyses		Registered by end of pregnancy		Including pregnancies without known outcomes		Extending influenza season through March	
		Minimally adjusted	Fully adjusted	Minimally adjusted	Fully adjusted	Minimally adjusted	Fully adjusted	Minimally adjusted	Fully adjusted
N		140,141	140,141	153,782	153,782	191,950	191,950	140,141	140,141
Season	2010	1	1	1	1	1	1	1	1
	2011	0.89 (0.86, 0.92)	0.89 (0.86, 0.92)	0.89 (0.86, 0.92)	0.89 (0.86, 0.92)	0.92 (0.89, 0.95)	0.92 (0.89, 0.94)	0.93 (0.90, 0.96)	0.93 (0.90, 0.96)
	2012	1.63 (1.58, 1.69)	1.65 (1.60, 1.71)	1.62 (1.57, 1.67)	1.63 (1.58, 1.68)	1.55 (1.51, 1.60)	1.56 (1.52, 1.61)	1.81 (1.76, 1.87)	1.84 (1.78, 1.90)
	2013	1.40 (1.35, 1.45)	1.40 (1.35, 1.45)	1.41 (1.36, 1.45)	1.41 (1.36, 1.46)	1.36 (1.32, 1.41)	1.36 (1.32, 1.41)	1.57 (1.51, 1.62)	1.57 (1.51, 1.62)
	2014	1.69 (1.63, 1.76)	1.70 (1.63, 1.76)	1.71 (1.65, 1.77)	1.72 (1.65, 1.78)	1.64 (1.59, 1.70)	1.63 (1.58, 1.69)	1.88 (1.81, 1.95)	1.89 (1.82, 1.96)
	2015	1.65 (1.58, 1.73)	1.68 (1.60, 1.76)	1.67 (1.60, 1.75)	1.70 (1.63, 1.78)	1.61 (1.54, 1.68)	1.61 (1.55, 1.68)	1.86 (1.78, 1.94)	1.89 (1.81, 1.98)
IMD	Least deprived	1	1	1	1	1	1	1	1
	2	0.90 (0.87, 0.93)	0.92 (0.89, 0.95)	0.88 (0.86, 0.91)	0.90 (0.87, 0.94)	0.87 (0.84, 0.90)	0.89 (0.86, 0.92)	0.90 (0.87, 0.93)	0.91 (0.88, 0.95)
	3	0.84 (0.81, 0.86)	0.88 (0.85, 0.91)	0.83 (0.81, 0.86)	0.87 (0.84, 0.90)	0.83 (0.80, 0.85)	0.87 (0.85, 0.90)	0.83 (0.81, 0.86)	0.87 (0.84, 0.90)
	4	0.71 (0.69, 0.74)	0.76 (0.74, 0.79)	0.71 (0.69, 0.73)	0.76 (0.74, 0.79)	0.71 (0.69, 0.74)	0.78 (0.75, 0.80)	0.70 (0.68, 0.73)	0.75 (0.73, 0.78)
	Most deprived	0.67 (0.65, 0.70)	0.72 (0.70, 0.75)	0.67 (0.65, 0.69)	0.72 (0.69, 0.74)	0.69 (0.67, 0.71)	0.76 (0.73, 0.78)	0.67 (0.65, 0.69)	0.71 (0.69, 0.74)
Region	London	1	1	1	1	1	1	1	1
	North East	1.11 (1.02, 1.21)	1.21 (1.11, 1.31)	1.14 (1.06, 1.24)	1.24 (1.14, 1.34)	1.15 (1.07, 1.24)	1.25 (1.16, 1.35)	1.09 (1.01, 1.18)	1.19 (1.09, 1.29)
	North West	1.37 (1.32, 1.42)	1.42 (1.36, 1.47)	1.39 (1.34, 1.45)	1.44 (1.39, 1.50)	1.42 (1.38, 1.47)	1.48 (1.42, 1.53)	1.38 (1.33, 1.44)	1.44 (1.38, 1.50)
	Yorkshire & The Humber	1.27 (1.18, 1.38)	1.26 (1.16, 1.37)	1.32 (1.22, 1.43)	1.31 (1.21, 1.42)	1.33 (1.24, 1.43)	1.33 (1.23, 1.43)	1.27 (1.17, 1.38)	1.26 (1.17, 1.37)
	East Midlands	1.33 (1.21, 1.47)	1.40 (1.27, 1.55)	1.34 (1.22, 1.47)	1.41 (1.29, 1.55)	1.33 (1.23, 1.45)	1.39 (1.28, 1.52)	1.35 (1.23, 1.49)	1.43 (1.30, 1.58)
	West Midlands	1.41 (1.35, 1.46)	1.43 (1.37, 1.49)	1.42 (1.37, 1.48)	1.45 (1.39, 1.51)	1.43 (1.38, 1.48)	1.45 (1.40, 1.51)	1.47 (1.41, 1.53)	1.50 (1.44, 1.57)
	East of England	1.31 (1.26, 1.37)	1.24 (1.19, 1.30)	1.31 (1.26, 1.37)	1.24 (1.19, 1.30)	1.31 (1.26, 1.36)	1.24 (1.19, 1.29)	1.32 (1.26, 1.37)	1.25 (1.20, 1.31)
	South West	1.25 (1.20, 1.31)	1.25 (1.20, 1.31)	1.29 (1.24, 1.35)	1.29 (1.24, 1.35)	1.34 (1.29, 1.39)	1.34 (1.29, 1.39)	1.27 (1.22, 1.32)	1.27 (1.22, 1.33)
	South Central	1.42 (1.36, 1.47)	1.33 (1.28, 1.38)	1.45 (1.40, 1.50)	1.36 (1.31, 1.41)	1.47 (1.42, 1.52)	1.38 (1.33, 1.43)	1.43 (1.38, 1.48)	1.34 (1.29, 1.40)
	South East Coast	1.06 (1.02, 1.10)	1.02 (0.98, 1.06)	1.07 (1.04, 1.11)	1.03 (0.99, 1.07)	1.11 (1.07, 1.15)	1.07 (1.03, 1.11)	1.05 (1.01, 1.09)	1.01 (0.97, 1.05)
Ethnicity	White	1	1	1	1	1	1	1	1
	South Asian	0.92 (0.88, 0.95)	0.99 (0.95, 1.03)	0.92 (0.88, 0.95)	0.99 (0.95, 1.03)	0.93 (0.90, 0.96)	0.98 (0.95, 1.02)	0.94 (0.91, 0.98)	1.02 (0.98, 1.06)
	Black	0.67 (0.64, 0.71)	0.83 (0.78, 0.88)	0.68 (0.64, 0.71)	0.83 (0.78, 0.88)	0.69 (0.65, 0.72)	0.83 (0.79, 0.87)	0.67 (0.64, 0.71)	0.83 (0.79, 0.88)
	Mixed	0.84 (0.77, 0.92)	0.91 (0.83, 0.99)	0.78 (0.72, 0.85)	0.84 (0.77, 0.92)	0.79 (0.73, 0.86)	0.86 (0.79, 0.93)	0.83 (0.76, 0.91)	0.90 (0.82, 0.99)
	Other	0.79 (0.73, 0.84)	0.85 (0.79, 0.91)	0.75 (0.70, 0.80)	0.81 (0.76, 0.86)	0.77 (0.73, 0.82)	0.83 (0.78, 0.88)	0.80 (0.75, 0.85)	0.86 (0.80, 0.92)
Maternal age, years	<20	0.87 (0.81, 0.92)	0.87 (0.82, 0.93)	0.87 (0.82, 0.93)	0.87 (0.82, 0.93)	0.68 (0.65, 0.72)	0.68 (0.64, 0.71)	0.88 (0.82, 0.93)	0.88 (0.83, 0.94)
	20-24	1	1	1	1	1	1	1	1
	25-29	1.13 (1.09, 1.17)	1.12 (1.08, 1.16)	1.14 (1.10, 1.18)	1.13 (1.10, 1.17)	1.24 (1.20, 1.28)	1.25 (1.21, 1.29)	1.13 (1.09, 1.17)	1.13 (1.09, 1.17)
	30-35	1.22 (1.18, 1.26)	1.21 (1.17, 1.25)	1.24 (1.20, 1.28)	1.23 (1.19, 1.27)	1.36 (1.32, 1.41)	1.38 (1.34, 1.42)	1.23 (1.19, 1.27)	1.22 (1.17, 1.26)
	≥35	1.17 (1.12, 1.21)	1.18 (1.13, 1.22)	1.19 (1.15, 1.23)	1.20 (1.15, 1.24)	1.18 (1.14, 1.21)	1.21 (1.17, 1.25)	1.16 (1.12, 1.21)	1.18 (1.14, 1.23)
	0	1	1	1	1	1	1	1	1

For peer review only - http://bmjopen.bmj.com/site/about/guidelines.xhtml

Number of children	1	0.80 (0.78, 0.82)	0.80 (0.78, 0.82)	0.84 (0.82, 0.86)	0.83 (0.81, 0.85)	0.87 (0.85, 0.89)	0.86 (0.84, 0.88)	0.79 (0.77, 0.80)	0.78 (0.76, 0.80)
	2	0.71 (0.68, 0.73)	0.71 (0.69, 0.74)	0.75 (0.72, 0.77)	0.74 (0.72, 0.77)	0.72 (0.70, 0.75)	0.71 (0.68, 0.73)	0.69 (0.67, 0.71)	0.69 (0.67, 0.72)
	≥3	0.61 (0.58, 0.63)	0.62 (0.59, 0.65)	0.64 (0.61, 0.67)	0.65 (0.62, 0.68)	0.63 (0.61, 0.66)	0.63 (0.60, 0.66)	0.59 (0.56, 0.61)	0.60 (0.57, 0.63)
Clinical risk group	No	1	1	1	1	1	1	1	1
	Yes	1.69 (1.62, 1.76)	1.70 (1.63, 1.77)	1.73 (1.66, 1.80)	1.73 (1.66, 1.80)	1.98 (1.91, 2.06)	2.00 (1.93, 2.07)	1.59 (1.53, 1.66)	1.60 (1.54, 1.67)
BMI	<18.5	0.85 (0.80, 0.90)		0.84 (0.79, 0.89)		0.84 (0.79, 0.88)		0.93 (0.88, 0.98)	
	18.5-24.9	1		1		1		1	
	25.0-29.9	1.04 (1.01, 1.07)		1.03 (1.01, 1.06)		1.04 (1.02, 1.07)		0.98 (0.95, 1.00)	
	≥30	1.00 (0.97, 1.03)		0.99 (0.96, 1.02)		1.03 (1.00, 1.06)		0.90 (0.87, 0.92)	

Note: All models include women who registered in first trimester, and exclude those with outcome unknown and missing ethnicity; minimally adjusted model of BMI excludes women with missing BMI

Abbreviations: UK, United Kingdom; IMD, Index of Multiple Deprivation; BMI, body mass index

For peer review only

For peer review only - http://bmjopen.bmj.com/site/about/guidelines.xhtml

Supplementary Table 7: Secondary analysis of subsequent pertussis vaccine uptake among women who had received pertussis vaccination in their first eligible pregnancy and had a second eligible pregnancy within the study period (N=3,111)

		**Total (column %)**	**Received pertussis vaccine in second pregnancy (row %)**	**Minimally adjusted model** OR of receiving vaccine in second pregnancy (95% CI)	**Fully adjusted model** OR of receiving vaccine in second pregnancy (95% CI)
N		3,111	1,877 (60.3)		
Year of first pregnancy	2012	550 (17.7)	380 (69.1)	1	1
	2013	1,912 (61.5)	1,264 (66.1)	0.87 (0.71-1.07)	0.70 (0.56-0.87)
	2014-15	649 (20.9)	233 (35.9)	0.25 (0.20-0.32)	0.14 (0.10-0.18)
Index of multiple deprivation (IMD) quintile	Least deprived	857 (27.6)	539 (62.9)	1	1
	2	539 (17.3)	326 (60.5)	0.90 (0.71-1.13)	0.91 (0.71-1.16)
	3	604 (19.4)	381 (63.1)	1.03 (0.92-1.28)	1.06 (0.83-1.35)
	4	579 (18.6)	337 (58.2)	0.82(0.66-1.02)	0.89 (0.70-1.15)
	Most deprived	532 (17.1)	294 (55.3)	0.72 (0.57-0.90)	0.77 (0.59-1.01)
Region	London	453 (14.6)	260 (57.4)	1	1
	North East	35 (1.1)	22 (62.9)	1.25 (0.65-2.83)	2.08 (0.95-4.58)
	North West	390 (12.5)	240 (61.5)	1.16 (0.87-1.55)	1.29 (0.95-1.77)
	Yorkshire & The Humber	31 (1.0)	14 (45.2)	0.56 (0.27-1.19)	0.73 (0.33-1.62)
	East Midlands	0	0	-	-
	West Midlands	375 (12.1)	229 (61.1)	1.13 (0.85-1.51)	1.33 (0.97-1.81)
	East of England	296 (9.5)	201 (67.9)	1.57 (1.14-2.15)	1.54 (1.10-2.16)
	South West	388 (12.5)	239 (61.6)	1.19 (0.98-1.58)	1.31 (0.96-1.79)
	South Central	562 (18.1)	360 (64.1)	1.33 (1.02-1.73)	1.31 (0.99-1.74)
	South East Coast	581 (18.7)	312 (53.7)	0.90 (0.69-1.16)	0.99 (0.75-1.31)
Ethnicity	White	2,732 (87.8)	1,657 (60.7)	1	1
	South Asian	204 (6.6)	114 (55.9)	0.82 (0.61-1.10)	0.78 (0.57-1.07)
	Black	84 (2.7)	49 (58.3)	1.05 (0.66-1.67)	1.09 (0.66-1.80)
	Mixed	33 (1.1)	20 (60.6)	0.94 (0.46-1.94)	1.14 (0.63-2.07)
	Other	58 (1.9)	37 (63.8)	1.25 (0.71-2.20)	0.97 (0.46-2.06)
Maternal age, years	<20	102 (3.2)	40 (39.2)	0.48 (0.30-0.75)	0.48 (0.30-0.77)
	20-24	505 (16.2)	290 (57.4)	1	1
	25-29	1,002 (32.2)	592 (59.1)	1.07 (0.85-1.34)	1.09 (0.86-1.39)
	30-34	1,048 (33.7)	669 (63.8)	1.32 (1.05-1.65)	1.26 (0.98-1.61)
	≥35	454 (14.6)	286 (63.0)	1.29 (0.99-1.69)	1.26 (0.94-1.69)
Number of children	0	1,936 (62.2)	1,224 (63.2)	1	1
	1	714 (23.0)	405 (56.7)	0.78 (0.65-0.94)	0.75 (0.62-0.91)
	2	264 (8.5)	149 (56.4)	0.72 (0.55-0.95)	0.64 (0.48-0.85)
	≥3	197 (6.3)	99 (50.3)	0.56 (0.42-0.76)	0.50 (0.36-0.69)
Pregnancy interval (days from end of first pregnancy to start of second)	0-179	416 (13.4)	227 (54.6)	1	1
	180-359	749 (24.1)	476 (63.6)	1.25 (0.96-1.63)	1.11 (0.85-1.45)
	360-539	1,004 (32.3)	695 (69.2)	1.33 (1.03-1.71)	1.13 (0.86-1.47)
	540-719	624 (20.1)	373 (59.8)	0.65 (0.49-0.85)	0.54 (0.41-0.73)
	720+	318 (10.2)	106 (33.3)	0.19 (0.14-0.27)	0.16 (0.11-0.22)

Note: Among 3,363 women with two eligible pregnancies during follow up, excluded 2 with implausible (<0 days) spacing between the end of the first pregnancy and start of the second, and 250 with missing ethnicity data.

For peer review only - http://bmjopen.bmj.com/site/about/guidelines.xhtml

The RECORD statement – checklist of items, extended from the STROBE statement, that should be reported in observational studies using routinely collected health data.

	Item No.	STROBE items	Location in manuscript where items are reported	RECORD items	Location in manuscript where items are reported
**Title and abstract**					
	1	(a) Indicate the study's design with a commonly used term in the title or the abstract (b) Provide in the abstract an informative and balanced summary of what was done and what was found	Title and abstract	RECORD 1.1: The type of data used should be specified in the title or abstract. When possible, the name of the databases used should be included.<br><br>RECORD 1.2: If applicable, the geographic region and timeframe within which the study took place should be reported in the title or abstract.<br><br>RECORD 1.3: If linkage between databases was conducted for the study, this should be clearly stated in the title or abstract.	Title and abstract<br><br>Abstract<br><br>No new linkage conducted for the study (use of pre-linked data described in methods)
**Introduction**					
Background rationale	2	Explain the scientific background and rationale for the investigation being reported	Introduction pages 5-6		
Objectives	3	State specific objectives, including any prespecified hypotheses	Introduction page 6		
**Methods**					
Study Design	4	Present key elements of study design early in the paper	Abstract and methods page 7		
Setting	5	Describe the setting, locations, and relevant dates, including	Abstract and methods page 7		

		periods of recruitment, exposure, follow-up, and data collection			
Participants	6	*(a) Cohort study* - Give the eligibility criteria, and the sources and methods of selection of participants. Describe methods of follow-up<br>*Case-control study* - Give the eligibility criteria, and the sources and methods of case ascertainment and control selection. Give the rationale for the choice of cases and controls<br>*Cross-sectional study* - Give the eligibility criteria, and the sources and methods of selection of participants<br><br>*(b) Cohort study* - For matched studies, give matching criteria and number of exposed and unexposed<br>*Case-control study* - For matched studies, give matching criteria and the number of controls per case	Cohort – methods pages 7-8<br><br>N/A Cohort – no matching	RECORD 6.1: The methods of study population selection (such as codes or algorithms used to identify subjects) should be listed in detail. If this is not possible, an explanation should be provided.<br><br>RECORD 6.2: Any validation studies of the codes or algorithms used to select the population should be referenced. If validation was conducted for this study and not published elsewhere, detailed methods and results should be provided.<br><br>RECORD 6.3: If the study involved linkage of databases, consider use of a flow diagram or other graphical display to demonstrate the data linkage process, including the number of individuals with linked data at each stage.	Cohort – methods pages 7-8<br><br>N/A<br><br>No new data linkages
Variables	7	Clearly define all outcomes, exposures, predictors, potential confounders, and effect modifiers. Give diagnostic criteria, if applicable.	Methods pages 9-10	RECORD 7.1: A complete list of codes and algorithms used to classify exposures, outcomes, confounders, and effect modifiers should be provided. If these cannot be reported, an explanation should be provided.	Methods pages 9-10
Data sources/ measurement	8	For each variable of interest, give sources of data and details of methods of assessment (measurement).	Methods pages 9-10		

For peer review only

		Describe comparability of assessment methods if there is more than one group			
Bias	9	Describe any efforts to address potential sources of bias	Methods page 11-12		
Study size	10	Explain how the study size was arrived at	Methods page 6-8		
Quantitative variables	11	Explain how quantitative variables were handled in the analyses. If applicable, describe which groupings were chosen, and why	Methods page 10		
Statistical methods	12	(a) Describe all statistical methods, including those used to control for confounding<br>(b) Describe any methods used to examine subgroups and interactions<br>(c) Explain how missing data were addressed<br>(d) *Cohort study* - If applicable, explain how loss to follow-up was addressed<br>*Case-control study* - If applicable, explain how matching of cases and controls was addressed<br>*Cross-sectional study* - If applicable, describe analytical methods taking account of sampling strategy<br>(e) Describe any sensitivity analyses	Methods page 10-12<br>N/A<br>Methods page 12<br>Methods page 9<br>Methods page 12		
Data access and cleaning methods		..		RECORD 12.1: Authors should describe the extent to which the investigators had access to the database population used to create the study population.	Page 22 author contributions

For peer review only

				RECORD 12.2: Authors should provide information on the data cleaning methods used in the study.	Methods pages 9-10, and results page 14
Linkage		..		RECORD 12.3: State whether the study included person-level, institutional-level, or other data linkage across two or more databases. The methods of linkage and methods of linkage quality evaluation should be provided.	No data linkage – this study used pre-linked data only, as described in Methods page 9
**Results**					
Participants	13	(a) Report the numbers of individuals at each stage of the study (*e.g.*, numbers potentially eligible, examined for eligibility, confirmed eligible, included in the study, completing follow-up, and analysed)<br>(b) Give reasons for non-participation at each stage.<br>(c) Consider use of a flow diagram	Methods page 9, results page 14	RECORD 13.1: Describe in detail the selection of the persons included in the study (*i.e.,* study population selection) including filtering based on data quality, data availability and linkage. The selection of included persons can be described in the text and/or by means of the study flow diagram.	Methods page 9, results page 14
Descriptive data	14	(a) Give characteristics of study participants (*e.g.*, demographic, clinical, social) and information on exposures and potential confounders<br>(b) Indicate the number of participants with missing data for each variable of interest<br>(c) *Cohort study* - summarise follow-up time (*e.g.*, average and total amount)	Results page 14, Tables 1 and 2<br><br>Results page 14 and supplementary table 2<br><br>N/A		
Outcome data	15	*Cohort study* - Report numbers of outcome events or summary measures over time	Tables 1 and 2		

For peer review only

		Case-control study - Report numbers in each exposure category, or summary measures of exposure<br>*Cross-sectional study* - Report numbers of outcome events or summary measures			
Main results	16	(a) Give unadjusted estimates and, if applicable, confounder-adjusted estimates and their precision (e.g., 95% confidence interval). Make clear which confounders were adjusted for and why they were included<br>(b) Report category boundaries when continuous variables were categorized<br>(c) If relevant, consider translating estimates of relative risk into absolute risk for a meaningful time period	Tables 1 and 2<br><br>Tables 1 and 2<br><br>N/A		
Other analyses	17	Report other analyses done—e.g., analyses of subgroups and interactions, and sensitivity analyses	Methods page 12-13, supplementary tables 3-7		
**Discussion**					
Key results	18	Summarise key results with reference to study objectives	Discussion page 19		
Limitations	19	Discuss limitations of the study, taking into account sources of potential bias or imprecision. Discuss both direction and magnitude of any potential bias	Discussion page 19	RECORD 19.1: Discuss the implications of using data that were not created or collected to answer the specific research question(s). Include discussion of misclassification bias, unmeasured confounding, missing data, and changing eligibility over time, as they pertain to the study being reported.	Discussion page 19

For peer review only

Interpretation	20	Give a cautious overall interpretation of results considering objectives, limitations, multiplicity of analyses, results from similar studies, and other relevant evidence	Discussion pages 20-21		
Generalisability	21	Discuss the generalisability (external validity) of the study results	Discussion page 19 re other settings		
**Other Information**					
Funding	22	Give the source of funding and the role of the funders for the present study and, if applicable, for the original study on which the present article is based	Funding statement		
Accessibility of protocol, raw data, and programming code		..		RECORD 22.1: Authors should provide information on how to access any supplemental information such as the study protocol, raw data, or programming code.	Methods page 10

*Reference: Benchimol EI, Smeeth L, Guttmann A, Harron K, Moher D, Petersen I, Sørensen HT, von Elm E, Langan SM, the RECORD Working Committee. The REporting of studies Conducted using Observational Routinely-collected health Data (RECORD) Statement. *PLoS Medicine* 2015; in press.

*Checklist is protected under Creative Commons Attribution (CC BY) license.

For peer review only

Social determinants of pertussis and influenza vaccine uptake in pregnancy: a national cohort study in England using electronic health records

Journal:	*BMJ Open*
Manuscript ID	bmjopen-2020-046545.R1
Article Type:	Original research
Date Submitted by the Author:	09-Apr-2021

Complete List of Authors:	Walker, Jemma; London School of Hygiene and Tropical Medicine, Faculty of Epidemiology and Population Health; Public Health England, Statistics, Modelling and Economics Department<br>Rentsch, Christopher ; London School of Hygiene & Tropical Medicine, Faculty of Epidemiology and Population Health<br>McDonald, Helen; London School of Hygiene and Tropical Medicine, Faculty of Epidemiology and Population Health<br>Bak, JeongEun; London School of Hygiene and Tropical Medicine, Faculty of Epidemiology and Population Health<br>Minassian, Caroline ; London School of Hygiene & Tropical Medicine, Faculty of Epidemiology and Population Health<br>Amirthalingam, Gayatri; Public Health England Immunisation and Countermeasures Division<br>Edelstein, Michael; Public Health England Immunisation and Countermeasures Division; London School of Hygiene & Tropical Medicine, Faculty of Epidemiology and Population Health<br>Thomas, Sara; London School of Hygiene & Tropical Medicine, Faculty of Epidemiology & Population Health

**Primary Subject Heading**:	Public health
Secondary Subject Heading:	Epidemiology, Health services research, Obstetrics and gynaecology
Keywords:	Public health < INFECTIOUS DISEASES, Maternal medicine < OBSTETRICS, PRIMARY CARE, PUBLIC HEALTH

SCHOLARONE™ Manuscripts

I, the Submitting Author has the right to grant and does grant on behalf of all authors of the Work (as defined in the below author licence), an exclusive licence and/or a non-exclusive licence for contributions from authors who are: i) UK Crown employees; ii) where BMJ has agreed a CC-BY licence shall apply, and/or iii) in accordance with the terms applicable for US Federal Government officers or employees acting as part of their official duties; on a worldwide, perpetual, irrevocable, royalty-free basis to BMJ Publishing Group Ltd ("BMJ") its licensees and where the relevant Journal is co-owned by BMJ to the co-owners of the Journal, to publish the Work in this journal and any other BMJ products and to exploit all rights, as set out in our licence.

The Submitting Author accepts and understands that any supply made under these terms is made by BMJ to the Submitting Author unless you are acting as an employee on behalf of your employer or a postgraduate student of an affiliated institution which is paying any applicable article publishing charge ("APC") for Open Access articles. Where the Submitting Author wishes to make the Work available on an Open Access basis (and intends to pay the relevant APC), the terms of reuse of such Open Access shall be governed by a Creative Commons licence – details of these licences and which Creative Commons licence will apply to this Work are set out in our licence referred to above.

Other than as permitted in any relevant BMJ Author's Self Archiving Policies, I confirm this Work has not been accepted for publication elsewhere, is not being considered for publication elsewhere and does not duplicate material already published. I confirm all authors consent to publication of this Work and authorise the granting of this licence.

For peer review only

Social determinants of pertussis and influenza vaccine uptake in pregnancy: a national cohort study in England using electronic health records

Authors: Jemma L Walker (0000-0003-3728-9509),[1,2,3]* Christopher T Rentsch (0000-0002-1408-7907),[1,2]* Helen I McDonald (0000-0003-0576-2015),[1,2§] JeongEun Bak (0000-0001-7519-7539),[2] Caroline Minassian (0000-0001-9406-1928),[2] Gayatri Amirthalingam (0000-0003-2078-0975),[1,4] Michael Edelstein (0000-0002-7323-0806),[1,2,4] Sara L Thomas.[1,2]

*Joint first authorship

1. NIHR Health Protection Research Unit in Immunisation
2. Faculty of Epidemiology and Population Health, London School of Hygiene and Tropical Medicine, London, WC1E 7HT, UK
   a. Jemma L Walker research fellow, Christopher T Rentsch research fellow, Helen I McDonald clinical research fellow, JeongEun Bak MSc student, Caroline Minassian assistant professor, Michael Edelstein honorary associate professor, Sara L Thomas professor
3. Statistics, Modelling and Economics Department, Public Health England, London, NW9 5EQ, UK
   a. Jemma L Walker statistician
4. Immunisation and Countermeasures Division, National Infection Service, Public Health England, London, NW9 5EQ, UK
   a. Gayatri Amirthalingam consultant epidemiologist, Michael Edelstein consultant epidemiologist

§Corresponding author:
Helen I McDonald, PhD
London School of Hygiene & Tropical Medicine
Keppel Street
WC1E 7HT
London, UK
Email: helen.mcdonald@lshtm.ac.uk

Article type: Research
Keywords: Influenza vaccine; pertussis vaccine; vaccination coverage; social determinants of health; electronic health records.

Word count: 4,068

For peer review only - http://bmjopen.bmj.com/site/about/guidelines.xhtml

For peer review only

ABSTRACT

Objective To examine the social determinants of influenza and pertussis vaccine uptake among pregnant women in England.

Design Nationwide population-based cohort study

Setting The study used anonymised primary care data from the Clinical Practice Research Datalink and linked Hospital Episode Statistics secondary care data

Participants Pregnant women eligible for pertussis (2012 to 2015, n=68,090) or influenza (2010/11 to 2015/16, n=152,132) vaccination, 2012 to 2015 (pertussis) and 2010/11 to 2015/16 (influenza)

Main outcome measures Influenza and pertussis vaccine uptake

Results

Vaccine uptake in the first eligible pregnancy was 67.3% for pertussis, and 39.1% for influenza. Uptake of both vaccines varied by region, with lowest uptakes in London and the North East. Lower vaccine uptake was associated with greater deprivation: almost 10% lower in the most deprived quintiles compared with the least deprived for influenza (34.5% vs 44.0%), and almost 20% lower for pertussis (57.7% vs 76.0%). Lower uptake for both vaccines was also associated with non-white ethnicity (lowest among women of Black ethnicity), maternal age under 20 years, and a greater number of children in the household. The associations between all social factors and vaccine uptake were broadly unchanged in fully adjusted models, suggesting the social determinants of uptake were largely independent of one another.

For peer review only - http://bmjopen.bmj.com/site/about/guidelines.xhtml

Among 3,111 women vaccinated against pertussis in their first eligible pregnancy and pregnant again, 1,234 (40%) were not vaccinated in their second eligible pregnancy.

Conclusions

Targeting promotional campaigns to pregnant women who are younger, of non-white ethnicity, with more children, living in areas of greater deprivation or the London or North East regions, has potential to reduce vaccine-preventable disease among infants and pregnant women, and to reduce health inequalities. Vaccination promotion needs to be sustained across successive pregnancies. Further research is needed into whether the effectiveness of vaccine promotion strategies may vary according to social factors.

For peer review only

For peer review only - http://bmjopen.bmj.com/site/about/guidelines.xhtml

Article Summary

Strengths and limitations of this study

- This large cohort study explored the social determinants of influenza and pertussis vaccination among pregnant women across England.
- It considered a range of social determinants including maternal age, ethnicity, socio-economic status, number of children in the household and region.
- The CPRD/LSHTM pregnancy register was used to ascertain pregnancies and their timing from primary care records using detailed algorithms.
- We were unable to investigate vaccine uptake inequalities from 2016 onwards due to the lack of reliable data on vaccination in secondary care settings.

For peer review only

For peer review only - http://bmjopen.bmj.com/site/about/guidelines.xhtml

INTRODUCTION

Pertussis (whooping cough) and seasonal influenza are vaccine-preventable diseases. Influenza can have severe outcomes among pregnant women and young infants, including hospitalisation and death.[1] Pertussis can be a serious illness for young infants: a pertussis outbreak in 2012 resulted in 14 infant deaths, most of whom were too young to be vaccinated directly.[2-4] Vaccination in pregnancy reduces influenza-associated hospitalisation among pregnant women,[5] and provides 'passive immunity' to protect infants in the first months of life.[6, 7] In England, pertussis vaccination has been offered to women in later stages of pregnancy since 2010 and seasonal influenza vaccination at any stage of pregnancy during influenza season since 2012.[2, 8]

Low vaccine uptake during pregnancy is a major public health challenge for high-income countries.[9] According to routine surveillance in 2018/19, vaccine uptake amongst pregnant women in England was 68.8% for pertussis and 45.2% for influenza.[10, 11] Although comparatively high for a high-income country, this suboptimal uptake still limits the programme's impact and results in vaccine-preventable deaths among infants of unvaccinated mothers. Studies of determinants of maternal influenza vaccine uptake to date have largely focused on health beliefs.[12] Studies in the United States have found inequalities in vaccine uptake during pregnancy by ethnicity/race, age and insurance status.[13-15] Less is known about the role of social factors in England. During the 2009 influenza pandemic, higher vaccine uptake in pregnancy was associated with higher maternal age, previous deliveries, and underlying health conditions but not deprivation.[16] However, ecological studies suggest that both seasonal influenza and pertussis vaccine uptake in pregnancy vary with ethnicity, and are lower in areas with greater deprivation, and are thus sources

For peer review only - http://bmjopen.bmj.com/site/about/guidelines.xhtml

of health inequalities in infancy.[17, 18] Smaller studies of pertussis and seasonal influenza vaccines have suggested deprivation, ethnicity, maternal age and parity or number of children may be factors in maternal vaccine uptake, but have lacked power to describe these associations fully.[19-23] A better understanding of the social determinants of maternal vaccine uptake could inform targeted public health interventions to improve vaccine uptake and reduce health inequalities.

This study aimed to use linked electronic health records to examine the social determinants of influenza and pertussis vaccine uptake among pregnant women in England for the first few years from programme introduction: 2012 to 2015 for pertussis and 2010/11 to 2015/16 for influenza vaccination.

For peer review only

METHODS

Data sources

This historical cohort study used data from the Clinical Practice Research Datalink (CPRD), a quality-assured anonymised primary care patient dataset covering approximately 7% of general practices in England, a representative sample of the population by age and sex.[24, 25] Available data include diagnoses and symptoms, prescriptions, immunisations and referrals recorded in primary care. The CPRD/LSHTM Pregnancy Register details all pregnancies recorded in primary care, identified using detailed algorithms to determine their timing and outcomes.[26] The Pregnancy Register has a high sensitivity for livebirths but may under-record pregnancies which end in a loss.[26, 27] For this analysis, we used the Pregnancy Register and CPRD data pre-linked to Hospital Episode Statistics (HES) admissions data (for supplementary ethnicity data),[28] and Office of National Statistics (ONS) small-area-level deprivation data.[29]

Study population

Analysis of pertussis vaccine and seasonal influenza vaccine uptake were conducted separately. For each vaccine, we identified pregnancies eligible for the relevant vaccination among women registered with CPRD, using the Pregnancy Register to identify start and end dates of pregnancies, eligible dates based on gestation, and pregnancy outcomes. Eligible women were registered at one of the 75% of CPRD practices in England which participate in the CPRD data-linkage scheme, for availability of linked HES and ONS data.[24] Vaccine eligibility started on or after 1 October 2012 for the pertussis vaccine analyses, and on or after 1 April 2010 for the

For peer review only - http://bmjopen.bmj.com/site/about/guidelines.xhtml

seasonal influenza vaccine analyses, reflecting the introduction of vaccination programmes.[2, 8] For each vaccine, the first eligible pregnancy for each woman during the follow-up period was used to avoid non-independence in the data.

Vaccination guidelines during the study period suggested women be offered pertussis vaccination in their third trimester of pregnancy (ideally between 28-32 weeks, though it could be offered between 28-38 weeks' gestation).[2, 8] For the pertussis vaccine analyses, we included women who delivered a live-or stillborn child on or after 26 weeks of pregnancy and followed up for vaccination up to 40 weeks' gestation, which allowed for up to 2 weeks imprecision in the Pregnancy Register estimation of the vaccine eligible period and mirrored the national surveillance approach. The study period ended before the April 2016 change in guidelines recommending vaccination at 16-32 weeks of pregnancy (though it may be given up to delivery), and changes in the commissioning arrangements leading to increased delivery through maternity services from 2016.[2]

Influenza vaccination is recommended at any stage in pregnancy that overlaps with the influenza season.[8] For the influenza vaccine analyses, all pregnancies for which the Pregnancy Register included a known outcome (such as stillbirth, livebirth, miscarriage, or termination) were included, irrespective of duration of pregnancy, providing the pregnancy overlapped by at least one day with the influenza season (1 September to 31 January of each year).

We limited primary analyses for both maternal vaccines to women who registered as patients at the primary care practice by the end of their first trimester, to reduce misclassification of vaccination status. We conducted sensitivity analyses around the study inclusion criteria, which are described below.

For peer review only

Follow-up period

The study period ranged from 1 October 2012 to 30 September 2015 for pertussis vaccine and 1 September 2010 to 31 January 2016 for influenza vaccine. Start of follow-up was considered the latest date of: start of the study period, practice meeting CPRD quality standards, patient registration at the practice, 11th birthday (dates of birth based on the mid-point of year of birth), 26 weeks gestation of pregnancy (for pertussis), the start of pregnancy plus 2 weeks (for influenza), or 1st September of each year (for influenza). End of follow-up was the earliest date of: last data collection from the practice, end of linkage to HES, patient transfer out of the practice, 49th birthday, death, receipt of the vaccine of interest, the 40th week of pregnancy (for pertussis), end of pregnancy (for influenza), end of the study period, or 31 January of each year (for influenza).

Vaccine uptake

Vaccination status for both maternal pertussis and influenza vaccines was extracted from CPRD. For the primary analysis of pertussis vaccine uptake, women were considered vaccinated if they received the vaccine between 26 and 40 weeks of pregnancy gestation, which is similar to the national vaccination guidelines of 28 to 38 weeks but allows for up to two weeks discrepancy in the Pregnancy Register estimation of gestation. Women who were not vaccinated between 26 and 40 weeks of gestation were considered unvaccinated, irrespective of vaccination before 26 weeks or after 40 weeks of gestation. For the primary analysis of influenza vaccine uptake, women were considered vaccinated if they received the vaccine on any day between 1 September and 31 January during their follow-up period. Women with a

For peer review only - http://bmjopen.bmj.com/site/about/guidelines.xhtml

pregnancy that spanned two influenza seasons (n=19,963, 14%) were counted in the denominator of the latter season and considered vaccinated if vaccinated in either season.

Social characteristics and clinical conditions

We defined social determinants using previously published detailed algorithms.[30] Index of multiple deprivation (IMD, a composite measure of relative deprivation) was assigned in quintiles (1 representing least deprived, 5 most deprived) based on the Lower Super Output Area of the patient's residential address using ONS national statistics data.[29] Ethnicity (White, South Asian, Black, Mixed, Other) was defined using primary care records supplemented with linked HES data.[28] Other social factors of interest were defined using CPRD primary care data and comprised: region of residence (London, North East, North West, Yorkshire & The Humber, East Midlands, West Midlands, East of England, South West, South Central, and South East Coast), maternal age (based on midpoint of year of birth), and number of children in the household.

For influenza vaccine uptake analyses, whether the individual was in a clinical risk group indicated to receive influenza vaccine was defined according to national guidance,[8] and comprised the following conditions: chronic renal disease, chronic heart disease, chronic respiratory disease, chronic liver disease, diabetes, immunosuppression, chronic neurological disease, asplenia, and morbid obesity. Clinical risk groups were identified using Read codes, primary care prescription records (for immunosuppression and asthma), and height and weight records. Body mass index (BMI) was defined using height and weight records using validated

For peer review only - http://bmjopen.bmj.com/site/about/guidelines.xhtml

methods,[31] and defined based on the record closest to the beginning of pregnancy, allowing measures during the first trimester of pregnancy. Asthma was defined as an asthma diagnosis and either any history of an emergency hospital admission for asthma, or any inhaled or oral steroid prescription in the previous 12 months. The algorithms used for immunosuppression are described in previous studies;[32] codelists for other conditions are available from https://doi.org/10.17037/DATA.00001907.

Statistical analysis

Parallel analyses were conducted for pertussis and influenza vaccine uptake. For each vaccine, a complete case analysis (excluding women with no ethnicity recorded in the main analysis) using multivariable logistic regression was used to estimate associations between vaccine uptake and social determinants. Our modelling strategy followed a previously adapted version[33] of a conceptual framework to analyse the hierarchical inter-relationships between distal and proximate social determinants with vaccine uptake **(Supplementary Table 1)**.[34] We first fitted a 'minimally adjusted' model to estimate associations between each social determinant and vaccine uptake adjusted for year (calendar year for pertussis, financial year for influenza to reflect the influenza season) to adjust for secular trends as an *a priori* confounder. We then fitted five further sequential models. Models 1 to 3 explored the social determinants of uptake from distal to proximal. Model 4 and the BMI Model explored the extent to which these were mediated by clinical conditions (for influenza), and mediated and/or confounded by BMI (for both vaccines).

In Model 1 we assessed associations between vaccine uptake and the distal determinants IMD, region, and ethnicity, mutually adjusted and adjusted for year. In

For peer review only - http://bmjopen.bmj.com/site/about/guidelines.xhtml

For peer review only

Model 2 the intermediate variable maternal age was added alongside the variables in Model 1 to determine to what extent this explained any effect of the distal variables. Model 3 comprised the variables in Model 2 and the proximate variable number of children, to investigate whether this mediated the effect of the distal and intermediate variables. For influenza uptake modelling, we further added clinical risk group as a potential mediator of the social characteristics (Model 4). Finally, we repeated complete case analyses additionally excluding women with no recorded BMI for all four models, adding a further model (BMI Model) that additionally adjusted for BMI, which may both mediate and confound the effect of social characteristics and clinical conditions.

All analyses were conducted using Stata 15 (StataCorp, College Station, TX, USA).

Missing data and sensitivity analyses

Primary analyses were conducted on women who had non-missing ethnicity and who were registered with an up-to-standard CPRD practice by the end of their first trimester. Other than ethnicity, only BMI had missing data.

We performed descriptive and sensitivity analyses to understand how estimates of vaccine uptake and associations with social determinants might be affected by missing data or study inclusion criteria. First, we examined the distribution of social determinants among women with and without recorded ethnicity. Second, we compared estimates from minimally and fully adjusted models from the primary analyses with sensitivity analyses including women who registered with an up-to-standard practice by the end of pregnancy (instead of end of first trimester) for both vaccines. For the pertussis analyses, we further ran minimally and fully adjusted models that mirrored national surveillance criteria of immunisation at 28-38 weeks'

For peer review only - http://bmjopen.bmj.com/site/about/guidelines.xhtml

gestation, to assess the impact of allowing a two-week window for imprecise estimation of gestation in our primary analysis. For the influenza analyses, we further ran models that included pregnancies with no recorded outcome, as well as models that extended the influenza season through 31 March of each year. Finally, for both pertussis and influenza analyses, we fitted random effects models to test for clustering by general practice.

Secondary analysis of sequential pregnancies

In response to the finding that vaccine uptake declined with greater number of children in the household, a *post-hoc* secondary analysis was added investigating the social determinants associated with vaccination in a second eligible pregnancy among women who had received pertussis vaccination in their first eligible pregnancy. This analysis focused on pertussis vaccination, as influenza vaccination uptake may depend upon the extent and timing of the overlap of pregnancy with the influenza season, severity of the influenza season and timing of vaccine availability, reducing the number of eligible sequential pregnancies and increasing the complexity of external factors which may affect a women's vaccine uptake across sequential pregnancies. Logistic regression with likelihood ratio tests were used to model and test minimally adjusted and fully adjusted (Model 3) associations between the outcome (vaccination in the second eligible pregnancy) and social determinants measured at baseline of the first eligible pregnancy, as well as additionally adjusting for the time interval between the end of the first pregnancy and the start of the next.

Ethics and patient involvement

For peer review only - http://bmjopen.bmj.com/site/about/guidelines.xhtml

The study was approved by the Independent Scientific Advisory Group of the CPRD (ISAC, Reference: 17_030) with an amendment to include the secondary analysis (ISAC reference 17_030RA2) and the London School of Hygiene and Tropical Medicine Ethics Committee (Reference: 16265). The amended ISAC protocol was made available to reviewers. This research was conducted without patient involvement.

For peer review only

For peer review only - http://bmjopen.bmj.com/site/about/guidelines.xhtml

RESULTS

Sample characteristics

A total of 68,090 women from 402 general practices were eligible for the pertussis vaccine analysis, and 152,132 women from 456 general practices were eligible for the influenza vaccine analysis during the study period. Many women were eligible to be offered both pertussis and influenza vaccinations during the study: 66,143 women were included in both analytic samples (97.1% of the pertussis vaccine cohort and 43.5% of the influenza cohort). There were 5,553 (8.9%) and 11,991 (7.9%) women from the pertussis and influenza vaccine analyses, respectively, who had missing ethnicity and were excluded from analysis.

Compared to women with recorded ethnicity, women with missing ethnicity were more likely to have an eligible pregnancy later in the study period, reside in South Central or South East Coast regions of England, have no children living in their household, and to have missing BMI information. Vaccine uptake was similar between women with recorded versus missing ethnicity for pertussis (67.3% vs. 68.2) and influenza (39.1% vs. 40.4%) (all p<0.001, **Supplementary Table 2**).

Primary analyses – pertussis vaccination

Among 62,537 eligible women with recorded ethnicity, maternal pertussis vaccine uptake increased each year, reaching 71.7% in 2015 (**Table 1**). Uptake was also highest in the least deprived areas (76.0%, **Figure 1**) and East and West Midlands (74.5% and 72.9%, respectively), and among women of white ethnicity (69.0%), aged 30-35 years (70.8%), who had no other children living in household (74.4%), who were of normal weight or overweight (69.2% and 69.3%, respectively).

For peer review only - http://bmjopen.bmj.com/site/about/guidelines.xhtml

For peer review only

After adjusting for calendar year, those who resided in the most deprived areas had less than half the odds of vaccine uptake compared to those in the least deprived areas, and those in all regions of England apart from the North East had increased odds of uptake compared to London (**Table 1**). Pertussis vaccination uptake was appreciably lower among all non-white ethnic groups, with reduced odds of between 24% (South Asian) and 55% (Black ethnicity) compared to those of White ethnicity. The odds of vaccination increased non-linearly with maternal age; compared to women aged 20-24 years, women who were <20 years had 21% lower odds of receiving vaccination and there was an increased likelihood of vaccination among women aged ≥25 years, reaching 54% increased odds of uptake among those aged 30-35 years. Uptake decreased linearly with increasing numbers of children living in the household; 33% less likely among women with one child, 53% less likely among women with two children, and 65% less likely among women with three or more children (Table 1). Among the 55,871 women with available BMI data, calendar-year adjusted uptake was 29% less likely among women whose BMI was classified as underweight and 18% less likely among women classified as obese, compared to women with normal BMI (**Table 1**).

Associations in the minimally adjusted models were largely unchanged after additionally adjusting for IMD, region, and ethnicity (Model 1), maternal age (Model 2), and number of children (Model 3). Associations were slightly attenuated (>10% change) for some regions in England (i.e., East of England, South Central, and South East Coast) in Model 1 and Model 2, but not in Model 3. Similarly, associations of pertussis uptake were marginally attenuated in non-white ethnic groups by adjustment for IMD and region (Model 2). However, strong evidence of all these associations remained. Model estimates were also robust to the additional

For peer review only - http://bmjopen.bmj.com/site/about/guidelines.xhtml

adjustment for BMI in the subset of women with non-missing BMI (**Supplementary Table 3**).

Primary analyses – influenza vaccination

Similar to pertussis vaccination, maternal influenza vaccine uptake was highest (46%) by the end of the study period (the 2015/16 season) among the 140,141 eligible women with recorded ethnicity (**Table 2**). Uptake was also highest in the least deprived areas (44.0%, **Figure 1**), in the South Central and West Midlands regions (42.6% and 42.2%, respectively), and among women of white ethnicity (39.8%), aged 30-35 years (41.0%), who had no children living in household (43.0%), and who were overweight (40.4%). Women who were classified as being in a clinical risk group had the highest influenza vaccine uptake (50.9%) out of all subgroups.

Findings of associations between social determinants and influenza vaccine uptake were largely the same as those with pertussis uptake (**Table 2**). Women were 65% more likely to receive the influenza vaccination in the 2015/16 season compared to the 2010/11 season. Similarly, in influenza-season adjusted models, women who resided in the most deprived areas had 29% lower odds of receiving vaccination, and women in all regions outside of London were more likely to be vaccinated. Associations with ethnicity, maternal age, number of children, and BMI also mirrored those found in the pertussis uptake models, although the lower uptake seen with women of non-white ethnicity was less marked than that seen for pertussis vaccination. Women identified as being in a clinical risk group for influenza were 69% more likely to be vaccinated than those not in a clinical risk group. Associations

For peer review only - http://bmjopen.bmj.com/site/about/guidelines.xhtml

were robust throughout all subsequent models except for South Asian ethnicity and South East Coast regional residence, and remained after additional adjustment for clinical risk group in Model 4 (**Table 2**). Model estimates were also robust to the additional adjustment for BMI in the model excluding those with missing BMI (**Supplementary Table 4**).

Sensitivity analyses

Directions of associations and conclusions were robust to all sensitivity analysis for pertussis vaccination (**Supplementary Table 5**) and influenza vaccination (**Supplementary Table 6**), and we found no evidence of clustering at the practice level in the primary analysis models for either pertussis or influenza uptake (ρ=0.07, 95% CI 0.06-0.09 for pertussis, ρ=0.03, 95% CI 0.03-0.03 for influenza).

Secondary analysis

Among women who were included in the main study, there were 3,111 women who received pertussis vaccination in their first eligible pregnancy and who completed a second eligible pregnancy within the study period. Among these, 1,234 (39.7%) were not vaccinated in their second eligible pregnancy. Social determinants of vaccine uptake among women who had previously received vaccination in pregnancy were similar to those in the main analysis, with lower uptake in the second eligible pregnancy associated with younger maternal age at the first pregnancy, a greater number of children in the household and a longer interval between pregnancies (**Supplementary Table 7**).

For peer review only - http://bmjopen.bmj.com/site/about/guidelines.xhtml

DISCUSSION

Vaccine uptake in pregnancy over the study period was 67.3% for pertussis and 39.1% for influenza. Lower vaccine uptake was associated with greater deprivation: the gap in uptake between the least and most deprived quintiles was almost 10% for influenza, and almost 20% for pertussis. Lower uptake was also associated with non-white ethnicity (particularly Black ethnicity), maternal age under 20 years, and greater number of children in the household. The associations between all social factors and vaccine uptake were largely independent of one another. Among women eligible for pertussis vaccination in two pregnancies and vaccinated in the first, 40% were not vaccinated in their second eligible pregnancy.

To our knowledge, this is the first large study of fully individual-level social determinants of maternal vaccine uptake of seasonal influenza and pertussis in England. Our findings differ from a large national study which found no association between deprivation and pandemic influenza vaccine uptake in pregnancy (although vaccine uptake did increase with maternal age) but the previous study was in the context of the 2010 influenza pandemic.[16] The pattern of regional variation we observed is consistent with national surveillance and ecological studies, and lower vaccine uptake in London is seen more widely across the vaccination programme.[10, 11, 17, 18] For seasonal influenza and pertussis vaccines, previous studies have generally suggested associations consistent with those we observed for deprivation, ethnicity, maternal age and parity or number of children, but studies have been ecological or pseudo-individualised, or were underpowered for precise estimates.[17-21, 23] Our findings in a large and nationally representative dataset demonstrate that each of these factors is an independent individual-level determinant of maternal vaccine uptake, outside of a pandemic context.

For peer review only - http://bmjopen.bmj.com/site/about/guidelines.xhtml

The novel finding that 40% of women who had been vaccinated in their first eligible pregnancy were not in their second is surprising, and suggests that low vaccine uptake in pregnancy is not fully determined by fixed maternal attitudes to vaccination, but may reflect healthcare access or awareness of the need for vaccination in each pregnancy.

Strengths of this study include the use of the CPRD/LSHTM Pregnancy Register with linked hospital and mortality data and detailed algorithms to identify pregnancy timings and a range of individual-level social determinants among a nationally representative population.[30]

Key limitations include low representation from some regions (in particular the East Midlands), and that not all potentially relevant social factors were available, such as education and religion. We may have over-estimated vaccine uptake as the pregnancy register may not include all pregnancies which ended in a loss without coming to the attention of healthcare workers. We included only timely pertussis vaccinations (before 40 weeks' gestation) which may result in lower uptake estimates than pertussis vaccine uptake by delivery. Our study was also limited to vaccination recorded in primary care records, which could have resulted in some under-recording of influenza vaccination, although maternity-led vaccination services were rare before 2016, and GPs are required to document vaccinations given outside the surgery. To minimise misclassification we ended our study period prior to the introduction of pertussis vaccination in antenatal settings.

The large differences we observed in vaccine uptake by deprivation and ethnicity indicate a key opportunity to reduce health inequalities. Targeting interventions and improving access to vaccines through primary care and maternity services for

For peer review only - http://bmjopen.bmj.com/site/about/guidelines.xhtml

pregnant women who live in more deprived areas, are of non-white ethnicity, younger, or have more children may reduce health inequalities, improve overall vaccine uptake, and reduce vaccine-preventable deaths among women and children. In addition to targeted vaccination promotion, wider action is needed to address inequalities in access to timely antenatal care.[36] The drop-off in uptake in second pregnancies suggests a need for awareness-raising of the rationale for passive immunisation of infants and the need for vaccination in each pregnancy. Communications to emphasise the need for vaccination in every pregnancy should be available in a range of locally appropriate languages. Since 2016, pertussis vaccination has been available in maternity services, aiming to increase opportunities for vaccine uptake, and it will be important to ensure that healthcare worker training also captures the importance of vaccination in every pregnancy and to monitor the impact of delivery in alternative settings on inequalities in uptake.

Our study adds to international evidence of health inequalities in vaccination uptake in high-income countries. Studies in the United States have found inequalities in vaccine uptake by insurance type, race/ethnicity and education.[13-15] Our finding of large inequalities in vaccine uptake during pregnancy in England, despite universal healthcare which is free at the point of access, highlights the need for other high-income countries to investigate and address inequalities in vaccine uptake during pregnancy.

Further research is needed into interventions to reduce inequalities in vaccine uptake during pregnancy,[37] to ensure that future vaccine promotion of these and any future maternal vaccination programmes succeed in narrowing rather than widening the large and multi-faceted health inequalities in early years.

For peer review only - http://bmjopen.bmj.com/site/about/guidelines.xhtml

Acknowledgements

This work uses data provided by patients and collected by the NHS as part of their care and support and would not have been possible without access to this data. The NIHR recognises and values the role of patient data, securely accessed and stored, both in underpinning and leading to improvements in research and care.

Author Contributions

JLW and SLT conceived the main study, and CTR and HIM conceived the secondary analysis. JLW, CTR, HIM, CM, and SLT designed the study. JLW performed the data extraction and JLW and CTR performed the statistical analyses. JB, CTR and HIM designed the secondary analysis, for which JB and HIM performed the statistical analysis. All authors contributed to the interpretation of results. CTR and HIM drafted the manuscript, which all authors contributed to, revised critically, and approved. HIM is the guarantor. The corresponding author (JLW) attests that all listed authors meet authorship criteria and that no others meeting the criteria have been omitted.

Funding

The research was funded by the National Institute for Health Research (NIHR) Health Protection Research Unit (HPRU) in Immunisation (Grant number IS-HPU-1112-10096) at the London School of Hygiene and Tropical Medicine in partnership with Public Health England (PHE). The views expressed are those of the authors and not necessarily those of the NHS, the NIHR, the Department of Health and Social Care, or PHE.

For peer review only

For peer review only - http://bmjopen.bmj.com/site/about/guidelines.xhtml

Competing interests

All authors have completed the ICMJE uniform disclosure form at www.icmje.org/coi_disclosure.pdf and declare: JLW, CTR, HIM and SLT had financial support from the National Institute for Health Research (NIHR) Health Protection Research Unit (HPRU) in Immunisation for the submitted work; Public Health England Immunisation and Countermeasures Division has provided vaccine manufacturers with post-marketing surveillance reports on pneumococcal and meningococcal infection which the companies are required to submit to the UK Licensing Authority in compliance with their Risk Management Strategy, and a cost recovery charge is made for these reports; no other relationships or activities that could appear to have influenced the submitted work.

Ethics approval

The study was approved by the Independent Scientific Advisory Group of the CPRD (ISAC reference 17_030RA2) and the London School of Hygiene and Tropical Medicine Ethics Committee (LSHTM reference 16265). The study protocol was made available to reviewers.

Data sharing

The data used for this study were obtained from the Clinical Practice Research Datalink (CPRD). All data are available via an application to the Independent

For peer review only - http://bmjopen.bmj.com/site/about/guidelines.xhtml

Scientific Advisory Committee (see https://www.cprd.com/Data-access). Data acquisition is associated with a fee.

Transparency

The manuscript's guarantor (HIM) affirms that the manuscript is an honest, accurate, and transparent account of the study being reported; that no important aspects of the study have been omitted; and that any discrepancies from the study as planned have been explained.

Copyright/license

The Corresponding Author has the right to grant on behalf of all authors and does grant on behalf of all authors, a non-exclusive worldwide licence to the Publishers and its licensees in perpetuity, in all forms, formats and media (whether known now or created in the future), to i) publish, reproduce, distribute, display and store the Contribution, ii) translate the Contribution into other languages, create adaptations, reprints, include within collections and create summaries, extracts and/or, abstracts of the Contribution, iii) create any other derivative work(s) based on the Contribution, iv) to exploit all subsidiary rights in the Contribution, v) the inclusion of electronic links from the Contribution to third party material where-ever it may be located; and, vi) licence any third party to do any or all of the above.

For peer review only - http://bmjopen.bmj.com/site/about/guidelines.xhtml

References

1. Rasmussen SA, Jamieson DJ, Uyeki TM. Effects of influenza on pregnant women and infants. American journal of obstetrics and gynecology. 2012 Sep;207(3 Suppl):S3-8
2. Public Health England. Immunisation against Infectious Disease (the Green Book). Chapter 24: Pertussis 2016. Available from: https://assets.publishing.service.gov.uk/government/uploads/system/uploads/attachment_data/file/514363/Pertussis_Green_Book_Chapter_24_Ap2016.pdf.
3. van Hoek AJ, Campbell H, Amirthalingam G, Andrews N, Miller E. The number of deaths among infants under one year of age in England with pertussis: results of a capture/recapture analysis for the period 2001 to 2011. Euro Surveillance: Bulletin Europeen sur les Maladies Transmissibles = European Communicable Disease Bulletin. 2013;18(9)
4. Amirthalingam G, Gupta S, Campbell H. Pertussis immunisation and control in England and Wales, 1957 to 2012: a historical review. Euro Surveillance: Bulletin Europeen sur les Maladies Transmissibles = European Communicable Disease Bulletin. 2013;18(38)
5. Thompson MG, Kwong JC, Regan AK, Katz MA, Drews SJ, Azziz-Baumgartner E, et al. Influenza Vaccine Effectiveness in Preventing Influenza-associated Hospitalizations During Pregnancy: A Multi-country Retrospective Test Negative Design Study, 2010-2016. Clinical infectious diseases : an official publication of the Infectious Diseases Society of America. 2019 Apr 24;68(9):1444-53
6. Amirthalingam G, Campbell H, Ribeiro S, Fry NK, Ramsay M, Miller E, et al. Sustained Effectiveness of the Maternal Pertussis Immunization Program in England 3 Years Following Introduction. Clinical infectious diseases : an official publication of the Infectious Diseases Society of America. 2016 Dec 1;63(suppl 4):S236-s43
7. Dabrera G, Zhao H, Andrews N, Begum F, Green H, Ellis J, et al. Effectiveness of seasonal influenza vaccination during pregnancy in preventing influenza infection in infants, England, 2013/14. Euro Surveill. 2014 Nov 13;19(45):20959
8. Public Health England. Immunisation against Infectious Disease (the Green Book). Chapter 19: Influenza. Available from: https://www.gov.uk/government/publications/influenza-the-green-book-chapter-19.
9. Wiley KE, Leask J. Respiratory vaccine uptake during pregnancy. Lancet Respir Med. 2013 Mar;1(1):9-11
10. Public Health England. Pertussis vaccination programme for pregnant women update: vaccine coverage in England, January to March 2019 and 2018/19 annual coverage. Health Protection Report [Internet]. 2019; 13. Available from: https://assets.publishing.service.gov.uk/government/uploads/system/uploads/attachment_data/file/821145/hpr2619_prntl-prtsss_VC.pdf.
11. Public Health England. Seasonal influenza vaccine uptake in GP patients: winter season 2018 to 20192019. Available from: https://assets.publishing.service.gov.uk/government/uploads/system/uploads/attachment_data/file/804889/Seasonal_influenza_vaccine_uptake_in_GP_patients_1819.pdf.
12. Yuen CY, Tarrant M. Determinants of uptake of influenza vaccination among pregnant women - a systematic review. Vaccine. 2014 Aug 6;32(36):4602-13
13. Koepke R, Schauer SL, Davis JP. Measuring maternal Tdap and influenza vaccination rates: Comparison of two population-based methods. Vaccine. 2017 Apr 25;35(18):2298-302

For peer review only - http://bmjopen.bmj.com/site/about/guidelines.xhtml

14. Ding H, Black CL, Ball S, Fink RV, Williams WW, Fiebelkorn AP, et al. Influenza Vaccination Coverage Among Pregnant Women - United States, 2016-17 Influenza Season. MMWR Morb Mortal Wkly Rep. 2017 Sep 29;66(38):1016-22
15. Housey M, Zhang F, Miller C, Lyon-Callo S, McFadden J, Garcia E, et al. Vaccination with tetanus, diphtheria, and acellular pertussis vaccine of pregnant women enrolled in Medicaid--Michigan, 2011-2013. MMWR Morb Mortal Wkly Rep. 2014 Sep 26;63(38):839-42
16. Sammon CJ, McGrogan A, Snowball J, de Vries CS. Pandemic influenza vaccination during pregnancy: an investigation of vaccine uptake during the 2009/10 pandemic vaccination campaign in Great Britain. Human vaccines & immunotherapeutics. 2013 Apr;9(4):917-23
17. Byrne L, Ward C, White JM, Amirthalingam G, Edelstein M. Predictors of coverage of the national maternal pertussis and infant rotavirus vaccination programmes in England. Epidemiol Infect. 2018 Jan;146(2):197-206
18. Tessier E, Warburton F, Tsang C, Rafeeq S, Boddington N, Sinnathamby M, et al. Population-level factors predicting variation in influenza vaccine uptake among adults and young children in England, 2015/16 and 2016/17. Vaccine. 2018 May 31;36(23):3231-8
19. Carlisle N, Seed PT, Gillman L. Can common characteristics be identified as predictors for seasonal influenza vaccine uptake in pregnancy? A retrospective cohort study from a South London Hospital. Midwifery. 2019;72:67-73
20. Wilcox CR, Calvert A, Metz J, Kilich E, MacLeod R, Beadon K, et al. Determinants of Influenza and Pertussis Vaccination Uptake in Pregnancy: A Multicenter Questionnaire Study of Pregnant Women and Healthcare Professionals. The Pediatric infectious disease journal. 2019;38(6):625-30
21. McAuslane H, Utsi L, Wensley A, Coole L. Inequalities in maternal pertussis vaccination uptake: a cross-sectional survey of maternity units. Journal of public health (Oxford, England). 2018;40(1):121-8
22. Maher L, Hope K, Torvaldsen S, Lawrence G, Dawson A, Wiley K, et al. Influenza vaccination during pregnancy: coverage rates and influencing factors in two urban districts in Sydney. Vaccine. 2013 Nov 12;31(47):5557-64
23. Donaldson B, Jain P, Holder BS, Lindsey B, Regan L, Kampmann B. What determines uptake of pertussis vaccine in pregnancy? A cross sectional survey in an ethnically diverse population of pregnant women in London. Vaccine. 2015 Oct 26;33(43):5822-8
24. Herrett E, Gallagher AM, Bhaskaran K, Forbes H, Mathur R, van Staa T, et al. Data Resource Profile: Clinical Practice Research Datalink (CPRD). Int J Epidemiol. 2015 Jun;44(3):827-36
25. Williams T, van Staa T, Puri S, Eaton S. Recent advances in the utility and use of the General Practice Research Database as an example of a UK Primary Care Data resource. Ther Adv Drug Saf. 2012 Apr;3(2):89-99
26. Minassian C, Williams R, Meeraus WH, Smeeth L, Campbell OMR, Thomas SL. Methods to generate and validate a Pregnancy Register in the UK Clinical Practice Research Datalink primary care database. Pharmacoepidemiol Drug Saf. 2019 Jul;28(7):923-33
27. Walker JL, Grint DJ, Strongman H, Eggo RM, Peppa M, Minassian C, et al. UK prevalence of underlying conditions which increase the risk of severe COVID-19 disease: a point prevalence study using electronic health records. BMC Public Health. 2021 2021/03/11;21(1):484

For peer review only - http://bmjopen.bmj.com/site/about/guidelines.xhtml

28. Mathur R, Bhaskaran K, Chaturvedi N, Leon DA, vanStaa T, Grundy E, et al. Completeness and usability of ethnicity data in UK-based primary care and hospital databases. Journal of public health (Oxford, England). 2014 Dec;36(4):684-92
29. Department for Communities and Local Government. The English Index of Multiple Deprivation 2015 – Frequently Asked Questions 2016 [23 January 2020]. Available from: https://assets.publishing.service.gov.uk/government/uploads/system/uploads/attachment_data/file/579151/English_Indices_of_Deprivation_2015_-_Frequently_Asked_Questions_Dec_2016.pdf
30. Jain A, van Hoek AJ, Walker JL, Mathur R, Smeeth L, Thomas SL. Identifying social factors amongst older individuals in linked electronic health records: An assessment in a population based study. PLoS One. 2017;12(11):e0189038
31. Bhaskaran K, Forbes HJ, Douglas I, Leon DA, Smeeth L. Representativeness and optimal use of body mass index (BMI) in the UK Clinical Practice Research Datalink (CPRD). BMJ Open. 2013 Sep 13;3(9):e003389
32. Walker JL, Andrews NJ, Amirthalingam G, Forbes H, Langan SM, Thomas SL. Effectiveness of herpes zoster vaccination in an older United Kingdom population. Vaccine. 2018 04 19;36(17):2371-7
33. Jain A, Walker JL, Mathur R, Forbes HJ, Langan SM, Smeeth L, et al. Zoster vaccination inequalities: A population based cohort study using linked data from the UK Clinical Practice Research Datalink. PLoS One. 2018;13(11):e0207183
34. Victora CG, Huttly SR, Fuchs SC, Olinto MT. The role of conceptual frameworks in epidemiological analysis: a hierarchical approach. Int J Epidemiol. 1997 Feb;26(1):224-7
35. . !!! INVALID CITATION !!! 35
36. McDonald H, Moren C, Scarlett J. Health inequalities in timely antenatal care: audit of pre- and post-referral delays in antenatal bookings in London 2015–16. Journal of Public Health. 2020;42(4):801-15
37. Crocker-Buque T, Edelstein M, Mounier-Jack S. Interventions to reduce inequalities in vaccine uptake in children and adolescents aged <19 years: a systematic review. J Epidemiol Community Health. 2017 Jan;71(1):87-97

For peer review only - http://bmjopen.bmj.com/site/about/guidelines.xhtml

Table 1. Pertussis vaccine uptake by social characteristics amongst pregnant women in England, 2012 to 2015
N=62,537 from 402 practices. Overall vaccine uptake 42,099 (67.3%)

	**Total** (column %)	**Received pertussis vaccine** unadjusted coverage (row %)	**Minimally adjusted for year** "minimally adjusted" OR (95% CI)	**Model 1** Additionally adjusted for IMD, region, and ethnicity OR (95% CI)	**Model 2** Additionally adjusted for maternal age OR (95% CI)	**Model 3** Additionally adjusted for number of children "fully adjusted" OR (95% CI)
**Year**						
2012	6,717 (10.7%)	3,809 (56.7%)	1	1	1	1
2013	24,657 (39.4%)	16,749 (67.9%)	1.62 (1.53, 1.71)	1.66 (1.57, 1.75)	1.66 (1.57, 1.75)	1.69 (1.60, 1.79)
2014	20,148 (32.2%)	13,638 (67.7%)	1.60 (1.51, 1.69)	1.63 (1.54, 1.73)	1.63 (1.54, 1.73)	1.66 (1.57, 1.76)
2015	11,015 (17.6%)	7,903 (71.7%)	1.94 (1.82, 2.07)	2.00 (1.87, 2.13)	2.00 (1.87, 2.13)	2.03 (1.90, 2.17)
**Index of Multiple Deprivation (IMD) quintile**						
Least deprived	13,285 (21.2%)	10,090 (76.0%)	1	1	1	1
2	11,335 (18.1%)	8,064 (71.1%)	0.78 (0.74, 0.83)	0.79 (0.74, 0.83)	0.80 (0.75, 0.85)	0.81 (0.76, 0.86)
3	12,933 (20.7%)	8,807 (68.1%)	0.68 (0.64, 0.71)	0.68 (0.64, 0.72)	0.70 (0.66, 0.74)	0.73 (0.69, 0.77)
4	12,973 (20.7%)	8,205 (63.2%)	0.54 (0.52, 0.57)	0.56 (0.53, 0.59)	0.59 (0.56, 0.62)	0.64 (0.60, 0.67)
Most deprived	12,011 (19.2%)	6,933 (57.7%)	0.43 (0.41, 0.46)	0.45 (0.42, 0.47)	0.48 (0.45, 0.51)	0.54 (0.51, 0.57)
**Region**						
London	11,894 (19.0%)	7,239 (60.9%)	1	1	1	1
North East	1,185 (1.9%)	687 (58.0%)	0.91 (0.81, 1.03)	0.96 (0.85, 1.09)	1.00 (0.88, 1.13)	1.04 (0.92, 1.19)
North West	8,835 (14.1%)	5,873 (66.5%)	1.29 (1.22, 1.36)	1.28 (1.20, 1.35)	1.30 (1.22, 1.38)	1.36 (1.27, 1.44)
Yorkshire & The Humber	1,000 (1.6%)	699 (69.9%)	1.51 (1.31, 1.74)	1.46 (1.27, 1.69)	1.51 (1.30, 1.74)	1.54 (1.33, 1.79)
East Midlands	326 (0.5%)	243 (74.5%)	2.18 (1.69, 2.81)	2.24 (1.73, 2.90)	2.30 (1.78, 2.98)	2.38 (1.84, 3.09)
West Midlands	7,050 (11.3%)	5,046 (71.6%)	1.64 (1.54, 1.75)	1.58 (1.48, 1.69)	1.62 (1.52, 1.73)	1.72 (1.61, 1.84)
East of England	5,568 (8.9%)	4,058 (72.9%)	1.75 (1.63, 1.88)	1.50 (1.40, 1.61)	1.52 (1.41, 1.63)	1.57 (1.46, 1.69)
South West	7,002 (11.2%)	4,800 (68.6%)	1.43 (1.34, 1.52)	1.32 (1.24, 1.41)	1.35 (1.26, 1.44)	1.43 (1.33, 1.52)
South Central	10,381 (16.6%)	7,185 (69.2%)	1.45 (1.37, 1.53)	1.19 (1.12, 1.26)	1.21 (1.15, 1.29)	1.28 (1.21, 1.36)
South East Coast	9,296 (14.9%)	6,269 (67.4%)	1.33 (1.26, 1.41)	1.10 (1.04, 1.17)	1.12 (1.06, 1.19)	1.19 (1.12, 1.26)
**Ethnicity**						
White	52,598 (84.1%)	36,272 (69.0%)	1	1	1	1
South Asian	4,692 (7.5%)	2,951 (62.9%)	0.76 (0.71, 0.81)	0.83 (0.78, 0.88)	0.79 (0.74, 0.85)	0.83 (0.78, 0.88)
Black	2,583 (4.1%)	1,294 (50.1%)	0.45 (0.41, 0.48)	0.58 (0.54, 0.64)	0.56 (0.52, 0.61)	0.61 (0.56, 0.67)
Mixed	922 (1.5%)	549 (59.5%)	0.65 (0.57, 0.74)	0.72 (0.63, 0.82)	0.71 (0.62, 0.82)	0.72 (0.63, 0.83)

For peer review only - http://bmjopen.bmj.com/site/about/guidelines.xhtml

Other	1,742 (2.8%)	1,033 (59.3%)	0.65 (0.59, 0.72)	0.73 (0.66, 0.80)	0.70 (0.63, 0.77)	0.68 (0.62, 0.75)
**Maternal age, years**						
<20	2,079 (3.3%)	1,153 (55.5%)	0.79 (0.72, 0.87)		0.80 (0.73, 0.89)	0.81 (0.73, 0.89)
20-24	8,848 (14.1%)	5,416 (61.2%)	1		1	1
25-29	16,696 (26.7%)	11,166 (66.9%)	1.27 (1.21, 1.34)		1.24 (1.18, 1.31)	1.29 (1.22, 1.36)
30-35	20,294 (32.5%)	14,376 (70.8%)	1.54 (1.46, 1.62)		1.43 (1.35, 1.51)	1.55 (1.47, 1.64)
≥35	14,620 (23.4%)	9,988 (68.3%)	1.36 (1.29, 1.44)		1.25 (1.18, 1.32)	1.42 (1.34, 1.51)
**Number of children**						
0	26,622 (42.6%)	19,814 (74.4%)	1			1
1	22,132 (35.4%)	14,673 (66.3%)	0.67 (0.65, 0.70)			0.65 (0.63, 0.68)
2	8,645 (13.8%)	5,009 (57.9%)	0.47 (0.45, 0.49)			0.47 (0.45, 0.50)
≥3	5,138 (8.2%)	2,603 (50.7%)	0.35 (0.33, 0.37)			0.37 (0.35, 0.40)
**Body Mass Index (BMI)**						
<18.5 underweight	2,063 (3.3%)	1,265 (61.3%)	0.71 (0.64, 0.77)			
18.5-24.9	29,045 (46.4%)	20,095 (69.2%)	1			
25.0-29.9 overweight	14,211 (22.7%)	9,852 (69.3%)	1.01 (0.96, 1.05)			
≥30 obese	10,552 (16.9%)	6,833 (64.8%)	0.82 (0.78, 0.86)			
Missing	6,666 (10.7%)	4,054 (60.8%)				

OR, odds ratio; CI, confidence interval.

Note: All models include women who registered before the end of the first trimester and delivered a live-or stillborn child on or after 26 weeks of pregnancy and exclude those with missing ethnicity; minimally adjusted model of BMI additionally excludes 6,666 women with missing BMI

For peer review only - http://bmjopen.bmj.com/site/about/guidelines.xhtml

Table 2. Influenza vaccine uptake by social characteristics amongst pregnant women in England, 2010/11 to 2015/16
N=140,141 from 456 practices. Overall vaccine uptake 54,837 (39.1%)

	**Total** (column %)	**Received influenza vaccine** unadjusted coverage (row %)	**Minimally adjusted for year** "minimally adjusted" OR (95% CI)	**Model 1** Additionally adjusted for IMD, region, and ethnicity OR (95% CI)	**Model 2** Additionally adjusted for maternal age OR (95% CI)	**Model 3** Additionally adjusted for number of children OR (95% CI)	**Model 4** Additionally adjusted for clinical risk group "fully adjusted" OR (95% CI)
**Season**							
2010	34,373 (24.5%)	11,703 (34.0%)	1	1	1	1	1
2011	32,258 (23.0%)	10,151 (31.5%)	0.89 (0.86, 0.92)	0.89 (0.86, 0.92)	0.89 (0.86, 0.92)	0.89 (0.86, 0.92)	0.89 (0.86, 0.92)
2012	26,750 (19.1%)	12,236 (45.7%)	1.63 (1.58, 1.69)	1.66 (1.61, 1.72)	1.66 (1.61, 1.72)	1.64 (1.59, 1.70)	1.65 (1.60, 1.71)
2013	21,029 (15.0%)	8,815 (41.9%)	1.40 (1.35, 1.45)	1.43 (1.38, 1.48)	1.42 (1.37, 1.47)	1.39 (1.35, 1.45)	1.40 (1.35, 1.45)
2014	15,712 (11.2%)	7,319 (46.6%)	1.69 (1.63, 1.76)	1.74 (1.67, 1.80)	1.73 (1.67, 1.80)	1.69 (1.63, 1.76)	1.70 (1.63, 1.76)
2015	10,019 (7.1%)	4,613 (46.0%)	1.65 (1.58, 1.73)	1.72 (1.65, 1.80)	1.72 (1.64, 1.80)	1.68 (1.60, 1.76)	1.68 (1.60, 1.76)
**Index of Multiple Deprivation (IMD) quintile**							
Least deprived	28,956 (20.7%)	12,744 (44.0%)	1	1	1	1	1
2	25,424 (18.1%)	10,533 (41.4%)	0.90 (0.87, 0.93)	0.91 (0.88, 0.94)	0.92 (0.89, 0.95)	0.93 (0.89, 0.96)	0.92 (0.89, 0.95)
3	29,368 (21.0%)	11,670 (39.7%)	0.84 (0.81, 0.86)	0.84 (0.82, 0.87)	0.86 (0.83, 0.89)	0.88 (0.85, 0.91)	0.88 (0.85, 0.91)
4	28,520 (20.4%)	10,278 (36.0%)	0.71 (0.69, 0.74)	0.72 (0.69, 0.74)	0.74 (0.71, 0.77)	0.77 (0.74, 0.79)	0.76 (0.74, 0.79)
Most deprived	27,873 (19.9%)	9,612 (34.5%)	0.67 (0.65, 0.70)	0.66 (0.64, 0.68)	0.69 (0.66, 0.71)	0.73 (0.70, 0.76)	0.72 (0.70, 0.75)
**Region**							
London	26,171 (18.7%)	9,146 (34.9%)	1	1	1	1	1
North East	2,758 (2.0%)	989 (35.9%)	1.11 (1.02, 1.21)	1.16 (1.07, 1.27)	1.19 (1.09, 1.29)	1.21 (1.11, 1.31)	1.21 (1.11, 1.31)
North West	19,060 (13.6%)	7,870 (41.3%)	1.37 (1.32, 1.42)	1.39 (1.33, 1.45)	1.40 (1.35, 1.46)	1.43 (1.37, 1.49)	1.42 (1.36, 1.47)
Yorkshire & The Humber	2,840 (2.0%)	1,090 (38.4%)	1.27 (1.18, 1.38)	1.24 (1.15, 1.35)	1.26 (1.16, 1.37)	1.26 (1.16, 1.37)	1.26 (1.16, 1.37)
East Midlands	1,940 (1.4%)	717 (37.0%)	1.33 (1.21, 1.47)	1.37 (1.24, 1.51)	1.39 (1.26, 1.53)	1.41 (1.27, 1.55)	1.40 (1.27, 1.55)
West Midlands	15,846 (11.3%)	6,692 (42.2%)	1.41 (1.35, 1.46)	1.40 (1.34, 1.46)	1.41 (1.36, 1.47)	1.44 (1.38, 1.51)	1.43 (1.37, 1.49)
East of England	13,695 (9.8%)	5,468 (39.9%)	1.31 (1.26, 1.37)	1.23 (1.18, 1.29)	1.24 (1.19, 1.29)	1.25 (1.20, 1.31)	1.24 (1.19, 1.30)
South West	16,546 (11.8%)	6,504 (39.3%)	1.25 (1.20, 1.31)	1.22 (1.17, 1.28)	1.24 (1.19, 1.29)	1.27 (1.21, 1.32)	1.25 (1.20, 1.31)
South Central	21,435 (15.3%)	9,125 (42.6%)	1.42 (1.36, 1.47)	1.30 (1.25, 1.35)	1.31 (1.26, 1.36)	1.34 (1.29, 1.39)	1.33 (1.28, 1.38)
South East Coast	19,850 (14.2%)	7,236 (36.5%)	1.06 (1.02, 1.10)	0.99 (0.95, 1.03)	1.00 (0.96, 1.04)	1.02 (0.98, 1.06)	1.02 (0.98, 1.06)
**Ethnicity**							
White	117,469 (83.8%)	46,781 (39.8%)	1	1	1	1	1
South Asian	10,827 (7.7%)	4,103 (37.9%)	0.92 (0.88, 0.95)	0.98 (0.94, 1.02)	0.96 (0.92, 1.00)	0.98 (0.94, 1.02)	0.99 (0.95, 1.03)
Black	5,853 (4.2%)	1,837 (31.4%)	0.67 (0.64, 0.71)	0.81 (0.76, 0.86)	0.80 (0.75, 0.85)	0.83 (0.78, 0.88)	0.83 (0.78, 0.88)
Mixed	2,094 (1.5%)	757 (36.2%)	0.84 (0.77, 0.92)	0.90 (0.82, 0.99)	0.90 (0.82, 0.99)	0.91 (0.83, 0.99)	0.91 (0.83, 0.99)
Other	3,898 (2.8%)	1,359 (34.9%)	0.79 (0.73, 0.84)	0.85 (0.80, 0.91)	0.84 (0.78, 0.90)	0.83 (0.78, 0.89)	0.85 (0.79, 0.91)

For peer review only - http://bmjopen.bmj.com/site/about/guidelines.xhtml

**Maternal age, years**							
<20	5,536 (4.0%)	1,817 (32.8%)	0.87 (0.81, 0.92)		0.87 (0.82, 0.93)	0.87 (0.82, 0.93)	0.87 (0.82, 0.93)
20-24	21,663 (15.5%)	7,797 (36.0%)	1		1	1	1
25-29	37,985 (27.1%)	14,827 (39.0%)	1.13 (1.09, 1.17)		1.11 (1.07, 1.15)	1.12 (1.09, 1.16)	1.12 (1.08, 1.16)
30-35	43,777 (31.2%)	17,950 (41.0%)	1.22 (1.18, 1.26)		1.18 (1.14, 1.22)	1.21 (1.17, 1.26)	1.21 (1.17, 1.25)
≥35	31,180 (22.2%)	12,446 (39.9%)	1.17 (1.12, 1.21)		1.12 (1.08, 1.16)	1.19 (1.15, 1.24)	1.18 (1.13, 1.22)
**Number of children**							
0	66,112 (47.2%)	28,457 (43.0%)	1			1	1
1	45,969 (32.8%)	17,092 (37.2%)	0.80 (0.78, 0.82)			0.80 (0.78, 0.82)	0.80 (0.78, 0.82)
2	18,192 (13.0%)	6,242 (34.3%)	0.71 (0.68, 0.73)			0.72 (0.69, 0.74)	0.71 (0.69, 0.74)
≥3	9,868 (7.0%)	3,046 (30.9%)	0.61 (0.58, 0.63)			0.63 (0.60, 0.66)	0.62 (0.59, 0.65)
**Clinical risk group recommended for influenza vaccination**							
No	130,160 (92.9%)	49,752 (38.2%)	1				1
Yes	9,981 (7.1%)	5,085 (50.9%)	1.69 (1.62, 1.76)				1.70 (1.63, 1.77)
**Body Mass Index (BMI)**							
<18.5 Underweight	4,865 (3.5%)	1,744 (35.8%)	0.85 (0.80, 0.90)				
18.5-24.9	66,405 (47.4%)	26,331 (39.7%)	1				
25.0-29.9 Overweight	31,855 (22.7%)	12,882 (40.4%)	1.04 (1.01, 1.07)				
≥30 Obese	23,142 (16.5%)	9,222 (39.8%)	1.00 (0.97, 1.03)				
Missing	13,874 (9.9%)	4,658 (33.6%)					

OR, odds ratio; CI, confidence interval.
Note: All models include women who registered before the end of the first trimester, and exclude those with no recorded pregnancy outcome or missing ethnicity; minimally adjusted model of BMI additionally excludes 13,874 women with missing BMI

For peer review only - http://bmjopen.bmj.com/site/about/guidelines.xhtml

Figure legend

Figure 1: Unadjusted pertussis and influenza vaccine coverage in pregnancy, by deprivation

For peer review only

For peer review only - http://bmjopen.bmj.com/site/about/guidelines.xhtml

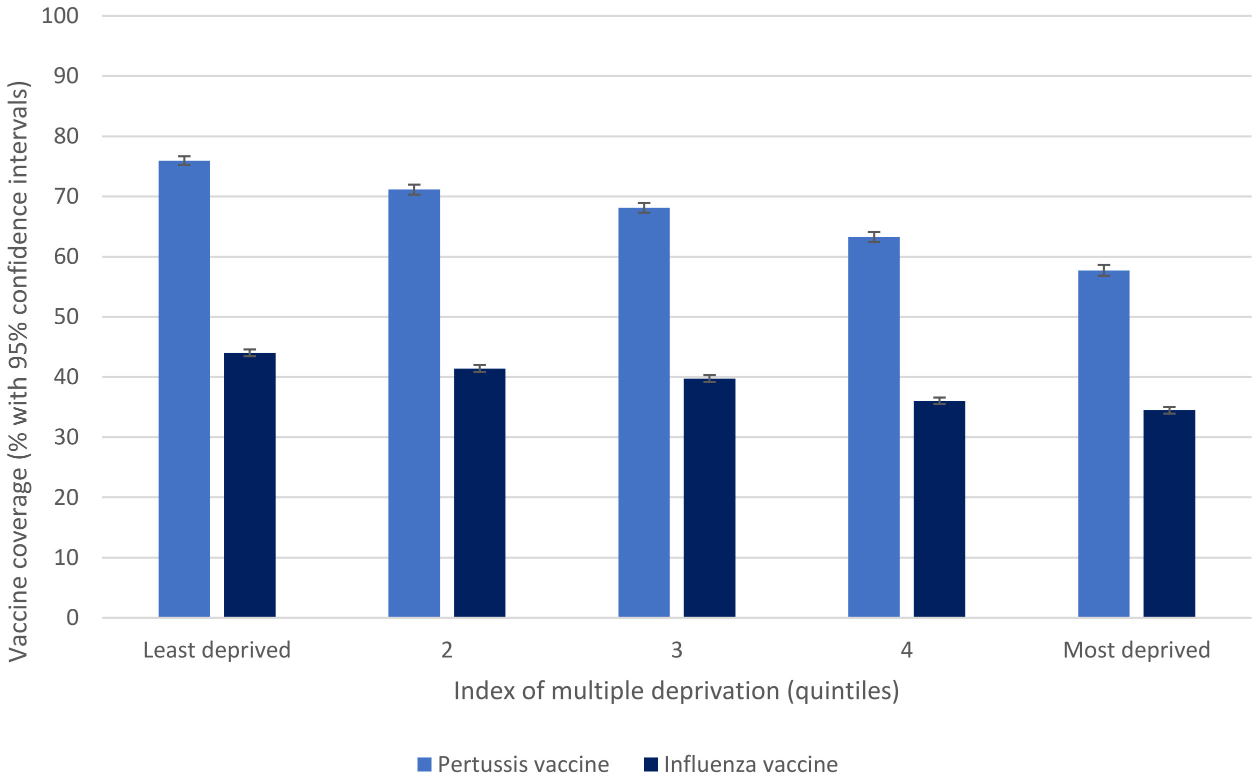
100
90
80
70
60
50
40
30
20
10
0
Vaccine coverage (% with 95% confidence intervals)
Least deprived
2
3
4
Most deprived
Index of multiple deprivation (quintiles)
Pertussis vaccine
Influenza vaccine

Supplementary material

Social determinants of pertussis and influenza vaccine uptake in pregnancy: a national cohort study using electronic health records

Authors: Jemma L Walker,* Christopher T Rentsch,* Helen I McDonald, Jeongeun Bak, Caroline Minassian, Gayatri Amirthalingam, Michael Edelstein, Sara L Thomas.

Supplementary Table 1: Hierarchical conceptual framework and interpretation of effect estimates

Supplementary Table 2: Patterns of social factors amongst pregnant women with and without a recorded ethnicity status, 2010-2015

Supplementary Table 3: 'Pertussis BMI Model' complete case analysis additionally excluding 6,666 women with missing BMI for pertussis vaccine uptake amongst pregnant women in the UK, 2012-2015

Supplementary Table 4: 'Influenza BMI Model' complete case analysis additionally excluding 13,874 women with missing BMI for influenza vaccine uptake amongst pregnant women in the UK, 2010-2015

Supplementary Table 5: Sensitivity analyses expanding definition of inclusion criteria for the pertussis vaccine uptake models: registration by end of pregnancy and ImmForm approach compared to primary analyses

Supplementary Table 7: Secondary analysis of subsequent pertussis vaccine uptake among women who had received pertussis vaccination in their first eligible pregnancy and had a second eligible pregnancy within the study period (N=3,111)

For peer review only - http://bmjopen.bmj.com/site/about/guidelines.xhtml

Supplementary Table 1: Hierarchical conceptual framework and interpretation of effect estimates

This table is reproduced from Supplementary Table 6 in Jain A., Walker JL, Forbes H, Langan S, Smeeth L, van Hoek AJ and Thomas SL. Zoster vaccination inequalities: A population based cohort study using linked data from the UK Clinical Practice Research Datalink. PLoS One 2018;13(11):e0207183. doi: 10.1371/journal.pone.0207183.

(based on [1])

**Hierarchical models**	**Explanatory variables**	**Interpretation of effect estimates**
`Minimally' adjusted model	Each explanatory variable adjusted in-turn for *a priori* confounders: year of birth and gender	Effect estimate of each variable adjusted for *a priori* confounders.
Model-1*^	Ethnicity +immigration status^ with *a priori* confounders	Effects of ethnicity and immigration status adjusted for each other and *a priori* confounders
Model-2*	Model-1+ patient-LSOA-level deprivation#	(i) Effects of ethnicity and immigration status not mediated via deprivation and adjusted for each other and *a priori* confounders<br>(ii) Effect of patient-LSOA-level deprivation adjusted for *a priori* confounders, ethnicity and immigration status
Model-3*	Model-2 + rest of the explanatory variables~	(i) Effect of ethnicity and immigration status not mediated via deprivation and other explanatory variables~ *<br>(ii) Effect of deprivation not mediated via other explanatory variables~*<br>(iii) Effect of other explanatory variables~ *

*all variables in the model adjusted for each other and *a priori* confounders: year of birth, sex and calendar period ^ethnicity and immigration status examined for multicollinearity LSOA Lower-layer Super Output Area # patient-LSOA-level and practice-LSOA-level deprivation were considered to be correlated therefore only patient-LSOA-level deprivation used ~ care home residence, living alone status and cohabitation status (living alone and cohabitation examined for multicollinearity)

1. Victora CG, Huttly SR, Fuchs SC, Olinto MT. The role of conceptual frameworks in epidemiological analysis: a hierarchical approach. Int J Epidemiol. 1997;26(1):224-7. PubMed PMID: 9126524.

For peer review only - http://bmjopen.bmj.com/site/about/guidelines.xhtml

Supplementary Table 2: Patterns of social factors amongst pregnant women with and without a recorded ethnicity status, 2010-2015

		Pertussis		Influenza	
		Recorded ethnicity	Missing ethnicity	Recorded ethnicity	Missing ethnicity
		n=62,537	n=5,553	n=140,141	n=11,991
Year/season	2010	-	-	34,373 (24.5%)	2,433 (20.3%)
	2011	-	-	32,258 (23.0%)	2,228 (18.6%)
	2012	6,717 (10.7%)	506 (9.1%)	26,750 (19.1%)	1,791 (14.9%)
	2013	24,657 (39.4%)	1,789 (32.2%)	21,029 (15.0%)	1,730 (14.4%)
	2014	20,148 (32.2%)	1,910 (34.4%)	15,712 (11.2%)	1,882 (15.7%)
	2015	11,015 (17.6%)	1,348 (24.3%)	10,019 (7.1%)	1,927 (16.1%)
Index of multiple deprivation (IMD) quintile	Least deprived	13,285 (21.2%)	1,522 (27.4%)	28,956 (20.7%)	3,203 (26.7%)
	2	11,335 (18.1%)	883 (15.9%)	25,424 (18.1%)	1,896 (15.8%)
	3	12,933 (20.7%)	992 (17.9%)	29,368 (21.0%)	2,245 (18.7%)
	4	12,973 (20.7%)	1,592 (28.7%)	28,520 (20.4%)	3,265 (27.2%)
	Most deprived	12,011 (19.2%)	564 (10.2%)	27,873 (19.9%)	1,382 (11.5%)
Region	London	11,894 (19.0%)	502 (9.0%)	26,171 (18.7%)	1,144 (9.5%)
	North East	1,185 (1.9%)	60 (1.1%)	2,758 (2.0%)	173 (1.4%)
	North West	8,835 (14.1%)	917 (16.5%)	19,060 (13.6%)	1,761 (14.7%)
	Yorkshire & The Humber	1,000 (1.6%)	5 (0.1%)	2,840 (2.0%)	24 (0.2%)
	East Midlands	326 (0.5%)	70 (1.3%)	1,940 (1.4%)	435 (3.6%)
	West Midlands	7,050 (11.3%)	530 (9.5%)	15,846 (11.3%)	1,231 (10.3%)
	East of England	5,568 (8.9%)	464 (8.4%)	13,695 (9.8%)	1,025 (8.5%)
	South West	7,002 (11.2%)	223 (4.0%)	16,546 (11.8%)	574 (4.8%)
	South Central	10,381 (16.6%)	1,692 (30.5%)	21,435 (15.3%)	3,215 (26.8%)
	South East Coast	9,296 (14.9%)	1,090 (19.6%)	19,850 (14.2%)	2,409 (20.1%)
Ethnicity	White	52,598 (84.1%)	-	117,469 (83.8%)	-
	South Asian	4,692 (7.5%)	-	10,827 (7.7%)	-
	Black	2,583 (4.1%)	-	5,853 (4.2%)	-
	Mixed	922 (1.5%)	-	2,094 (1.5%)	-
	Other	1,742 (2.8%)	-	3,898 (2.8%)	-
Maternal age, years	<20	2,079 (3.3%)	218 (3.9%)	5,536 (4.0%)	583 (4.9%)
	20-24	8,848 (14.1%)	914 (16.5%)	21,663 (15.5%)	2,014 (16.8%)
	25-29	16,696 (26.7%)	1,391 (25.0%)	37,985 (27.1%)	3,004 (25.1%)
	30-35	20,294 (32.5%)	1,673 (30.1%)	43,777 (31.2%)	3,639 (30.3%)
	≥35	14,620 (23.4%)	1,357 (24.4%)	31,180 (22.2%)	2,751 (22.9%)
Number of children	0	26,622 (42.6%)	2,645 (47.6%)	66,112 (47.2%)	6,255 (52.2%)
	1	22,132 (35.4%)	1,675 (30.2%)	45,969 (32.8%)	3,312 (27.6%)
	2	8,645 (13.8%)	679 (12.2%)	18,192 (13.0%)	1,431 (11.9%)
	≥3	5,138 (8.2%)	554 (10.0%)	9,868 (7.0%)	993 (8.3%)
Clinical risk group	No	-	-	130,160 (92.9%)	11,238 (93.7%)
	Yes	-	-	9,981 (7.1%)	753 (6.3%)
Body mass index (BMI)	<18.5	2,063 (3.3%)	201 (3.6%)	4,865 (3.5%)	434 (3.6%)
	18.5-24.9	29,045 (46.4%)	2,489 (44.8%)	66,405 (47.4%)	5,571 (46.5%)
	25.0-29.9	14,211 (22.7%)	1,203 (21.7%)	31,855 (22.7%)	2,563 (21.4%)
	≥30	10,552 (16.9%)	785 (14.1%)	23,142 (16.5%)	1,747 (14.6%)
	Missing	6,666 (10.7%)	875 (15.8%)	13,874 (9.9%)	1,676 (14.0%)
Note: all p<0.001					

For peer review only - http://bmjopen.bmj.com/site/about/guidelines.xhtml

Supplementary Table 3: 'Pertussis BMI Model' complete case analysis additionally excluding 6,666 women with missing BMI for pertussis vaccine uptake amongst pregnant women in the UK, 2012-2015

		**Minimally adjusted** for year	**Model 3 (fully adjusted in main analysis)** Adjusted for year, IMD, region, ethnicity, maternal age and number of children	**BMI Model** As Model 3 and additionally adjusted for BMI
N		55,871	55,871	55,871
Year	2012	1	1	1
	2013	1.65 (1.56, 1.75)	1.74 (1.63, 1.84)	1.74 (1.63, 1.84)
	2014	1.63 (1.54, 1.73)	1.70 (1.60, 1.81)	1.70 (1.60, 1.81)
	2015	1.95 (1.82, 2.08)	2.04 (1.90, 2.19)	2.04 (1.91, 2.19)
Index of multiple deprivation (IMD) quintile	Least deprived	1	1	1
	2	0.78 (0.73, 0.83)	0.81 (0.76, 0.86)	0.81 (0.76, 0.86)
	3	0.67 (0.64, 0.71)	0.72 (0.68, 0.77)	0.72 (0.68, 0.77)
	4	0.54 (0.51, 0.58)	0.63 (0.59, 0.67)	0.63 (0.59, 0.67)
	Most deprived	0.44 (0.41, 0.46)	0.53 (0.50, 0.57)	0.54 (0.50, 0.57)
Region	London	1	1	1
	North East	0.96 (0.84, 1.10)	1.12 (0.98, 1.29)	1.12 (0.97, 1.28)
	North West	1.30 (1.22, 1.38)	1.36 (1.28, 1.46)	1.36 (1.28, 1.46)
	Yorkshire & The Humber	1.49 (1.29, 1.72)	1.51 (1.30, 1.76)	1.51 (1.30, 1.76)
	East Midlands	1.87 (1.44, 2.42)	2.33 (1.78, 3.04)	2.31 (1.77, 3.02)
	West Midlands	1.60 (1.50, 1.71)	1.70 (1.59, 1.83)	1.70 (1.58, 1.82)
	East of England	1.73 (1.60, 1.86)	1.56 (1.44, 1.68)	1.56 (1.44, 1.68)
	South West	1.41 (1.32, 1.51)	1.42 (1.33, 1.53)	1.42 (1.33, 1.53)
	South Central	1.46 (1.37, 1.55)	1.29 (1.21, 1.37)	1.29 (1.21, 1.37)
	South East Coast	1.33 (1.26, 1.42)	1.18 (1.11, 1.26)	1.18 (1.11, 1.26)
Ethnicity	White	1	1	1
	South Asian	0.74 (0.70, 0.79)	0.83 (0.77, 0.89)	0.83 (0.77, 0.89)
	Black	0.45 (0.41, 0.49)	0.62 (0.57, 0.68)	0.62 (0.56, 0.67)
	Mixed	0.69 (0.60, 0.79)	0.75 (0.65, 0.87)	0.75 (0.65, 0.87)
	Other	0.63 (0.57, 0.70)	0.67 (0.60, 0.75)	0.68 (0.61, 0.75)
Maternal age, years	<20	0.85 (0.75, 0.96)	0.84 (0.74, 0.96)	0.85 (0.75, 0.97)
	20-24	1	1	1
	25-29	1.27 (1.20, 1.34)	1.28 (1.20, 1.36)	1.27 (1.20, 1.35)
	30-35	1.49 (1.41, 1.58)	1.51 (1.42, 1.60)	1.49 (1.41, 1.58)
	≥35	1.32 (1.24, 1.40)	1.37 (1.29, 1.46)	1.36 (1.28, 1.45)
Number of children	0	1	1	1
	1	0.67 (0.65, 0.70)	0.66 (0.63, 0.68)	0.66 (0.63, 0.68)
	2	0.47 (0.44, 0.49)	0.47 (0.45, 0.50)	0.47 (0.45, 0.50)
	≥3	0.35 (0.33, 0.38)	0.37 (0.35, 0.40)	0.37 (0.35, 0.40)
Body mass index (BMI)	<18.5	0.71 (0.64, 0.77)		0.77 (0.70, 0.85)
	18.5-24.9	1		1
	25.0-29.9	1.01 (0.96, 1.05)		1.10 (1.05, 1.15)
	≥30	0.82 (0.78, 0.86)		0.96 (0.91, 1.00)

Note: Model inclusion as per the main analysis but additionally excluding 6,666 women with missing BMI

For peer review only - http://bmjopen.bmj.com/site/about/guidelines.xhtml

Supplementary Table 4: 'Influenza BMI Model' complete case analysis additionally excluding 13,874 women with missing BMI for influenza vaccine uptake amongst pregnant women in the UK, 2010-2015

		**Minimally adjusted** for year	**Model 4** adjusted for year, IMD,region, ethnicity, maternal age, number of children and clinical risk group	**BMI Model** as Model 4 and additionally adjusted for BMI
N		126,267	126,267	126,267
Year	2010	1	1	1
	2011	0.90 (0.87, 0.93)	0.90 (0.87, 0.93)	0.90 (0.87, 0.93)
	2012	1.63 (1.57, 1.68)	1.64 (1.59, 1.70)	1.64 (1.59, 1.70)
	2013	1.41 (1.36, 1.46)	1.41 (1.35, 1.46)	1.40 (1.35, 1.46)
	2014	1.70 (1.63, 1.77)	1.70 (1.63, 1.77)	1.70 (1.63, 1.77)
	2015	1.66 (1.58, 1.74)	1.67 (1.60, 1.76)	1.67 (1.59, 1.76)
Index of multiple deprivation (IMD) quintile	Least deprived	1	1	1
	2	0.90 (0.87, 0.94)	0.92 (0.89, 0.95)	0.92 (0.88, 0.95)
	3	0.83 (0.80, 0.86)	0.87 (0.84, 0.90)	0.86 (0.83, 0.90)
	4	0.71 (0.69, 0.74)	0.76 (0.73, 0.79)	0.75 (0.73, 0.78)
	Most deprived	0.68 (0.65, 0.70)	0.72 (0.69, 0.75)	0.71 (0.69, 0.74)
Region	London	1	1	1
	North East	1.17 (1.07, 1.28)	1.26 (1.15, 1.37)	1.25 (1.14, 1.37)
	North West	1.40 (1.34, 1.45)	1.43 (1.37, 1.49)	1.43 (1.37, 1.49)
	Yorkshire & The Humber	1.28 (1.18, 1.39)	1.26 (1.16, 1.37)	1.25 (1.15, 1.36)
	East Midlands	1.29 (1.17, 1.43)	1.35 (1.22, 1.49)	1.34 (1.21, 1.49)
	West Midlands	1.41 (1.35, 1.47)	1.44 (1.37, 1.50)	1.43 (1.37, 1.49)
	East of England	1.30 (1.24, 1.36)	1.23 (1.17, 1.28)	1.22 (1.17, 1.28)
	South West	1.29 (1.23, 1.34)	1.28 (1.22, 1.34)	1.27 (1.22, 1.33)
	South Central	1.45 (1.39, 1.51)	1.35 (1.30, 1.41)	1.35 (1.29, 1.40)
	South East Coast	1.08 (1.04, 1.12)	1.03 (0.99, 1.07)	1.03 (0.99, 1.07)
Ethnicity	White	1	1	1
	South Asian	0.90 (0.87, 0.94)	0.99 (0.95, 1.03)	0.99 (0.95, 1.04)
	Black	0.66 (0.62, 0.70)	0.83 (0.78, 0.88)	0.82 (0.77, 0.87)
	Mixed	0.85 (0.78, 0.94)	0.92 (0.84, 1.01)	0.92 (0.84, 1.01)
	Other	0.78 (0.73, 0.84)	0.86 (0.80, 0.92)	0.87 (0.80, 0.93)
Maternal age, years	<20	0.90 (0.84, 0.98)	0.90 (0.83, 0.97)	0.91 (0.84, 0.98)
	20-24	1	1	1
	25-29	1.12 (1.08, 1.16)	1.11 (1.07, 1.15)	1.11 (1.07, 1.15)
	30-35	1.20 (1.16, 1.24)	1.19 (1.15, 1.23)	1.19 (1.14, 1.23)
	≥35	1.14 (1.10, 1.18)	1.15 (1.11, 1.20)	1.15 (1.10, 1.19)
Number of children	0	1	1	1
	1	0.80 (0.78, 0.82)	0.79 (0.77, 0.81)	0.79 (0.77, 0.81)
	2	0.70 (0.68, 0.73)	0.71 (0.68, 0.73)	0.70 (0.68, 0.73)
	≥3	0.61 (0.58, 0.64)	0.62 (0.59, 0.66)	0.62 (0.59, 0.65)
Clinical risk group	No	1	1	1
	Yes	1.69 (1.62, 1.76)	1.69 (1.62, 1.77)	1.68 (1.61, 1.76)
Body mass index (BMI)	<18.5	0.85 (0.80, 0.90)		0.89 (0.84, 0.95)
	18.5-24.9	1		1
	25.0-29.9	1.04 (1.01, 1.07)		1.07 (1.04, 1.10)
	≥30	1.00 (0.97, 1.03)		1.06 (1.03, 1.09)

Note: Model inclusion as per the main analysis but additionally excluding 13,874 women with missing BMI

For peer review only - http://bmjopen.bmj.com/site/about/guidelines.xhtml

Supplementary Table 5: Sensitivity analyses expanding definition of inclusion criteria for the pertussis vaccine uptake models: registration by end of pregnancy and ImmForm approach compared to primary analyses

		Primary analyses		Registered by end of pregnancy		ImmForm approach	
		**Minimally adjusted**	**Fully adjusted**	**Minimally adjusted**	**Fully adjusted**	**Minimally adjusted**	**Fully adjusted**
N		62,537	62,537	80,831	80,831	90,720	90,720
Year	2012	1	1	1	1	1	1
	2013	1.62 (1.53, 1.71)	1.69 (1.60, 1.79)	1.59 (1.52, 1.67)	1.65 (1.58, 1.73)	1.55 (1.48, 1.62)	1.60 (1.53, 1.67)
	2014	1.60 (1.51, 1.69)	1.66 (1.57, 1.76)	1.69 (1.61, 1.77)	1.72 (1.64, 1.81)	1.64 (1.57, 1.72)	1.67 (1.60, 1.75)
	2015	1.94 (1.82, 2.07)	2.03 (1.90, 2.17)	2.09 (1.98, 2.21)	2.13 (2.02, 2.26)	2.04 (1.94, 2.15)	2.07 (1.96, 2.19)
Index of multiple deprivation (IMD) quintile	Least deprived	1	1	1	1	1	1
	2	0.78 (0.74, 0.83)	0.81 (0.76, 0.86)	0.79 (0.76, 0.83)	0.83 (0.79, 0.87)	0.79 (0.76, 0.83)	0.83 (0.79, 0.87)
	3	0.68 (0.64, 0.71)	0.73 (0.69, 0.77)	0.71 (0.68, 0.74)	0.78 (0.74, 0.81)	0.70 (0.67, 0.73)	0.76 (0.73, 0.80)
	4	0.54 (0.52, 0.57)	0.64 (0.60, 0.67)	0.58 (0.56, 0.61)	0.69 (0.66, 0.73)	0.58 (0.56, 0.61)	0.69 (0.66, 0.72)
	Most deprived	0.43 (0.41, 0.46)	0.54 (0.51, 0.57)	0.46 (0.44, 0.49)	0.59 (0.56, 0.62)	0.46 (0.44, 0.48)	0.58 (0.55, 0.60)
Region	London	1	1	1	1	1	1
	North East	0.91 (0.81, 1.03)	1.04 (0.92, 1.19)	1.01 (0.90, 1.13)	1.17 (1.05, 1.32)	1.03 (0.93, 1.15)	1.21 (1.08, 1.35)
	North West	1.29 (1.22, 1.36)	1.36 (1.27, 1.44)	1.31 (1.25, 1.38)	1.41 (1.34, 1.49)	1.30 (1.24, 1.36)	1.40 (1.34, 1.48)
	Yorkshire & The Humber	1.51 (1.31, 1.74)	1.54 (1.33, 1.79)	1.48 (1.31, 1.68)	1.55 (1.37, 1.76)	1.44 (1.28, 1.62)	1.53 (1.35, 1.73)
	East Midlands	2.18 (1.69, 2.81)	2.38 (1.84, 3.09)	2.12 (1.70, 2.65)	2.36 (1.88, 2.96)	2.16 (1.75, 2.67)	2.43 (1.96, 3.02)
	West Midlands	1.64 (1.54, 1.75)	1.72 (1.61, 1.84)	1.61 (1.53, 1.70)	1.73 (1.63, 1.83)	1.55 (1.47, 1.63)	1.67 (1.58, 1.76)
	East of England	1.75 (1.63, 1.88)	1.57 (1.46, 1.69)	1.65 (1.55, 1.75)	1.49 (1.40, 1.58)	1.65 (1.56, 1.74)	1.49 (1.41, 1.58)
	South West	1.43 (1.34, 1.52)	1.43 (1.33, 1.52)	1.48 (1.41, 1.56)	1.49 (1.41, 1.57)	1.49 (1.42, 1.57)	1.51 (1.43, 1.59)
	South Central	1.45 (1.37, 1.53)	1.28 (1.21, 1.36)	1.54 (1.47, 1.62)	1.41 (1.34, 1.48)	1.51 (1.44, 1.58)	1.38 (1.32, 1.45)
	South East Coast	1.33 (1.26, 1.41)	1.19 (1.12, 1.26)	1.33 (1.26, 1.39)	1.24 (1.17, 1.30)	1.30 (1.24, 1.36)	1.21 (1.15, 1.27)
Ethnicity	White	1	1	1	1	1	1
	South Asian	0.76 (0.71, 0.81)	0.83 (0.78, 0.88)	0.78 (0.74, 0.83)	0.84 (0.79, 0.88)	0.78 (0.75, 0.82)	0.84 (0.80, 0.89)
	Black	0.45 (0.41, 0.48)	0.61 (0.56, 0.67)	0.46 (0.43, 0.50)	0.61 (0.57, 0.66)	0.47 (0.44, 0.51)	0.63 (0.59, 0.67)
	Mixed	0.65 (0.57, 0.74)	0.72 (0.63, 0.83)	0.64 (0.57, 0.71)	0.70 (0.62, 0.79)	0.63 (0.57, 0.70)	0.69 (0.62, 0.77)
	Other	0.65 (0.59, 0.72)	0.68 (0.62, 0.75)	0.66 (0.61, 0.71)	0.69 (0.63, 0.74)	0.65 (0.61, 0.70)	0.68 (0.63, 0.74)
Maternal age, years	<20	0.79 (0.72, 0.87)	0.81 (0.73, 0.89)	0.73 (0.67, 0.79)	0.73 (0.68, 0.79)	0.74 (0.68, 0.79)	0.74 (0.69, 0.80)
	20-24	1	1	1	1	1	1
	25-29	1.27 (1.21, 1.34)	1.29 (1.22, 1.36)	1.28 (1.23, 1.34)	1.30 (1.25, 1.37)	1.26 (1.21, 1.32)	1.28 (1.23, 1.34)
	30-35	1.54 (1.46, 1.62)	1.55 (1.47, 1.64)	1.55 (1.49, 1.62)	1.57 (1.50, 1.65)	1.54 (1.47, 1.60)	1.55 (1.48, 1.62)
	≥35	1.36 (1.29, 1.44)	1.42 (1.34, 1.51)	1.41 (1.35, 1.48)	1.48 (1.41, 1.55)	1.38 (1.32, 1.44)	1.44 (1.37, 1.51)
Number of children	0	1	1	1	1	1	1
	1	0.67 (0.65, 0.70)	0.65 (0.63, 0.68)	0.69 (0.66, 0.71)	0.67 (0.65, 0.69)	0.70 (0.67, 0.72)	0.68 (0.66, 0.70)
	2	0.47 (0.45, 0.49)	0.47 (0.45, 0.50)	0.50 (0.48, 0.52)	0.49 (0.47, 0.51)	0.50 (0.48, 0.52)	0.50 (0.48, 0.52)
	≥3	0.35 (0.33, 0.37)	0.37 (0.35, 0.40)	0.38 (0.36, 0.40)	0.39 (0.37, 0.42)	0.39 (0.37, 0.41)	0.40 (0.38, 0.42)
Body mass index (BMI)	<18.5	0.71 (0.64, 0.77)		0.69 (0.64, 0.75)		0.71 (0.66, 0.77)	
	18.5-24.9	1		1		1	
	25.0-29.9	1.01 (0.96, 1.05)		0.97 (0.94, 1.01)		0.98 (0.94, 1.01)	
	≥30	0.82 (0.78, 0.86)		0.82 (0.79, 0.85)		0.82 (0.79, 0.85)	

Note: All models include women who registered in first trimester and exclude those with missing ethnicity; minimally adjusted models of BMI excludes women with missing BMI

Abbreviations: UK, United Kingdom; IMD, Index of Multiple Deprivation; BMI, body mass index

For peer review only - http://bmjopen.bmj.com/site/about/guidelines.xhtml

Supplementary Table 6: Sensitivity analyses expanding definition of inclusion criteria for the influenza vaccine uptake models: registration by end of pregnancy, including pregnancies without known outcomes, extending influenza season to March, compared to primary analyses

		Primary analyses		Registered by end of pregnancy		Including pregnancies without known outcomes		Extending influenza season through March	
		Minimally adjusted	Fully adjusted	Minimally adjusted	Fully adjusted	Minimally adjusted	Fully adjusted	Minimally adjusted	Fully adjusted
N		140,141	140,141	153,782	153,782	191,950	191,950	140,141	140,141
Season	2010	1	1	1	1	1	1	1	1
	2011	0.89 (0.86, 0.92)	0.89 (0.86, 0.92)	0.89 (0.86, 0.92)	0.89 (0.86, 0.92)	0.92 (0.89, 0.95)	0.92 (0.89, 0.94)	0.93 (0.90, 0.96)	0.93 (0.90, 0.96)
	2012	1.63 (1.58, 1.69)	1.65 (1.60, 1.71)	1.62 (1.57, 1.67)	1.63 (1.58, 1.68)	1.55 (1.51, 1.60)	1.56 (1.52, 1.61)	1.81 (1.76, 1.87)	1.84 (1.78, 1.90)
	2013	1.40 (1.35, 1.45)	1.40 (1.35, 1.45)	1.41 (1.36, 1.45)	1.41 (1.36, 1.46)	1.36 (1.32, 1.41)	1.36 (1.32, 1.41)	1.57 (1.51, 1.62)	1.57 (1.51, 1.62)
	2014	1.69 (1.63, 1.76)	1.70 (1.63, 1.76)	1.71 (1.65, 1.77)	1.72 (1.65, 1.78)	1.64 (1.59, 1.70)	1.63 (1.58, 1.69)	1.88 (1.81, 1.95)	1.89 (1.82, 1.96)
	2015	1.65 (1.58, 1.73)	1.68 (1.60, 1.76)	1.67 (1.60, 1.75)	1.70 (1.63, 1.78)	1.61 (1.54, 1.68)	1.61 (1.55, 1.68)	1.86 (1.78, 1.94)	1.89 (1.81, 1.98)
IMD	Least deprived	1	1	1	1	1	1	1	1
	2	0.90 (0.87, 0.93)	0.92 (0.89, 0.95)	0.88 (0.86, 0.91)	0.90 (0.87, 0.94)	0.87 (0.84, 0.90)	0.89 (0.86, 0.92)	0.90 (0.87, 0.93)	0.91 (0.88, 0.95)
	3	0.84 (0.81, 0.86)	0.88 (0.85, 0.91)	0.83 (0.81, 0.86)	0.87 (0.84, 0.90)	0.83 (0.80, 0.85)	0.87 (0.85, 0.90)	0.83 (0.81, 0.86)	0.87 (0.84, 0.90)
	4	0.71 (0.69, 0.74)	0.76 (0.74, 0.79)	0.71 (0.69, 0.73)	0.76 (0.74, 0.79)	0.71 (0.69, 0.74)	0.78 (0.75, 0.80)	0.70 (0.68, 0.73)	0.75 (0.73, 0.78)
	Most deprived	0.67 (0.65, 0.70)	0.72 (0.70, 0.75)	0.67 (0.65, 0.69)	0.72 (0.69, 0.74)	0.69 (0.67, 0.71)	0.76 (0.73, 0.78)	0.67 (0.65, 0.69)	0.71 (0.69, 0.74)
Region	London	1	1	1	1	1	1	1	1
	North East	1.11 (1.02, 1.21)	1.21 (1.11, 1.31)	1.14 (1.06, 1.24)	1.24 (1.14, 1.34)	1.15 (1.07, 1.24)	1.25 (1.16, 1.35)	1.09 (1.01, 1.18)	1.19 (1.09, 1.29)
	North West	1.37 (1.32, 1.42)	1.42 (1.36, 1.47)	1.39 (1.34, 1.45)	1.44 (1.39, 1.50)	1.42 (1.38, 1.47)	1.48 (1.42, 1.53)	1.38 (1.33, 1.44)	1.44 (1.38, 1.50)
	Yorkshire & The Humber	1.27 (1.18, 1.38)	1.26 (1.16, 1.37)	1.32 (1.22, 1.43)	1.31 (1.21, 1.42)	1.33 (1.24, 1.43)	1.33 (1.23, 1.43)	1.27 (1.17, 1.38)	1.26 (1.17, 1.37)
	East Midlands	1.33 (1.21, 1.47)	1.40 (1.27, 1.55)	1.34 (1.22, 1.47)	1.41 (1.29, 1.55)	1.33 (1.23, 1.45)	1.39 (1.28, 1.52)	1.35 (1.23, 1.49)	1.43 (1.30, 1.58)
	West Midlands	1.41 (1.35, 1.46)	1.43 (1.37, 1.49)	1.42 (1.37, 1.48)	1.45 (1.39, 1.51)	1.43 (1.38, 1.48)	1.45 (1.40, 1.51)	1.47 (1.41, 1.53)	1.50 (1.44, 1.57)
	East of England	1.31 (1.26, 1.37)	1.24 (1.19, 1.30)	1.31 (1.26, 1.37)	1.24 (1.19, 1.30)	1.31 (1.26, 1.36)	1.24 (1.19, 1.29)	1.32 (1.26, 1.37)	1.25 (1.20, 1.31)
	South West	1.25 (1.20, 1.31)	1.25 (1.20, 1.31)	1.29 (1.24, 1.35)	1.29 (1.24, 1.35)	1.34 (1.29, 1.39)	1.34 (1.29, 1.39)	1.27 (1.22, 1.32)	1.27 (1.22, 1.33)
	South Central	1.42 (1.36, 1.47)	1.33 (1.28, 1.38)	1.45 (1.40, 1.50)	1.36 (1.31, 1.41)	1.47 (1.42, 1.52)	1.38 (1.33, 1.43)	1.43 (1.38, 1.48)	1.34 (1.29, 1.40)
	South East Coast	1.06 (1.02, 1.10)	1.02 (0.98, 1.06)	1.07 (1.04, 1.11)	1.03 (0.99, 1.07)	1.11 (1.07, 1.15)	1.07 (1.03, 1.11)	1.05 (1.01, 1.09)	1.01 (0.97, 1.05)
Ethnicity	White	1	1	1	1	1	1	1	1
	South Asian	0.92 (0.88, 0.95)	0.99 (0.95, 1.03)	0.92 (0.88, 0.95)	0.99 (0.95, 1.03)	0.93 (0.90, 0.96)	0.98 (0.95, 1.02)	0.94 (0.91, 0.98)	1.02 (0.98, 1.06)
	Black	0.67 (0.64, 0.71)	0.83 (0.78, 0.88)	0.68 (0.64, 0.71)	0.83 (0.78, 0.88)	0.69 (0.65, 0.72)	0.83 (0.79, 0.87)	0.67 (0.64, 0.71)	0.83 (0.79, 0.88)
	Mixed	0.84 (0.77, 0.92)	0.91 (0.83, 0.99)	0.78 (0.72, 0.85)	0.84 (0.77, 0.92)	0.79 (0.73, 0.86)	0.86 (0.79, 0.93)	0.83 (0.76, 0.91)	0.90 (0.82, 0.99)
	Other	0.79 (0.73, 0.84)	0.85 (0.79, 0.91)	0.75 (0.70, 0.80)	0.81 (0.76, 0.86)	0.77 (0.73, 0.82)	0.83 (0.78, 0.88)	0.80 (0.75, 0.85)	0.86 (0.80, 0.92)
Maternal age, years	<20	0.87 (0.81, 0.92)	0.87 (0.82, 0.93)	0.87 (0.82, 0.93)	0.87 (0.82, 0.93)	0.68 (0.65, 0.72)	0.68 (0.64, 0.71)	0.88 (0.82, 0.93)	0.88 (0.83, 0.94)
	20-24	1	1	1	1	1	1	1	1
	25-29	1.13 (1.09, 1.17)	1.12 (1.08, 1.16)	1.14 (1.10, 1.18)	1.13 (1.10, 1.17)	1.24 (1.20, 1.28)	1.25 (1.21, 1.29)	1.13 (1.09, 1.17)	1.13 (1.09, 1.17)
	30-35	1.22 (1.18, 1.26)	1.21 (1.17, 1.25)	1.24 (1.20, 1.28)	1.23 (1.19, 1.27)	1.36 (1.32, 1.41)	1.38 (1.34, 1.42)	1.23 (1.19, 1.27)	1.22 (1.17, 1.26)
	≥35	1.17 (1.12, 1.21)	1.18 (1.13, 1.22)	1.19 (1.15, 1.23)	1.20 (1.15, 1.24)	1.18 (1.14, 1.21)	1.21 (1.17, 1.25)	1.16 (1.12, 1.21)	1.18 (1.14, 1.23)
	0	1	1	1	1	1	1	1	1

For peer review only - http://bmjopen.bmj.com/site/about/guidelines.xhtml

Number of children	1	0.80 (0.78, 0.82)	0.80 (0.78, 0.82)	0.84 (0.82, 0.86)	0.83 (0.81, 0.85)	0.87 (0.85, 0.89)	0.86 (0.84, 0.88)	0.79 (0.77, 0.80)	0.78 (0.76, 0.80)
	2	0.71 (0.68, 0.73)	0.71 (0.69, 0.74)	0.75 (0.72, 0.77)	0.74 (0.72, 0.77)	0.72 (0.70, 0.75)	0.71 (0.68, 0.73)	0.69 (0.67, 0.71)	0.69 (0.67, 0.72)
	≥3	0.61 (0.58, 0.63)	0.62 (0.59, 0.65)	0.64 (0.61, 0.67)	0.65 (0.62, 0.68)	0.63 (0.61, 0.66)	0.63 (0.60, 0.66)	0.59 (0.56, 0.61)	0.60 (0.57, 0.63)
Clinical risk group	No	1	1	1	1	1	1	1	1
	Yes	1.69 (1.62, 1.76)	1.70 (1.63, 1.77)	1.73 (1.66, 1.80)	1.73 (1.66, 1.80)	1.98 (1.91, 2.06)	2.00 (1.93, 2.07)	1.59 (1.53, 1.66)	1.60 (1.54, 1.67)
BMI	<18.5	0.85 (0.80, 0.90)		0.84 (0.79, 0.89)		0.84 (0.79, 0.88)		0.93 (0.88, 0.98)	
	18.5-24.9	1		1		1		1	
	25.0-29.9	1.04 (1.01, 1.07)		1.03 (1.01, 1.06)		1.04 (1.02, 1.07)		0.98 (0.95, 1.00)	
	≥30	1.00 (0.97, 1.03)		0.99 (0.96, 1.02)		1.03 (1.00, 1.06)		0.90 (0.87, 0.92)	

Note: All models include women who registered in first trimester, and exclude those with outcome unknown and missing ethnicity; minimally adjusted model of BMI excludes women with missing BMI

Abbreviations: UK, United Kingdom; IMD, Index of Multiple Deprivation; BMI, body mass index

For peer review only

Supplementary Table 7: Secondary analysis of subsequent pertussis vaccine uptake among women who had received pertussis vaccination in their first eligible pregnancy and had a second eligible pregnancy within the study period (N=3,111)

		Total (column %)	Received pertussis vaccine in second pregnancy (row %)	Minimally adjusted model OR of receiving vaccine in second pregnancy (95% CI)	Fully adjusted model OR of receiving vaccine in second pregnancy (95% CI)
N		3,111	1,877 (60.3)		
Year of first pregnancy	2012	550 (17.7)	380 (69.1)	1	1
	2013	1,912 (61.5)	1,264 (66.1)	0.87 (0.71-1.07)	0.70 (0.56-0.87)
	2014-15	649 (20.9)	233 (35.9)	0.25 (0.20-0.32)	0.14 (0.10-0.18)
Index of multiple deprivation (IMD) quintile	Least deprived	857 (27.6)	539 (62.9)	1	1
	2	539 (17.3)	326 (60.5)	0.90 (0.71-1.13)	0.91 (0.71-1.16)
	3	604 (19.4)	381 (63.1)	1.03 (0.92-1.28)	1.06 (0.83-1.35)
	4	579 (18.6)	337 (58.2)	0.82(0.66-1.02)	0.89 (0.70-1.15)
	Most deprived	532 (17.1)	294 (55.3)	0.72 (0.57-0.90)	0.77 (0.59-1.01)
Region	London	453 (14.6)	260 (57.4)	1	1
	North East	35 (1.1)	22 (62.9)	1.25 (0.65-2.83)	2.08 (0.95-4.58)
	North West	390 (12.5)	240 (61.5)	1.16 (0.87-1.55)	1.29 (0.95-1.77)
	Yorkshire & The Humber	31 (1.0)	14 (45.2)	0.56 (0.27-1.19)	0.73 (0.33-1.62)
	East Midlands	0	0	-	-
	West Midlands	375 (12.1)	229 (61.1)	1.13 (0.85-1.51)	1.33 (0.97-1.81)
	East of England	296 (9.5)	201 (67.9)	1.57 (1.14-2.15)	1.54 (1.10-2.16)
	South West	388 (12.5)	239 (61.6)	1.19 (0.98-1.58)	1.31 (0.96-1.79)
	South Central	562 (18.1)	360 (64.1)	1.33 (1.02-1.73)	1.31 (0.99-1.74)
	South East Coast	581 (18.7)	312 (53.7)	0.90 (0.69-1.16)	0.99 (0.75-1.31)
Ethnicity	White	2,732 (87.8)	1,657 (60.7)	1	1
	South Asian	204 (6.6)	114 (55.9)	0.82 (0.61-1.10)	0.78 (0.57-1.07)
	Black	84 (2.7)	49 (58.3)	1.05 (0.66-1.67)	1.09 (0.66-1.80)
	Mixed	33 (1.1)	20 (60.6)	0.94 (0.46-1.94)	1.14 (0.63-2.07)
	Other	58 (1.9)	37 (63.8)	1.25 (0.71-2.20)	0.97 (0.46-2.06)
Maternal age, years	<20	102 (3.2)	40 (39.2)	0.48 (0.30-0.75)	0.48 (0.30-0.77)
	20-24	505 (16.2)	290 (57.4)	1	1
	25-29	1,002 (32.2)	592 (59.1)	1.07 (0.85-1.34)	1.09 (0.86-1.39)
	30-34	1,048 (33.7)	669 (63.8)	1.32 (1.05-1.65)	1.26 (0.98-1.61)
	≥35	454 (14.6)	286 (63.0)	1.29 (0.99-1.69)	1.26 (0.94-1.69)
Number of children	0	1,936 (62.2)	1,224 (63.2)	1	1
	1	714 (23.0)	405 (56.7)	0.78 (0.65-0.94)	0.75 (0.62-0.91)
	2	264 (8.5)	149 (56.4)	0.72 (0.55-0.95)	0.64 (0.48-0.85)
	≥3	197 (6.3)	99 (50.3)	0.56 (0.42-0.76)	0.50 (0.36-0.69)
Pregnancy interval (days from end of first pregnancy to start of second)	0-179	416 (13.4)	227 (54.6)	1	1
	180-359	749 (24.1)	476 (63.6)	1.25 (0.96-1.63)	1.11 (0.85-1.45)
	360-539	1,004 (32.3)	695 (69.2)	1.33 (1.03-1.71)	1.13 (0.86-1.47)
	540-719	624 (20.1)	373 (59.8)	0.65 (0.49-0.85)	0.54 (0.41-0.73)
	720+	318 (10.2)	106 (33.3)	0.19 (0.14-0.27)	0.16 (0.11-0.22)

Note: Among 3,363 women with two eligible pregnancies during follow up, excluded 2 with implausible (<0 days) spacing between the end of the first pregnancy and start of the second, and 250 with missing ethnicity data.

For peer review only

The RECORD statement – checklist of items, extended from the STROBE statement, that should be reported in observational studies using routinely collected health data.

	Item No.	STROBE items	Location in manuscript where items are reported	RECORD items	Location in manuscript where items are reported
**Title and abstract**					
	1	(a) Indicate the study's design with a commonly used term in the title or the abstract (b) Provide in the abstract an informative and balanced summary of what was done and what was found	Title and abstract	RECORD 1.1: The type of data used should be specified in the title or abstract. When possible, the name of the databases used should be included.<br><br>RECORD 1.2: If applicable, the geographic region and timeframe within which the study took place should be reported in the title or abstract.<br><br>RECORD 1.3: If linkage between databases was conducted for the study, this should be clearly stated in the title or abstract.	Title and abstract<br><br>Abstract<br><br>No new linkage conducted for the study (use of pre-linked data described in methods)
**Introduction**					
Background rationale	2	Explain the scientific background and rationale for the investigation being reported	Introduction pages 5-6		
Objectives	3	State specific objectives, including any prespecified hypotheses	Introduction page 6		
**Methods**					
Study Design	4	Present key elements of study design early in the paper	Abstract and methods page 7		
Setting	5	Describe the setting, locations, and relevant dates, including	Abstract and methods page 7		

For peer review only

		periods of recruitment, exposure, follow-up, and data collection			
Participants	6	*(a) Cohort study* - Give the eligibility criteria, and the sources and methods of selection of participants. Describe methods of follow-up<br>*Case-control study* - Give the eligibility criteria, and the sources and methods of case ascertainment and control selection. Give the rationale for the choice of cases and controls<br>*Cross-sectional study* - Give the eligibility criteria, and the sources and methods of selection of participants<br><br>*(b) Cohort study* - For matched studies, give matching criteria and number of exposed and unexposed<br>*Case-control study* - For matched studies, give matching criteria and the number of controls per case	Cohort – methods pages 7-8<br><br>N/A Cohort – no matching	RECORD 6.1: The methods of study population selection (such as codes or algorithms used to identify subjects) should be listed in detail. If this is not possible, an explanation should be provided.<br><br>RECORD 6.2: Any validation studies of the codes or algorithms used to select the population should be referenced. If validation was conducted for this study and not published elsewhere, detailed methods and results should be provided.<br><br>RECORD 6.3: If the study involved linkage of databases, consider use of a flow diagram or other graphical display to demonstrate the data linkage process, including the number of individuals with linked data at each stage.	Cohort – methods pages 7-8<br><br>N/A<br><br>No new data linkages
Variables	7	Clearly define all outcomes, exposures, predictors, potential confounders, and effect modifiers. Give diagnostic criteria, if applicable.	Methods pages 9-10	RECORD 7.1: A complete list of codes and algorithms used to classify exposures, outcomes, confounders, and effect modifiers should be provided. If these cannot be reported, an explanation should be provided.	Methods pages 9-10
Data sources/ measurement	8	For each variable of interest, give sources of data and details of methods of assessment (measurement).	Methods pages 9-10		

For peer review only

		Describe comparability of assessment methods if there is more than one group			
Bias	9	Describe any efforts to address potential sources of bias	Methods page 11-12		
Study size	10	Explain how the study size was arrived at	Methods page 6-8		
Quantitative variables	11	Explain how quantitative variables were handled in the analyses. If applicable, describe which groupings were chosen, and why	Methods page 10		
Statistical methods	12	(a) Describe all statistical methods, including those used to control for confounding<br>(b) Describe any methods used to examine subgroups and interactions<br>(c) Explain how missing data were addressed<br>(d) *Cohort study* - If applicable, explain how loss to follow-up was addressed<br>*Case-control study* - If applicable, explain how matching of cases and controls was addressed<br>*Cross-sectional study* - If applicable, describe analytical methods taking account of sampling strategy<br>(e) Describe any sensitivity analyses	Methods page 10-12<br>N/A<br>Methods page 12<br>Methods page 9<br>Methods page 12		
Data access and cleaning methods		..		RECORD 12.1: Authors should describe the extent to which the investigators had access to the database population used to create the study population.	Page 22 author contributions

For peer review only

				RECORD 12.2: Authors should provide information on the data cleaning methods used in the study.	Methods pages 9-10, and results page 14
Linkage		..		RECORD 12.3: State whether the study included person-level, institutional-level, or other data linkage across two or more databases. The methods of linkage and methods of linkage quality evaluation should be provided.	No data linkage – this study used pre-linked data only, as described in Methods page 9
**Results**					
Participants	13	(a) Report the numbers of individuals at each stage of the study (*e.g.*, numbers potentially eligible, examined for eligibility, confirmed eligible, included in the study, completing follow-up, and analysed)<br>(b) Give reasons for non-participation at each stage.<br>(c) Consider use of a flow diagram	Methods page 9, results page 14	RECORD 13.1: Describe in detail the selection of the persons included in the study (*i.e.,* study population selection) including filtering based on data quality, data availability and linkage. The selection of included persons can be described in the text and/or by means of the study flow diagram.	Methods page 9, results page 14
Descriptive data	14	(a) Give characteristics of study participants (*e.g.*, demographic, clinical, social) and information on exposures and potential confounders<br>(b) Indicate the number of participants with missing data for each variable of interest<br>(c) *Cohort study* - summarise follow-up time (*e.g.*, average and total amount)	Results page 14, Tables 1 and 2<br>Results page 14 and supplementary table 2<br>N/A		
Outcome data	15	*Cohort study* - Report numbers of outcome events or summary measures over time	Tables 1 and 2		

For peer review only

		Case-control study - Report numbers in each exposure category, or summary measures of exposure<br>*Cross-sectional study* - Report numbers of outcome events or summary measures			
Main results	16	(a) Give unadjusted estimates and, if applicable, confounder-adjusted estimates and their precision (e.g., 95% confidence interval). Make clear which confounders were adjusted for and why they were included<br>(b) Report category boundaries when continuous variables were categorized<br>(c) If relevant, consider translating estimates of relative risk into absolute risk for a meaningful time period	Tables 1 and 2<br><br>Tables 1 and 2<br><br>N/A		
Other analyses	17	Report other analyses done—e.g., analyses of subgroups and interactions, and sensitivity analyses	Methods page 12-13, supplementary tables 3-7		
**Discussion**					
Key results	18	Summarise key results with reference to study objectives	Discussion page 19		
Limitations	19	Discuss limitations of the study, taking into account sources of potential bias or imprecision. Discuss both direction and magnitude of any potential bias	Discussion page 19	RECORD 19.1: Discuss the implications of using data that were not created or collected to answer the specific research question(s). Include discussion of misclassification bias, unmeasured confounding, missing data, and changing eligibility over time, as they pertain to the study being reported.	Discussion page 19

For peer review only

Interpretation	20	Give a cautious overall interpretation of results considering objectives, limitations, multiplicity of analyses, results from similar studies, and other relevant evidence	Discussion pages 20-21		
Generalisability	21	Discuss the generalisability (external validity) of the study results	Discussion page 19 re other settings		
**Other Information**					
Funding	22	Give the source of funding and the role of the funders for the present study and, if applicable, for the original study on which the present article is based	Funding statement		
Accessibility of protocol, raw data, and programming code		..		RECORD 22.1: Authors should provide information on how to access any supplemental information such as the study protocol, raw data, or programming code.	Methods page 10

*Reference: Benchimol EI, Smeeth L, Guttmann A, Harron K, Moher D, Petersen I, Sørensen HT, von Elm E, Langan SM, the RECORD Working Committee. The REporting of studies Conducted using Observational Routinely-collected health Data (RECORD) Statement. *PLoS Medicine* 2015; in press.

*Checklist is protected under Creative Commons Attribution (CC BY) license.

For peer review only

BMJ Open

Social determinants of pertussis and influenza vaccine uptake in pregnancy: a national cohort study in England using electronic health records

Journal:	*BMJ Open*
Manuscript ID	bmjopen-2020-046545.R2
Article Type:	Original research
Date Submitted by the Author:	07-May-2021
Complete List of Authors:	Walker, Jemma; London School of Hygiene and Tropical Medicine, Faculty of Epidemiology and Population Health; Public Health England, Statistics, Modelling and Economics Department<br>Rentsch, Christopher ; London School of Hygiene & Tropical Medicine, Faculty of Epidemiology and Population Health<br>McDonald, Helen; London School of Hygiene and Tropical Medicine, Faculty of Epidemiology and Population Health<br>Bak, JeongEun; London School of Hygiene and Tropical Medicine, Faculty of Epidemiology and Population Health<br>Minassian, Caroline ; London School of Hygiene & Tropical Medicine, Faculty of Epidemiology and Population Health<br>Amirthalingam, Gayatri; Public Health England Immunisation and Countermeasures Division<br>Edelstein, Michael; Public Health England Immunisation and Countermeasures Division; London School of Hygiene & Tropical Medicine, Faculty of Epidemiology and Population Health<br>Thomas, Sara; London School of Hygiene & Tropical Medicine, Faculty of Epidemiology & Population Health
**Primary Subject Heading**:	Public health
Secondary Subject Heading:	Epidemiology, Health services research, Obstetrics and gynaecology
Keywords:	Public health < INFECTIOUS DISEASES, Maternal medicine < OBSTETRICS, PRIMARY CARE, PUBLIC HEALTH

SCHOLARONE™
Manuscripts

I, the Submitting Author has the right to grant and does grant on behalf of all authors of the Work (as defined in the below author licence), an exclusive licence and/or a non-exclusive licence for contributions from authors who are: i) UK Crown employees; ii) where BMJ has agreed a CC-BY licence shall apply, and/or iii) in accordance with the terms applicable for US Federal Government officers or employees acting as part of their official duties; on a worldwide, perpetual, irrevocable, royalty-free basis to BMJ Publishing Group Ltd ("BMJ") its licensees and where the relevant Journal is co-owned by BMJ to the co-owners of the Journal, to publish the Work in this journal and any other BMJ products and to exploit all rights, as set out in our licence.

The Submitting Author accepts and understands that any supply made under these terms is made by BMJ to the Submitting Author unless you are acting as an employee on behalf of your employer or a postgraduate student of an affiliated institution which is paying any applicable article publishing charge ("APC") for Open Access articles. Where the Submitting Author wishes to make the Work available on an Open Access basis (and intends to pay the relevant APC), the terms of reuse of such Open Access shall be governed by a Creative Commons licence – details of these licences and which Creative Commons licence will apply to this Work are set out in our licence referred to above.

Other than as permitted in any relevant BMJ Author's Self Archiving Policies, I confirm this Work has not been accepted for publication elsewhere, is not being considered for publication elsewhere and does not duplicate material already published. I confirm all authors consent to publication of this Work and authorise the granting of this licence.

For peer review only

Social determinants of pertussis and influenza vaccine uptake in pregnancy: a national cohort study in England using electronic health records

Authors: Jemma L Walker (0000-0003-3728-9509),[1,2,3*] Christopher T Rentsch (0000-0002-1408-7907),[1,2*] Helen I McDonald (0000-0003-0576-2015),[1,2§] JeongEun Bak (0000-0001-7519-7539),[2] Caroline Minassian (0000-0001-9406-1928),[2] Gayatri Amirthalingam (0000-0003-2078-0975),[1,4] Michael Edelstein (0000-0002-7323-0806),[1,2,4] Sara L Thomas.[1,2]

*Joint first authorship

1. NIHR Health Protection Research Unit in Immunisation
2. Faculty of Epidemiology and Population Health, London School of Hygiene and Tropical Medicine, London, WC1E 7HT, UK
    a. Jemma L Walker research fellow, Christopher T Rentsch research fellow, Helen I McDonald clinical research fellow, JeongEun Bak MSc student, Caroline Minassian assistant professor, Michael Edelstein honorary associate professor, Sara L Thomas professor
3. Statistics, Modelling and Economics Department, Public Health England, London, NW9 5EQ, UK
    a. Jemma L Walker statistician
4. Immunisation and Countermeasures Division, National Infection Service, Public Health England, London, NW9 5EQ, UK
    a. Gayatri Amirthalingam consultant epidemiologist, Michael Edelstein consultant epidemiologist

§Corresponding author:
Helen I McDonald, PhD
London School of Hygiene & Tropical Medicine
Keppel Street
WC1E 7HT
London, UK
Email: helen.mcdonald@lshtm.ac.uk

Article type: Research
Keywords: Influenza vaccine; pertussis vaccine; vaccination coverage; social determinants of health; electronic health records.

Word count: 4,255

For peer review only

ABSTRACT

Objective To examine the social determinants of influenza and pertussis vaccine uptake among pregnant women in England.

Design Nationwide population-based cohort study

Setting The study used anonymised primary care data from the Clinical Practice Research Datalink and linked Hospital Episode Statistics secondary care data

Participants Pregnant women eligible for pertussis (2012 to 2015, n=68,090) or influenza (2010/11 to 2015/16, n=152,132) vaccination in England

Main outcome measures Influenza and pertussis vaccine uptake

Results

Vaccine uptake was 67.3% for pertussis, and 39.1% for influenza. Uptake of both vaccines varied by region, with lowest uptakes in London and the North East. Lower vaccine uptake was associated with greater deprivation: almost 10% lower in the most deprived quintiles compared with the least deprived for influenza (34.5% vs 44.0%), and almost 20% lower for pertussis (57.7% vs 76.0%). Lower uptake for both vaccines was also associated with non-white ethnicity (lowest among women of Black ethnicity), maternal age under 20 years, and a greater number of children in the household. The associations between all social factors and vaccine uptake were broadly unchanged in fully adjusted models, suggesting the social determinants of uptake were largely independent of one another.

Among 3,111 women vaccinated against pertussis in their first eligible pregnancy and pregnant again, 1,234 (40%) were not vaccinated in their second eligible pregnancy.

For peer review only - http://bmjopen.bmj.com/site/about/guidelines.xhtml

Conclusions

Targeting promotional campaigns to pregnant women who are younger, of non-white ethnicity, with more children, living in areas of greater deprivation or the London or North East regions, has potential to reduce vaccine-preventable disease among infants and pregnant women, and to reduce health inequalities. Vaccination promotion needs to be sustained across successive pregnancies. Further research is needed into whether the effectiveness of vaccine promotion strategies may vary according to social factors.

For peer review only

Article Summary

Strengths and limitations of this study

- This large cohort study explored the social determinants of influenza and pertussis vaccination among pregnant women across England. It considered a range of social determinants including maternal age, ethnicity, socio-economic status, number of children in the household, and region.
- The CPRD/LSHTM pregnancy register was used to ascertain pregnancies and their timing from primary care records using detailed algorithms.
- We were unable to investigate other potential social determinants of uptake not routinely recorded in primary care records such as education or religion.

For peer review only

For peer review only - http://bmjopen.bmj.com/site/about/guidelines.xhtml

INTRODUCTION

Pertussis (whooping cough) and seasonal influenza are vaccine-preventable diseases. Influenza can have severe outcomes among pregnant women and young infants, including hospitalisation and death.[1] Pertussis can be a serious illness for young infants: a pertussis outbreak in 2012 resulted in 14 infant deaths, most of whom were too young to be vaccinated directly.[2-4] Vaccination in pregnancy reduces influenza-associated hospitalisation among pregnant women,[5] and provides 'passive immunity' to protect infants in the first months of life.[6, 7] In England, pertussis vaccination has been offered to women in later stages of pregnancy since 2012 and seasonal influenza vaccination at any stage of pregnancy during influenza season since 2010, with both provided free of charge.[2, 8]

Low vaccine uptake during pregnancy is a major public health challenge for high-income countries.[9] According to routine surveillance in 2018/19, vaccine uptake amongst pregnant women in England was 68.8% for pertussis and 45.2% for influenza.[10, 11] Although comparatively high for a high-income country, this suboptimal uptake still limits the programme's impact and results in vaccine-preventable deaths among infants of unvaccinated mothers. Studies of determinants of maternal influenza vaccine uptake to date have largely focused on health beliefs.[12] Studies in the United States have found inequalities in vaccine uptake during pregnancy by ethnicity/race, age and insurance status.[13-15] Less is known about the role of social factors in England. During the 2009 influenza pandemic, higher vaccine uptake in pregnancy was associated with higher maternal age, previous deliveries, and underlying health conditions but not deprivation.[16] However, ecological studies suggest that both seasonal influenza and pertussis vaccine uptake in pregnancy vary with ethnicity, and are lower in areas with greater deprivation, and are thus sources

For peer review only - http://bmjopen.bmj.com/site/about/guidelines.xhtml

of health inequalities in infancy.[17, 18] Smaller studies of pertussis and seasonal influenza vaccines have suggested deprivation, ethnicity, maternal age and parity or number of children may be factors in maternal vaccine uptake, but have lacked power to describe these associations fully.[19-23] A better understanding of the social determinants of maternal vaccine uptake could inform targeted public health interventions to improve vaccine uptake and reduce health inequalities.

This study aimed to use linked electronic health records to examine the social determinants of influenza and pertussis vaccine uptake among pregnant women in England for the first few years from programme introduction: 2012 to 2015 for pertussis and 2010/11 to 2015/16 for influenza vaccination.

For peer review only

For peer review only - http://bmjopen.bmj.com/site/about/guidelines.xhtml

METHODS

Data sources

This historical cohort study used data from the Clinical Practice Research Datalink (CPRD), a quality-assured anonymised primary care patient dataset covering approximately 7% of general practices in England, a representative sample of the population by age and sex.[24, 25] Available data include diagnoses and symptoms, prescriptions, immunisations and referrals recorded in primary care. The CPRD/LSHTM Pregnancy Register details all pregnancies recorded in primary care, identified using detailed algorithms to determine their timing and outcomes.[26] The Pregnancy Register has been found to have a high sensitivity for livebirths (including 90% of all deliveries recorded in secondary care) but may under-record pregnancies which end in a loss.[26, 27] For this analysis, we used the Pregnancy Register and CPRD data pre-linked to Hospital Episode Statistics (HES) admissions data (for supplementary ethnicity data),[28] and Office of National Statistics (ONS) small-area-level deprivation data.[29]

Study population

Analysis of pertussis vaccine and seasonal influenza vaccine uptake were conducted separately. For each vaccine, we identified pregnancies eligible for the relevant vaccination among women registered with CPRD, using the Pregnancy Register to identify start and end dates of pregnancies, eligible dates based on gestation, and pregnancy outcomes. Eligible women were registered at one of the 75% of CPRD practices in England which participate in the CPRD data-linkage scheme, for availability of linked HES and ONS data.[24] Vaccine eligibility started on or after 1

For peer review only - http://bmjopen.bmj.com/site/about/guidelines.xhtml

October 2012 for the pertussis vaccine analyses, and on or after 1 April 2010 for the seasonal influenza vaccine analyses, reflecting the introduction of vaccination programmes.[2, 8] For each vaccine, the first eligible pregnancy for each woman during the follow-up period was used to avoid non-independence in the data.

Vaccination guidelines during the study period suggested women should be offered pertussis vaccination in their third trimester of pregnancy (ideally between 28-32 weeks, though it could be offered between 28-38 weeks' gestation).[2] For the pertussis vaccine analyses, we included women who delivered a live-or stillborn child on or after 26 weeks of pregnancy and followed up for vaccination up to 40 weeks' gestation, which allowed for up to 2 weeks imprecision in the Pregnancy Register estimation of the vaccine eligible period and mirrored the national surveillance approach. The study period ended before the April 2016 change in guidelines recommending vaccination at 16-32 weeks of pregnancy (though it may be given up to delivery), and changes in the commissioning arrangements leading to increased delivery through maternity services from 2016.[2]

Influenza vaccination is recommended at any stage in pregnancy that overlaps with the influenza season.[8] For the influenza vaccine analyses, all pregnancies for which the Pregnancy Register included a known outcome (such as stillbirth, livebirth, miscarriage, or termination) were included, irrespective of duration of pregnancy, providing the pregnancy overlapped by at least one day with the influenza season (1 September to 31 January of each year).

We limited primary analyses for both maternal vaccines to women who registered as patients at the primary care practice by the end of their first trimester, to reduce

For peer review only - http://bmjopen.bmj.com/site/about/guidelines.xhtml

misclassification of vaccination status. We conducted sensitivity analyses around the study inclusion criteria, which are described below.

Follow-up period

The study period ranged from 1 October 2012 to 30 September 2015 for pertussis vaccine and 1 September 2010 to 31 January 2016 for influenza vaccine. Start of follow-up was considered the latest date of: start of the study period, practice meeting CPRD quality standards, patient registration at the practice, 11th birthday (dates of birth based on the mid-point of year of birth), 26 weeks gestation of pregnancy (for pertussis), the start of pregnancy plus 2 weeks (for influenza), or 1st September of each year (for influenza). End of follow-up was the earliest date of: last data collection from the practice, end of linkage to HES, patient transfer out of the practice, 49th birthday, death, receipt of the vaccine of interest, the 40th week of pregnancy (for pertussis), end of pregnancy (for influenza), end of the study period, or 31 January of each year (for influenza).

Vaccine uptake

Vaccination status for both maternal pertussis and influenza vaccines was extracted from CPRD. For the primary analysis of pertussis vaccine uptake, women were considered vaccinated if they received the vaccine between 26 and 40 weeks of pregnancy gestation, which is similar to the national vaccination guidelines of 28 to 38 weeks but allows for up to two weeks discrepancy in the Pregnancy Register estimation of gestation. Women who were not vaccinated between 26 and 40 weeks of gestation were considered unvaccinated, irrespective of vaccination before 26 weeks or after 40 weeks of gestation. For the primary analysis of influenza vaccine

For peer review only - http://bmjopen.bmj.com/site/about/guidelines.xhtml

uptake, women were considered vaccinated if they received the vaccine on any day between 1 September and 31 January during their follow-up period. Women with a pregnancy that spanned two influenza seasons (n=19,963, 14%) were counted in the denominator of the latter season and considered vaccinated if vaccinated in either season.

Social characteristics and clinical conditions

We defined social determinants using previously published detailed algorithms.[30] Index of multiple deprivation (IMD, a composite measure of relative deprivation) was assigned in quintiles (1 representing least deprived, 5 most deprived) based on the Lower Super Output Area of the patient's residential address using ONS national statistics data.[29] Ethnicity (White, South Asian, Black, Mixed, Other) was defined using primary care records supplemented with linked HES data.[28] Other social factors of interest were defined using CPRD primary care data and comprised: region of residence (London, North East, North West, Yorkshire & The Humber, East Midlands, West Midlands, East of England, South West, South Central, and South East Coast), maternal age (based on midpoint of year of birth), and number of children in the household.

For influenza vaccine uptake analyses, whether the individual was in a clinical risk group indicated to receive influenza vaccine was defined according to national guidance,[8] and comprised the following conditions: chronic renal disease, chronic heart disease, chronic respiratory disease, chronic liver disease, diabetes, immunosuppression, chronic neurological disease, asplenia, and morbid obesity. Clinical risk groups were identified using Read codes, primary care prescription

For peer review only - http://bmjopen.bmj.com/site/about/guidelines.xhtml

records (for immunosuppression and asthma), and height and weight records. Body mass index (BMI) was defined using height and weight records using validated methods,[31] and defined based on the record closest to the beginning of pregnancy, allowing measures during the first trimester of pregnancy. Asthma was defined as an asthma diagnosis and either any history of an emergency hospital admission for asthma, or any inhaled or oral steroid prescription in the previous 12 months. The algorithms used for immunosuppression are described in previous studies;[32] codelists for other conditions are available from https://doi.org/10.17037/DATA.00001907.

Statistical analysis

Parallel analyses were conducted for pertussis and influenza vaccine uptake. For each vaccine, a complete case analysis (excluding women with no ethnicity recorded in the main analysis) using multivariable logistic regression was used to estimate associations between vaccine uptake and social determinants. Our modelling strategy followed a previously adapted version[33] of a conceptual framework to analyse the hierarchical inter-relationships between distal and proximate social determinants with vaccine uptake **(Supplementary Table 1)**.[34] We first fitted a 'minimally adjusted' model to estimate associations between each social determinant and vaccine uptake adjusted for year (calendar year for pertussis, financial year for influenza to reflect the influenza season) to adjust for secular trends as an *a priori* confounder. We then fitted five further sequential models. Models 1 to 3 explored the social determinants of uptake from distal to proximal. Model 4 and the BMI Model explored the extent to which these were mediated by clinical conditions (for influenza), and mediated and/or confounded by BMI (for both vaccines).

For peer review only - http://bmjopen.bmj.com/site/about/guidelines.xhtml

In Model 1 we assessed associations between vaccine uptake and the distal determinants IMD, region, and ethnicity, mutually adjusted and adjusted for year. In Model 2 the intermediate variable maternal age was added alongside the variables in Model 1 to determine to what extent this explained any effect of the distal variables. Model 3 comprised the variables in Model 2 and the proximate variable number of children, to investigate whether this mediated the effect of the distal and intermediate variables. For influenza uptake modelling, we further added clinical risk group as a potential mediator of the social characteristics (Model 4). Finally, we repeated complete case analyses additionally excluding women with no recorded BMI for all four models, adding a further model (BMI Model) that additionally adjusted for BMI, which may both mediate and confound the effect of social characteristics and clinical conditions.

All analyses were conducted using Stata 15 (StataCorp, College Station, TX, USA).

Missing data and sensitivity analyses

Primary analyses were conducted on women who had non-missing ethnicity and who were registered with an up-to-standard CPRD practice by the end of their first trimester. Other than ethnicity, only BMI had missing data.

We performed descriptive and sensitivity analyses to understand how estimates of vaccine uptake and associations with social determinants might be affected by missing data or study inclusion criteria. First, we examined the distribution of social determinants among women with and without recorded ethnicity. Second, we compared estimates from minimally and fully adjusted models from the primary analyses with sensitivity analyses including women who registered with an up-to-standard practice by the end of pregnancy (instead of end of first trimester) for both

For peer review only - http://bmjopen.bmj.com/site/about/guidelines.xhtml

vaccines. For the pertussis analyses, we further ran minimally and fully adjusted models that mirrored national surveillance criteria of immunisation at 28-38 weeks' gestation, to assess the impact of allowing a two-week window for imprecise estimation of gestation in our primary analysis. For the influenza analyses, we further ran models that included pregnancies with no recorded outcome, as well as models that extended the influenza season through 31 March of each year. Finally, for both pertussis and influenza analyses, we fitted random effects models to test for clustering by general practice.

Secondary analysis of sequential pregnancies

In response to the finding that vaccine uptake declined with greater number of children in the household, a *post-hoc* secondary analysis was added investigating the social determinants associated with vaccination in a second eligible pregnancy among women who had received pertussis vaccination in their first eligible pregnancy. This analysis focused on pertussis vaccination, as influenza vaccination uptake may depend upon the extent and timing of the overlap of pregnancy with the influenza season, severity of the influenza season and timing of vaccine availability, reducing the number of eligible sequential pregnancies and increasing the complexity of external factors which may affect a women's vaccine uptake across sequential pregnancies. Logistic regression with likelihood ratio tests were used to model and test minimally adjusted and fully adjusted (Model 3) associations between the outcome (vaccination in the second eligible pregnancy) and social determinants measured at baseline of the first eligible pregnancy, as well as additionally adjusting for the time interval between the end of the first pregnancy and the start of the next.

For peer review only - http://bmjopen.bmj.com/site/about/guidelines.xhtml

Ethics

The study was approved by the Independent Scientific Advisory Group of the CPRD (ISAC, Reference: 17_030) with an amendment to include the secondary analysis (ISAC reference 17_030RA2) and the London School of Hygiene and Tropical Medicine Ethics Committee (Reference: 16265). The amended ISAC protocol was made available to reviewers.

Patient involvement

This research was conducted without patient involvement.

For peer review only

For peer review only - http://bmjopen.bmj.com/site/about/guidelines.xhtml

RESULTS

Sample characteristics

A total of 68,090 women from 402 general practices were eligible for the pertussis vaccine analysis, and 152,132 women from 456 general practices were eligible for the influenza vaccine analysis during the study period (2012 to 2015 for pertussis and 2010/11 to 2015/16 for influenza). Many women were eligible to be offered both pertussis and influenza vaccinations during the study: 66,143 women were included in both analytic samples (97.1% of the pertussis vaccine cohort and 43.5% of the influenza cohort). There were 5,553 (8.9%) and 11,991 (7.9%) women from the pertussis and influenza vaccine analyses, respectively, who had missing ethnicity and were excluded from analysis.

Compared to women with recorded ethnicity, women with missing ethnicity were more likely to have an eligible pregnancy later in the study period, reside in South Central or South East Coast regions of England, have no children living in their household, and to have missing BMI information. Vaccine uptake was similar between women with recorded versus missing ethnicity for pertussis (67.3% vs. 68.2) and influenza (39.1% vs. 40.4%) (all p<0.001, **Supplementary Table 2**).

Primary analyses – pertussis vaccination

Among 62,537 eligible women with recorded ethnicity, maternal pertussis vaccine uptake increased each year, reaching 71.7% in 2015 (**Table 1**). Uptake was also highest in the least deprived areas (76.0%, **Figure 1**) and East and West Midlands (74.5% and 72.9%, respectively), and among women of white ethnicity (69.0%), aged 30-35 years (70.8%), who had no other children living in household (74.4%), who were of normal weight or overweight (69.2% and 69.3%, respectively).

For peer review only - http://bmjopen.bmj.com/site/about/guidelines.xhtml

After adjusting for calendar year, those who resided in the most deprived areas had less than half the odds of vaccine uptake compared to those in the least deprived areas, and those in all regions of England apart from the North East had increased odds of uptake compared to London (**Table 1**). Pertussis vaccination uptake was appreciably lower among all non-white ethnic groups, with reduced odds of between 24% (South Asian) and 55% (Black ethnicity) compared to those of White ethnicity. The odds of vaccination increased non-linearly with maternal age; compared to women aged 20-24 years, women who were <20 years had 21% lower odds of receiving vaccination and there was an increased likelihood of vaccination among women aged ≥25 years, reaching 54% increased odds of uptake among those aged 30-35 years. Uptake decreased linearly with increasing numbers of children living in the household; 33% less likely among women with one child, 53% less likely among women with two children, and 65% less likely among women with three or more children (Table 1). Among the 55,871 women with available BMI data, calendar-year adjusted uptake was 29% less likely among women whose BMI was classified as underweight and 18% less likely among women classified as obese, compared to women with normal BMI (**Table 1**).

Associations in the minimally adjusted models were largely unchanged after additionally adjusting for IMD, region, and ethnicity (Model 1), maternal age (Model 2), and number of children (Model 3). Associations were slightly attenuated (>10% change) for some regions in England (i.e., East of England, South Central, and South East Coast) in Model 1 and Model 2, but not in Model 3. Similarly, associations of pertussis uptake were marginally attenuated in non-white ethnic groups by adjustment for IMD and region (Model 2). However, strong evidence of all

For peer review only - http://bmjopen.bmj.com/site/about/guidelines.xhtml

these associations remained. Model estimates were also robust to the additional adjustment for BMI in the subset of women with non-missing BMI (**Supplementary Table 3**).

Primary analyses – influenza vaccination

Similar to pertussis vaccination, maternal influenza vaccine uptake was highest (46%) by the end of the study period (the 2015/16 season) among the 140,141 eligible women with recorded ethnicity (**Table 2**). Uptake was also highest in the least deprived areas (44.0%, **Figure 1**), in the South Central and West Midlands regions (42.6% and 42.2%, respectively), and among women of white ethnicity (39.8%), aged 30-35 years (41.0%), who had no children living in household (43.0%), and who were overweight (40.4%). Influenza vaccination uptake was lowest among women of Black ethnicity, with 16% reduced odds of uptake compared to those of White ethnicity. Women who were classified as being in a clinical risk group had the highest influenza vaccine uptake (50.9%) out of all subgroups.

Findings of associations between social determinants and influenza vaccine uptake were largely the same as those with pertussis uptake (**Table 2**). Women were 65% more likely to receive the influenza vaccination in the 2015/16 season compared to the 2010/11 season. Similarly, in influenza-season adjusted models, women who resided in the most deprived areas had 29% lower odds of receiving vaccination, and women in all regions outside of London were more likely to be vaccinated. Associations with ethnicity, maternal age, number of children, and BMI also mirrored those found in the pertussis uptake models, although the lower uptake seen with women of non-white ethnicity was less marked than that seen for pertussis

For peer review only - http://bmjopen.bmj.com/site/about/guidelines.xhtml

vaccination. Women identified as being in a clinical risk group for influenza were 69% more likely to be vaccinated than those not in a clinical risk group. Associations were robust throughout all subsequent models except for South Asian ethnicity and South East Coast regional residence, and remained after additional adjustment for clinical risk group in Model 4 (**Table 2**). Model estimates were also robust to the additional adjustment for BMI in the model excluding those with missing BMI (**Supplementary Table 4**).

Sensitivity analyses

Directions of associations and conclusions were robust to all sensitivity analysis for pertussis vaccination (**Supplementary Table 5**) and influenza vaccination (**Supplementary Table 6**), and we found no evidence of clustering at the practice level in the primary analysis models for either pertussis or influenza uptake (ρ=0.07, 95% CI 0.06-0.09 for pertussis, ρ=0.03, 95% CI 0.03-0.03 for influenza).

Secondary analysis

Among women who were included in the main study, there were 3,111 women who received pertussis vaccination in their first eligible pregnancy and who completed a second eligible pregnancy within the study period. Among these, 1,234 (39.7%) were not vaccinated in their second eligible pregnancy. Social determinants of vaccine uptake among women who had previously received vaccination in pregnancy were similar to those in the main analysis, with lower uptake in the second eligible pregnancy associated with younger maternal age at the first pregnancy, a greater

For peer review only - http://bmjopen.bmj.com/site/about/guidelines.xhtml

number of children in the household and a longer interval between pregnancies (**Supplementary Table 7**).

For peer review only

DISCUSSION

Vaccine uptake in pregnancy over the study period was 67.3% for pertussis and 39.1% for influenza. Lower vaccine uptake was associated with greater deprivation: the gap in uptake between the least and most deprived quintiles was almost 10% for influenza, and almost 20% for pertussis. Lower uptake was also associated with non-white ethnicity (particularly Black ethnicity), maternal age under 20 years, and greater number of children in the household. The associations between all social factors and vaccine uptake were largely independent of one another. Among women eligible for pertussis vaccination in two pregnancies and vaccinated in the first, 40% were not vaccinated in their second eligible pregnancy.

To our knowledge, this is the first large study of fully individual-level social determinants of maternal vaccine uptake of seasonal influenza and pertussis in England. Our findings differ from a large national study which found no association between deprivation and pandemic influenza vaccine uptake in pregnancy (although vaccine uptake did increase with maternal age) but the previous study was in the context of the 2010 influenza pandemic.[16] Both the overall uptakes and the patterns of regional variation are consistent with national surveillance and ecological studies. Lower vaccine uptake in London is seen more widely across the vaccination programme.[10, 11, 17, 18] For influenza vaccine, the denominator may be seen as overinclusive as some women may have only a short time period eligible for vaccination (due to pregnancy loss or limited overlap of pregnancy with influenza season), resulting in a low estimate of uptake. For seasonal influenza and pertussis vaccines, previous studies have generally suggested associations consistent with those we observed for deprivation, ethnicity, maternal age and parity or number of children, but studies have been ecological or pseudo-individualised, or were

For peer review only - http://bmjopen.bmj.com/site/about/guidelines.xhtml

underpowered for precise estimates.[17-21, 23] Our findings in a large and nationally representative dataset demonstrate that each of these factors is an independent individual-level determinant of maternal vaccine uptake, outside of a pandemic context.

The novel finding that 40% of women who had been vaccinated in their first eligible pregnancy were not in their second is surprising, and suggests that low vaccine uptake in pregnancy is not fully determined by fixed maternal attitudes to vaccination, but may reflect healthcare access or awareness of the need for vaccination in each pregnancy.

Strengths of this study include the use of the CPRD/LSHTM Pregnancy Register with linked hospital and mortality data and detailed algorithms to identify pregnancy timings and a range of individual-level social determinants among a nationally representative population.[30]

Key limitations include low representation from some regions (in particular the East Midlands), and that not all potentially relevant social factors were available, such as education and religion. We may have over-estimated vaccine uptake as the pregnancy register may not include all pregnancies which ended in a loss without coming to the attention of healthcare workers. We included only timely pertussis vaccinations (before 40 weeks' gestation) which may result in lower uptake estimates than pertussis vaccine uptake by delivery. Our study was also limited to vaccination recorded in primary care records, which could have resulted in some under-recording of influenza vaccination, although maternity-led vaccination services were rare before 2016, and general practitioners are required to document

For peer review only - http://bmjopen.bmj.com/site/about/guidelines.xhtml

vaccinations given outside the surgery. To minimise misclassification we ended our study period prior to the introduction of pertussis vaccination in antenatal settings.

The large differences we observed in vaccine uptake by deprivation and ethnicity indicate a key opportunity to reduce health inequalities. Targeting interventions and improving access to vaccines through primary care and maternity services for pregnant women who live in more deprived areas, are of non-white ethnicity, younger, or have more children may reduce health inequalities, improve overall vaccine uptake, and reduce vaccine-preventable deaths among women and children. In addition to targeted vaccination promotion, wider action is needed to address inequalities in access to timely antenatal care.[35] The drop-off in uptake in second pregnancies suggests a need for awareness-raising of the rationale for passive immunisation of infants and the need for vaccination in each pregnancy. Communications to emphasise the need for vaccination in every pregnancy should be available in a range of locally appropriate languages. Since 2016, pertussis vaccination has been available in maternity services, aiming to increase opportunities for vaccine uptake, and it will be important to ensure that healthcare worker training also captures the importance of vaccination in every pregnancy and to monitor the impact of delivery in alternative settings on inequalities in uptake.

Our study adds to international evidence of health inequalities in vaccination uptake in high-income countries. Studies in the United States have found inequalities in vaccine uptake by insurance type, race/ethnicity and education.[13-15] Our finding of large inequalities in vaccine uptake during pregnancy in England, despite universal healthcare which is free at the point of access, highlights the need for other high-income countries to investigate and address inequalities in vaccine uptake during pregnancy.

For peer review only - http://bmjopen.bmj.com/site/about/guidelines.xhtml

Further research is needed into interventions to reduce inequalities in vaccine uptake during pregnancy,[36] to ensure that future vaccine promotion of these and any future maternal vaccination programmes succeed in narrowing rather than widening the large and multi-faceted health inequalities in early years.

For peer review only

For peer review only - http://bmjopen.bmj.com/site/about/guidelines.xhtml

Acknowledgements

This work uses data provided by patients and collected by the NHS as part of their care and support and would not have been possible without access to this data. The NIHR recognises and values the role of patient data, securely accessed and stored, both in underpinning and leading to improvements in research and care.

Author Contributions

JLW and SLT conceived the main study, and CTR and HIM conceived the secondary analysis. JLW, CTR, HIM, CM, and SLT designed the study. JLW performed the data extraction and JLW and CTR performed the statistical analyses. JB, CTR and HIM designed the secondary analysis, for which JB and HIM performed the statistical analysis. JLW, CTR, HIM, JB, CM, GA, ME and SLT contributed to the interpretation of results. CTR and HIM drafted the manuscript, which JLW, JB, CM, GA, ME and SLT contributed to, revised critically, and approved. HIM is the guarantor. The corresponding author (HIM) attests that all listed authors meet authorship criteria and that no others meeting the criteria have been omitted.

Funding

The research was funded by the National Institute for Health Research (NIHR) Health Protection Research Unit (HPRU) in Immunisation (Grant number IS-HPU-1112-10096) at the London School of Hygiene and Tropical Medicine in partnership with Public Health England (PHE). The views expressed are those of the authors and not necessarily those of the NHS, the NIHR, the Department of Health and Social Care, or PHE.

For peer review only

Competing interests

All authors have completed the ICMJE uniform disclosure form at www.icmje.org/coi_disclosure.pdf and declare: JLW, CTR, HIM and SLT had financial support from the National Institute for Health Research (NIHR) Health Protection Research Unit (HPRU) in Immunisation for the submitted work; Public Health England Immunisation and Countermeasures Division has provided vaccine manufacturers with post-marketing surveillance reports on pneumococcal and meningococcal infection which the companies are required to submit to the UK Licensing Authority in compliance with their Risk Management Strategy, and a cost recovery charge is made for these reports; no other relationships or activities that could appear to have influenced the submitted work.

Ethics approval

The study was approved by the Independent Scientific Advisory Group of the CPRD (ISAC reference 17_030RA2) and the London School of Hygiene and Tropical Medicine Ethics Committee (LSHTM reference 16265). The study protocol was made available to reviewers.

Data sharing

The data used for this study were obtained from the Clinical Practice Research Datalink (CPRD). All data are available via an application to the Independent

For peer review only

For peer review only - http://bmjopen.bmj.com/site/about/guidelines.xhtml

Scientific Advisory Committee (see https://www.cprd.com/Data-access). Data acquisition is associated with a fee.

Transparency

The manuscript's guarantor (HIM) affirms that the manuscript is an honest, accurate, and transparent account of the study being reported; that no important aspects of the study have been omitted; and that any discrepancies from the study as planned have been explained.

Copyright/license

The Corresponding Author has the right to grant on behalf of all authors and does grant on behalf of all authors, a non-exclusive worldwide licence to the Publishers and its licensees in perpetuity, in all forms, formats and media (whether known now or created in the future), to i) publish, reproduce, distribute, display and store the Contribution, ii) translate the Contribution into other languages, create adaptations, reprints, include within collections and create summaries, extracts and/or, abstracts of the Contribution, iii) create any other derivative work(s) based on the Contribution, iv) to exploit all subsidiary rights in the Contribution, v) the inclusion of electronic links from the Contribution to third party material where-ever it may be located; and, vi) licence any third party to do any or all of the above.

For peer review only

References

1. Rasmussen SA, Jamieson DJ, Uyeki TM. Effects of influenza on pregnant women and infants. American journal of obstetrics and gynecology. 2012 Sep;207(3 Suppl):S3-8
2. Public Health England. Immunisation against Infectious Disease (the Green Book). Chapter 24: Pertussis 2016. Available from: https://assets.publishing.service.gov.uk/government/uploads/system/uploads/attachment_data/file/514363/Pertussis_Green_Book_Chapter_24_Ap2016.pdf.
3. van Hoek AJ, Campbell H, Amirthalingam G, Andrews N, Miller E. The number of deaths among infants under one year of age in England with pertussis: results of a capture/recapture analysis for the period 2001 to 2011. Euro Surveillance: Bulletin Europeen sur les Maladies Transmissibles = European Communicable Disease Bulletin. 2013;18(9)
4. Amirthalingam G, Gupta S, Campbell H. Pertussis immunisation and control in England and Wales, 1957 to 2012: a historical review. Euro Surveillance: Bulletin Europeen sur les Maladies Transmissibles = European Communicable Disease Bulletin. 2013;18(38)
5. Thompson MG, Kwong JC, Regan AK, Katz MA, Drews SJ, Azziz-Baumgartner E, et al. Influenza Vaccine Effectiveness in Preventing Influenza-associated Hospitalizations During Pregnancy: A Multi-country Retrospective Test Negative Design Study, 2010-2016. Clinical infectious diseases : an official publication of the Infectious Diseases Society of America. 2019 Apr 24;68(9):1444-53
6. Amirthalingam G, Campbell H, Ribeiro S, Fry NK, Ramsay M, Miller E, et al. Sustained Effectiveness of the Maternal Pertussis Immunization Program in England 3 Years Following Introduction. Clinical infectious diseases : an official publication of the Infectious Diseases Society of America. 2016 Dec 1;63(suppl 4):S236-s43
7. Dabrera G, Zhao H, Andrews N, Begum F, Green H, Ellis J, et al. Effectiveness of seasonal influenza vaccination during pregnancy in preventing influenza infection in infants, England, 2013/14. Euro Surveill. 2014 Nov 13;19(45):20959
8. Public Health England. Immunisation against Infectious Disease (the Green Book). Chapter 19: Influenza. Available from: https://www.gov.uk/government/publications/influenza-the-green-book-chapter-19.
9. Wiley KE, Leask J. Respiratory vaccine uptake during pregnancy. Lancet Respir Med. 2013 Mar;1(1):9-11
10. Public Health England. Pertussis vaccination programme for pregnant women update: vaccine coverage in England, January to March 2019 and 2018/19 annual coverage. Health Protection Report [Internet]. 2019; 13. Available from: https://assets.publishing.service.gov.uk/government/uploads/system/uploads/attachment_data/file/821145/hpr2619_prntl-prtsss_VC.pdf.
11. Public Health England. Seasonal influenza vaccine uptake in GP patients: winter season 2018 to 20192019. Available from: https://assets.publishing.service.gov.uk/government/uploads/system/uploads/attachment_data/file/804889/Seasonal_influenza_vaccine_uptake_in_GP_patients_1819.pdf.
12. Yuen CY, Tarrant M. Determinants of uptake of influenza vaccination among pregnant women - a systematic review. Vaccine. 2014 Aug 6;32(36):4602-13
13. Koepke R, Schauer SL, Davis JP. Measuring maternal Tdap and influenza vaccination rates: Comparison of two population-based methods. Vaccine. 2017 Apr 25;35(18):2298-302

For peer review only - http://bmjopen.bmj.com/site/about/guidelines.xhtml

14. Ding H, Black CL, Ball S, Fink RV, Williams WW, Fiebelkorn AP, et al. Influenza Vaccination Coverage Among Pregnant Women - United States, 2016-17 Influenza Season. MMWR Morb Mortal Wkly Rep. 2017 Sep 29;66(38):1016-22
15. Housey M, Zhang F, Miller C, Lyon-Callo S, McFadden J, Garcia E, et al. Vaccination with tetanus, diphtheria, and acellular pertussis vaccine of pregnant women enrolled in Medicaid--Michigan, 2011-2013. MMWR Morb Mortal Wkly Rep. 2014 Sep 26;63(38):839-42
16. Sammon CJ, McGrogan A, Snowball J, de Vries CS. Pandemic influenza vaccination during pregnancy: an investigation of vaccine uptake during the 2009/10 pandemic vaccination campaign in Great Britain. Human vaccines & immunotherapeutics. 2013 Apr;9(4):917-23
17. Byrne L, Ward C, White JM, Amirthalingam G, Edelstein M. Predictors of coverage of the national maternal pertussis and infant rotavirus vaccination programmes in England. Epidemiol Infect. 2018 Jan;146(2):197-206
18. Tessier E, Warburton F, Tsang C, Rafeeq S, Boddington N, Sinnathamby M, et al. Population-level factors predicting variation in influenza vaccine uptake among adults and young children in England, 2015/16 and 2016/17. Vaccine. 2018 May 31;36(23):3231-8
19. Carlisle N, Seed PT, Gillman L. Can common characteristics be identified as predictors for seasonal influenza vaccine uptake in pregnancy? A retrospective cohort study from a South London Hospital. Midwifery. 2019;72:67-73
20. Wilcox CR, Calvert A, Metz J, Kilich E, MacLeod R, Beadon K, et al. Determinants of Influenza and Pertussis Vaccination Uptake in Pregnancy: A Multicenter Questionnaire Study of Pregnant Women and Healthcare Professionals. The Pediatric infectious disease journal. 2019;38(6):625-30
21. McAuslane H, Utsi L, Wensley A, Coole L. Inequalities in maternal pertussis vaccination uptake: a cross-sectional survey of maternity units. Journal of public health (Oxford, England). 2018;40(1):121-8
22. Maher L, Hope K, Torvaldsen S, Lawrence G, Dawson A, Wiley K, et al. Influenza vaccination during pregnancy: coverage rates and influencing factors in two urban districts in Sydney. Vaccine. 2013 Nov 12;31(47):5557-64
23. Donaldson B, Jain P, Holder BS, Lindsey B, Regan L, Kampmann B. What determines uptake of pertussis vaccine in pregnancy? A cross sectional survey in an ethnically diverse population of pregnant women in London. Vaccine. 2015 Oct 26;33(43):5822-8
24. Herrett E, Gallagher AM, Bhaskaran K, Forbes H, Mathur R, van Staa T, et al. Data Resource Profile: Clinical Practice Research Datalink (CPRD). Int J Epidemiol. 2015 Jun;44(3):827-36
25. Williams T, van Staa T, Puri S, Eaton S. Recent advances in the utility and use of the General Practice Research Database as an example of a UK Primary Care Data resource. Ther Adv Drug Saf. 2012 Apr;3(2):89-99
26. Minassian C, Williams R, Meeraus WH, Smeeth L, Campbell OMR, Thomas SL. Methods to generate and validate a Pregnancy Register in the UK Clinical Practice Research Datalink primary care database. Pharmacoepidemiol Drug Saf. 2019 Jul;28(7):923-33
27. Walker JL, Grint DJ, Strongman H, Eggo RM, Peppa M, Minassian C, et al. UK prevalence of underlying conditions which increase the risk of severe COVID-19 disease: a point prevalence study using electronic health records. BMC Public Health. 2021 2021/03/11;21(1):484

For peer review only - http://bmjopen.bmj.com/site/about/guidelines.xhtml

28. Mathur R, Bhaskaran K, Chaturvedi N, Leon DA, vanStaa T, Grundy E, et al. Completeness and usability of ethnicity data in UK-based primary care and hospital databases. Journal of public health (Oxford, England). 2014 Dec;36(4):684-92

29. Department for Communities and Local Government. The English Index of Multiple Deprivation 2015 – Frequently Asked Questions 2016 [23 January 2020]. Available from: https://assets.publishing.service.gov.uk/government/uploads/system/uploads/attachment_data/file/579151/English_Indices_of_Deprivation_2015_-_Frequently_Asked_Questions_Dec_2016.pdf

30. Jain A, van Hoek AJ, Walker JL, Mathur R, Smeeth L, Thomas SL. Identifying social factors amongst older individuals in linked electronic health records: An assessment in a population based study. PLoS One. 2017;12(11):e0189038

31. Bhaskaran K, Forbes HJ, Douglas I, Leon DA, Smeeth L. Representativeness and optimal use of body mass index (BMI) in the UK Clinical Practice Research Datalink (CPRD). BMJ Open. 2013 Sep 13;3(9):e003389

32. Walker JL, Andrews NJ, Amirthalingam G, Forbes H, Langan SM, Thomas SL. Effectiveness of herpes zoster vaccination in an older United Kingdom population. Vaccine. 2018 04 19;36(17):2371-7

33. Jain A, Walker JL, Mathur R, Forbes HJ, Langan SM, Smeeth L, et al. Zoster vaccination inequalities: A population based cohort study using linked data from the UK Clinical Practice Research Datalink. PLoS One. 2018;13(11):e0207183

34. Victora CG, Huttly SR, Fuchs SC, Olinto MT. The role of conceptual frameworks in epidemiological analysis: a hierarchical approach. Int J Epidemiol. 1997 Feb;26(1):224-7

35. McDonald H, Moren C, Scarlett J. Health inequalities in timely antenatal care: audit of pre- and post-referral delays in antenatal bookings in London 2015–16. Journal of Public Health. 2020;42(4):801-15

36. Crocker-Buque T, Edelstein M, Mounier-Jack S. Interventions to reduce inequalities in vaccine uptake in children and adolescents aged <19 years: a systematic review. J Epidemiol Community Health. 2017 Jan;71(1):87-97

For peer review only - http://bmjopen.bmj.com/site/about/guidelines.xhtml

Table 1. Pertussis vaccine uptake by social characteristics amongst pregnant women in England, 2012 to 2015
N=62,537 from 402 practices. Overall vaccine uptake 42,099 (67.3%)

	**Total** (column %)	**Received pertussis vaccine** unadjusted coverage (row %)	**Minimally adjusted for year** "minimally adjusted" OR (95% CI)	**Model 1** Additionally adjusted for IMD, region, and ethnicity OR (95% CI)	**Model 2** Additionally adjusted for maternal age OR (95% CI)	**Model 3** Additionally adjusted for number of children "fully adjusted" OR (95% CI)
**Year**						
2012	6,717 (10.7%)	3,809 (56.7%)	1	1	1	1
2013	24,657 (39.4%)	16,749 (67.9%)	1.62 (1.53, 1.71)	1.66 (1.57, 1.75)	1.66 (1.57, 1.75)	1.69 (1.60, 1.79)
2014	20,148 (32.2%)	13,638 (67.7%)	1.60 (1.51, 1.69)	1.63 (1.54, 1.73)	1.63 (1.54, 1.73)	1.66 (1.57, 1.76)
2015	11,015 (17.6%)	7,903 (71.7%)	1.94 (1.82, 2.07)	2.00 (1.87, 2.13)	2.00 (1.87, 2.13)	2.03 (1.90, 2.17)
**Index of Multiple Deprivation (IMD) quintile**						
Least deprived	13,285 (21.2%)	10,090 (76.0%)	1	1	1	1
2	11,335 (18.1%)	8,064 (71.1%)	0.78 (0.74, 0.83)	0.79 (0.74, 0.83)	0.80 (0.75, 0.85)	0.81 (0.76, 0.86)
3	12,933 (20.7%)	8,807 (68.1%)	0.68 (0.64, 0.71)	0.68 (0.64, 0.72)	0.70 (0.66, 0.74)	0.73 (0.69, 0.77)
4	12,973 (20.7%)	8,205 (63.2%)	0.54 (0.52, 0.57)	0.56 (0.53, 0.59)	0.59 (0.56, 0.62)	0.64 (0.60, 0.67)
Most deprived	12,011 (19.2%)	6,933 (57.7%)	0.43 (0.41, 0.46)	0.45 (0.42, 0.47)	0.48 (0.45, 0.51)	0.54 (0.51, 0.57)
**Region**						
London	11,894 (19.0%)	7,239 (60.9%)	1	1	1	1
North East	1,185 (1.9%)	687 (58.0%)	0.91 (0.81, 1.03)	0.96 (0.85, 1.09)	1.00 (0.88, 1.13)	1.04 (0.92, 1.19)
North West	8,835 (14.1%)	5,873 (66.5%)	1.29 (1.22, 1.36)	1.28 (1.20, 1.35)	1.30 (1.22, 1.38)	1.36 (1.27, 1.44)
Yorkshire & The Humber	1,000 (1.6%)	699 (69.9%)	1.51 (1.31, 1.74)	1.46 (1.27, 1.69)	1.51 (1.30, 1.74)	1.54 (1.33, 1.79)
East Midlands	326 (0.5%)	243 (74.5%)	2.18 (1.69, 2.81)	2.24 (1.73, 2.90)	2.30 (1.78, 2.98)	2.38 (1.84, 3.09)
West Midlands	7,050 (11.3%)	5,046 (71.6%)	1.64 (1.54, 1.75)	1.58 (1.48, 1.69)	1.62 (1.52, 1.73)	1.72 (1.61, 1.84)
East of England	5,568 (8.9%)	4,058 (72.9%)	1.75 (1.63, 1.88)	1.50 (1.40, 1.61)	1.52 (1.41, 1.63)	1.57 (1.46, 1.69)
South West	7,002 (11.2%)	4,800 (68.6%)	1.43 (1.34, 1.52)	1.32 (1.24, 1.41)	1.35 (1.26, 1.44)	1.43 (1.33, 1.52)
South Central	10,381 (16.6%)	7,185 (69.2%)	1.45 (1.37, 1.53)	1.19 (1.12, 1.26)	1.21 (1.15, 1.29)	1.28 (1.21, 1.36)
South East Coast	9,296 (14.9%)	6,269 (67.4%)	1.33 (1.26, 1.41)	1.10 (1.04, 1.17)	1.12 (1.06, 1.19)	1.19 (1.12, 1.26)
**Ethnicity**						
White	52,598 (84.1%)	36,272 (69.0%)	1	1	1	1
South Asian	4,692 (7.5%)	2,951 (62.9%)	0.76 (0.71, 0.81)	0.83 (0.78, 0.88)	0.79 (0.74, 0.85)	0.83 (0.78, 0.88)
Black	2,583 (4.1%)	1,294 (50.1%)	0.45 (0.41, 0.48)	0.58 (0.54, 0.64)	0.56 (0.52, 0.61)	0.61 (0.56, 0.67)
Mixed	922 (1.5%)	549 (59.5%)	0.65 (0.57, 0.74)	0.72 (0.63, 0.82)	0.71 (0.62, 0.82)	0.72 (0.63, 0.83)

For peer review only - http://bmjopen.bmj.com/site/about/guidelines.xhtml

Other	1,742 (2.8%)	1,033 (59.3%)	0.65 (0.59, 0.72)	0.73 (0.66, 0.80)	0.70 (0.63, 0.77)	0.68 (0.62, 0.75)
**Maternal age, years**						
<20	2,079 (3.3%)	1,153 (55.5%)	0.79 (0.72, 0.87)		0.80 (0.73, 0.89)	0.81 (0.73, 0.89)
20-24	8,848 (14.1%)	5,416 (61.2%)	1		1	1
25-29	16,696 (26.7%)	11,166 (66.9%)	1.27 (1.21, 1.34)		1.24 (1.18, 1.31)	1.29 (1.22, 1.36)
30-35	20,294 (32.5%)	14,376 (70.8%)	1.54 (1.46, 1.62)		1.43 (1.35, 1.51)	1.55 (1.47, 1.64)
≥35	14,620 (23.4%)	9,988 (68.3%)	1.36 (1.29, 1.44)		1.25 (1.18, 1.32)	1.42 (1.34, 1.51)
**Number of children**						
0	26,622 (42.6%)	19,814 (74.4%)	1			1
1	22,132 (35.4%)	14,673 (66.3%)	0.67 (0.65, 0.70)			0.65 (0.63, 0.68)
2	8,645 (13.8%)	5,009 (57.9%)	0.47 (0.45, 0.49)			0.47 (0.45, 0.50)
≥3	5,138 (8.2%)	2,603 (50.7%)	0.35 (0.33, 0.37)			0.37 (0.35, 0.40)
**Body Mass Index (BMI)**						
<18.5 underweight	2,063 (3.3%)	1,265 (61.3%)	0.71 (0.64, 0.77)			
18.5-24.9	29,045 (46.4%)	20,095 (69.2%)	1			
25.0-29.9 overweight	14,211 (22.7%)	9,852 (69.3%)	1.01 (0.96, 1.05)			
≥30 obese	10,552 (16.9%)	6,833 (64.8%)	0.82 (0.78, 0.86)			
Missing	6,666 (10.7%)	4,054 (60.8%)				

OR, odds ratio; CI, confidence interval.

Note: All models include women who registered before the end of the first trimester and delivered a live-or stillborn child on or after 26 weeks of pregnancy and exclude those with missing ethnicity; minimally adjusted model of BMI additionally excludes 6,666 women with missing BMI

For peer review only - http://bmjopen.bmj.com/site/about/guidelines.xhtml

Table 2. Influenza vaccine uptake by social characteristics amongst pregnant women in England, 2010/11 to 2015/16
N=140,141 from 456 practices. Overall vaccine uptake 54,837 (39.1%)

	**Total** (column %)	**Received influenza vaccine** unadjusted coverage (row %)	**Minimally adjusted for year** "minimally adjusted" OR (95% CI)	**Model 1** Additionally adjusted for IMD, region, and ethnicity OR (95% CI)	**Model 2** Additionally adjusted for maternal age OR (95% CI)	**Model 3** Additionally adjusted for number of children OR (95% CI)	**Model 4** Additionally adjusted for clinical risk group "fully adjusted" OR (95% CI)
**Season**							
2010	34,373 (24.5%)	11,703 (34.0%)	1	1	1	1	1
2011	32,258 (23.0%)	10,151 (31.5%)	0.89 (0.86, 0.92)	0.89 (0.86, 0.92)	0.89 (0.86, 0.92)	0.89 (0.86, 0.92)	0.89 (0.86, 0.92)
2012	26,750 (19.1%)	12,236 (45.7%)	1.63 (1.58, 1.69)	1.66 (1.61, 1.72)	1.66 (1.61, 1.72)	1.64 (1.59, 1.70)	1.65 (1.60, 1.71)
2013	21,029 (15.0%)	8,815 (41.9%)	1.40 (1.35, 1.45)	1.43 (1.38, 1.48)	1.42 (1.37, 1.47)	1.39 (1.35, 1.45)	1.40 (1.35, 1.45)
2014	15,712 (11.2%)	7,319 (46.6%)	1.69 (1.63, 1.76)	1.74 (1.67, 1.80)	1.73 (1.67, 1.80)	1.69 (1.63, 1.76)	1.70 (1.63, 1.76)
2015	10,019 (7.1%)	4,613 (46.0%)	1.65 (1.58, 1.73)	1.72 (1.65, 1.80)	1.72 (1.64, 1.80)	1.68 (1.60, 1.76)	1.68 (1.60, 1.76)
**Index of Multiple Deprivation (IMD) quintile**							
Least deprived	28,956 (20.7%)	12,744 (44.0%)	1	1	1	1	1
2	25,424 (18.1%)	10,533 (41.4%)	0.90 (0.87, 0.93)	0.91 (0.88, 0.94)	0.92 (0.89, 0.95)	0.93 (0.89, 0.96)	0.92 (0.89, 0.95)
3	29,368 (21.0%)	11,670 (39.7%)	0.84 (0.81, 0.86)	0.84 (0.82, 0.87)	0.86 (0.83, 0.89)	0.88 (0.85, 0.91)	0.88 (0.85, 0.91)
4	28,520 (20.4%)	10,278 (36.0%)	0.71 (0.69, 0.74)	0.72 (0.69, 0.74)	0.74 (0.71, 0.77)	0.77 (0.74, 0.79)	0.76 (0.74, 0.79)
Most deprived	27,873 (19.9%)	9,612 (34.5%)	0.67 (0.65, 0.70)	0.66 (0.64, 0.68)	0.69 (0.66, 0.71)	0.73 (0.70, 0.76)	0.72 (0.70, 0.75)
**Region**							
London	26,171 (18.7%)	9,146 (34.9%)	1	1	1	1	1
North East	2,758 (2.0%)	989 (35.9%)	1.11 (1.02, 1.21)	1.16 (1.07, 1.27)	1.19 (1.09, 1.29)	1.21 (1.11, 1.31)	1.21 (1.11, 1.31)
North West	19,060 (13.6%)	7,870 (41.3%)	1.37 (1.32, 1.42)	1.39 (1.33, 1.45)	1.40 (1.35, 1.46)	1.43 (1.37, 1.49)	1.42 (1.36, 1.47)
Yorkshire & The Humber	2,840 (2.0%)	1,090 (38.4%)	1.27 (1.18, 1.38)	1.24 (1.15, 1.35)	1.26 (1.16, 1.37)	1.26 (1.16, 1.37)	1.26 (1.16, 1.37)
East Midlands	1,940 (1.4%)	717 (37.0%)	1.33 (1.21, 1.47)	1.37 (1.24, 1.51)	1.39 (1.26, 1.53)	1.41 (1.27, 1.55)	1.40 (1.27, 1.55)
West Midlands	15,846 (11.3%)	6,692 (42.2%)	1.41 (1.35, 1.46)	1.40 (1.34, 1.46)	1.41 (1.36, 1.47)	1.44 (1.38, 1.51)	1.43 (1.37, 1.49)
East of England	13,695 (9.8%)	5,468 (39.9%)	1.31 (1.26, 1.37)	1.23 (1.18, 1.29)	1.24 (1.19, 1.29)	1.25 (1.20, 1.31)	1.24 (1.19, 1.30)
South West	16,546 (11.8%)	6,504 (39.3%)	1.25 (1.20, 1.31)	1.22 (1.17, 1.28)	1.24 (1.19, 1.29)	1.27 (1.21, 1.32)	1.25 (1.20, 1.31)
South Central	21,435 (15.3%)	9,125 (42.6%)	1.42 (1.36, 1.47)	1.30 (1.25, 1.35)	1.31 (1.26, 1.36)	1.34 (1.29, 1.39)	1.33 (1.28, 1.38)
South East Coast	19,850 (14.2%)	7,236 (36.5%)	1.06 (1.02, 1.10)	0.99 (0.95, 1.03)	1.00 (0.96, 1.04)	1.02 (0.98, 1.06)	1.02 (0.98, 1.06)
**Ethnicity**							
White	117,469 (83.8%)	46,781 (39.8%)	1	1	1	1	1
South Asian	10,827 (7.7%)	4,103 (37.9%)	0.92 (0.88, 0.95)	0.98 (0.94, 1.02)	0.96 (0.92, 1.00)	0.98 (0.94, 1.02)	0.99 (0.95, 1.03)
Black	5,853 (4.2%)	1,837 (31.4%)	0.67 (0.64, 0.71)	0.81 (0.76, 0.86)	0.80 (0.75, 0.85)	0.83 (0.78, 0.88)	0.83 (0.78, 0.88)
Mixed	2,094 (1.5%)	757 (36.2%)	0.84 (0.77, 0.92)	0.90 (0.82, 0.99)	0.90 (0.82, 0.99)	0.91 (0.83, 0.99)	0.91 (0.83, 0.99)
Other	3,898 (2.8%)	1,359 (34.9%)	0.79 (0.73, 0.84)	0.85 (0.80, 0.91)	0.84 (0.78, 0.90)	0.83 (0.78, 0.89)	0.85 (0.79, 0.91)

For peer review only - http://bmjopen.bmj.com/site/about/guidelines.xhtml

**Maternal age, years**							
<20	5,536 (4.0%)	1,817 (32.8%)	0.87 (0.81, 0.92)		0.87 (0.82, 0.93)	0.87 (0.82, 0.93)	0.87 (0.82, 0.93)
20-24	21,663 (15.5%)	7,797 (36.0%)	1		1	1	1
25-29	37,985 (27.1%)	14,827 (39.0%)	1.13 (1.09, 1.17)		1.11 (1.07, 1.15)	1.12 (1.09, 1.16)	1.12 (1.08, 1.16)
30-35	43,777 (31.2%)	17,950 (41.0%)	1.22 (1.18, 1.26)		1.18 (1.14, 1.22)	1.21 (1.17, 1.26)	1.21 (1.17, 1.25)
≥35	31,180 (22.2%)	12,446 (39.9%)	1.17 (1.12, 1.21)		1.12 (1.08, 1.16)	1.19 (1.15, 1.24)	1.18 (1.13, 1.22)
**Number of children**							
0	66,112 (47.2%)	28,457 (43.0%)	1			1	1
1	45,969 (32.8%)	17,092 (37.2%)	0.80 (0.78, 0.82)			0.80 (0.78, 0.82)	0.80 (0.78, 0.82)
2	18,192 (13.0%)	6,242 (34.3%)	0.71 (0.68, 0.73)			0.72 (0.69, 0.74)	0.71 (0.69, 0.74)
≥3	9,868 (7.0%)	3,046 (30.9%)	0.61 (0.58, 0.63)			0.63 (0.60, 0.66)	0.62 (0.59, 0.65)
**Clinical risk group recommended for influenza vaccination**							
No	130,160 (92.9%)	49,752 (38.2%)	1				1
Yes	9,981 (7.1%)	5,085 (50.9%)	1.69 (1.62, 1.76)				1.70 (1.63, 1.77)
**Body Mass Index (BMI)**							
<18.5 Underweight	4,865 (3.5%)	1,744 (35.8%)	0.85 (0.80, 0.90)				
18.5-24.9	66,405 (47.4%)	26,331 (39.7%)	1				
25.0-29.9 Overweight	31,855 (22.7%)	12,882 (40.4%)	1.04 (1.01, 1.07)				
≥30 Obese	23,142 (16.5%)	9,222 (39.8%)	1.00 (0.97, 1.03)				
Missing	13,874 (9.9%)	4,658 (33.6%)					

OR, odds ratio; CI, confidence interval.

Note: All models include women who registered before the end of the first trimester, and exclude those with no recorded pregnancy outcome or missing ethnicity; minimally adjusted model of BMI additionally excludes 13,874 women with missing BMI

For peer review only - http://bmjopen.bmj.com/site/about/guidelines.xhtml

Figure legend

Figure 1: Unadjusted pertussis and influenza vaccine coverage in pregnancy, by deprivation

For peer review only

For peer review only - http://bmjopen.bmj.com/site/about/guidelines.xhtml

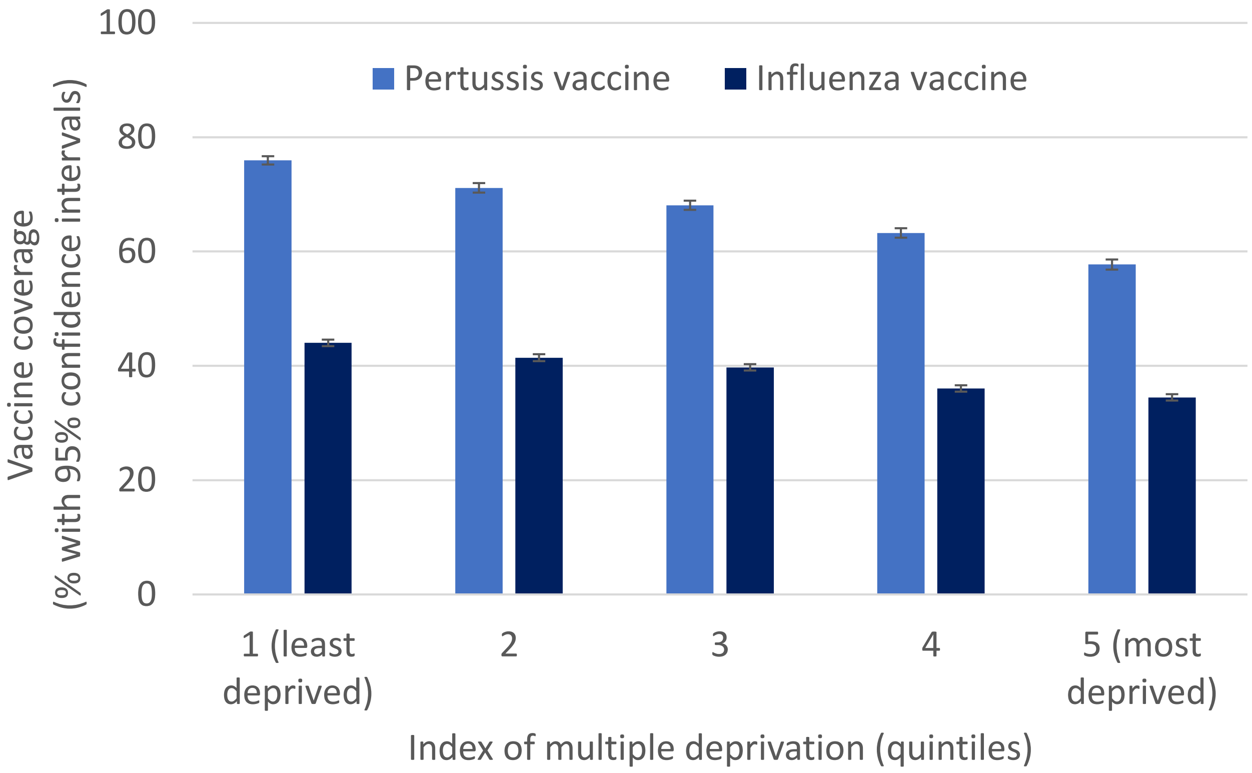
100
80
60
40
20
0
Pertussis vaccine
Influenza vaccine
Vaccine coverage
(% with 95% confidence intervals)
1 (least deprived)
2
3
4
5 (most deprived)
Index of multiple deprivation (quintiles)

Supplementary material

Social determinants of pertussis and influenza vaccine uptake in pregnancy: a national cohort study using electronic health records

Authors: Jemma L Walker,* Christopher T Rentsch,* Helen I McDonald, Jeongeun Bak, Caroline Minassian, Gayatri Amirthalingam, Michael Edelstein, Sara L Thomas.

Supplementary Table 1: Hierarchical conceptual framework and interpretation of effect estimates

Supplementary Table 2: Patterns of social factors amongst pregnant women with and without a recorded ethnicity status, 2010-2015

Supplementary Table 3: 'Pertussis BMI Model' complete case analysis additionally excluding 6,666 women with missing BMI for pertussis vaccine uptake amongst pregnant women in the UK, 2012-2015

Supplementary Table 4: 'Influenza BMI Model' complete case analysis additionally excluding 13,874 women with missing BMI for influenza vaccine uptake amongst pregnant women in the UK, 2010-2015

Supplementary Table 5: Sensitivity analyses expanding definition of inclusion criteria for the pertussis vaccine uptake models: registration by end of pregnancy and ImmForm approach compared to primary analyses

Supplementary Table 7: Secondary analysis of subsequent pertussis vaccine uptake among women who had received pertussis vaccination in their first eligible pregnancy and had a second eligible pregnancy within the study period (N=3,111)

For peer review only

For peer review only - http://bmjopen.bmj.com/site/about/guidelines.xhtml

Supplementary Table 1: Hierarchical conceptual framework and interpretation of effect estimates

This table is reproduced from Supplementary Table 6 in Jain A., Walker JL, Forbes H, Langan S, Smeeth L, van Hoek AJ and Thomas SL. Zoster vaccination inequalities: A population based cohort study using linked data from the UK Clinical Practice Research Datalink. PLoS One 2018;13(11):e0207183. doi: 10.1371/journal.pone.0207183.

(based on [1])

Hierarchical models	Explanatory variables	Interpretation of effect estimates
`Minimally' adjusted model	Each explanatory variable adjusted in-turn for *a priori* confounders: year of birth and gender	Effect estimate of each variable adjusted for *a priori* confounders.
Model-1*^	Ethnicity +immigration status^ with *a priori* confounders	Effects of ethnicity and immigration status adjusted for each other and *a priori* confounders
Model-2*	Model-1+ patient-LSOA-level deprivation#	(i) Effects of ethnicity and immigration status not mediated via deprivation and adjusted for each other and *a priori* confounders<br>(ii) Effect of patient-LSOA-level deprivation adjusted for *a priori* confounders, ethnicity and immigration status
Model-3*	Model-2 + rest of the explanatory variables~	(i) Effect of ethnicity and immigration status not mediated via deprivation and other explanatory variables~ *<br>(ii) Effect of deprivation not mediated via other explanatory variables~*<br>(iii) Effect of other explanatory variables~ *

*all variables in the model adjusted for each other and *a priori* confounders: year of birth, sex and calendar period ^ethnicity and immigration status examined for multicollinearity LSOA Lower-layer Super Output Area # patient-LSOA-level and practice-LSOA-level deprivation were considered to be correlated therefore only patient-LSOA-level deprivation used ~ care home residence, living alone status and cohabitation status (living alone and cohabitation examined for multicollinearity)

1. Victora CG, Huttly SR, Fuchs SC, Olinto MT. The role of conceptual frameworks in epidemiological analysis: a hierarchical approach. Int J Epidemiol. 1997;26(1):224-7. PubMed PMID: 9126524.

For peer review only - http://bmjopen.bmj.com/site/about/guidelines.xhtml

Supplementary Table 2: Patterns of social factors amongst pregnant women with and without a recorded ethnicity status, 2010-2015

		Pertussis		Influenza	
		Recorded ethnicity	Missing ethnicity	Recorded ethnicity	Missing ethnicity
		n=62,537	n=5,553	n=140,141	n=11,991
Year/season	2010	-	-	34,373 (24.5%)	2,433 (20.3%)
	2011	-	-	32,258 (23.0%)	2,228 (18.6%)
	2012	6,717 (10.7%)	506 (9.1%)	26,750 (19.1%)	1,791 (14.9%)
	2013	24,657 (39.4%)	1,789 (32.2%)	21,029 (15.0%)	1,730 (14.4%)
	2014	20,148 (32.2%)	1,910 (34.4%)	15,712 (11.2%)	1,882 (15.7%)
	2015	11,015 (17.6%)	1,348 (24.3%)	10,019 (7.1%)	1,927 (16.1%)
Index of multiple deprivation (IMD) quintile	Least deprived	13,285 (21.2%)	1,522 (27.4%)	28,956 (20.7%)	3,203 (26.7%)
	2	11,335 (18.1%)	883 (15.9%)	25,424 (18.1%)	1,896 (15.8%)
	3	12,933 (20.7%)	992 (17.9%)	29,368 (21.0%)	2,245 (18.7%)
	4	12,973 (20.7%)	1,592 (28.7%)	28,520 (20.4%)	3,265 (27.2%)
	Most deprived	12,011 (19.2%)	564 (10.2%)	27,873 (19.9%)	1,382 (11.5%)
Region	London	11,894 (19.0%)	502 (9.0%)	26,171 (18.7%)	1,144 (9.5%)
	North East	1,185 (1.9%)	60 (1.1%)	2,758 (2.0%)	173 (1.4%)
	North West	8,835 (14.1%)	917 (16.5%)	19,060 (13.6%)	1,761 (14.7%)
	Yorkshire & The Humber	1,000 (1.6%)	5 (0.1%)	2,840 (2.0%)	24 (0.2%)
	East Midlands	326 (0.5%)	70 (1.3%)	1,940 (1.4%)	435 (3.6%)
	West Midlands	7,050 (11.3%)	530 (9.5%)	15,846 (11.3%)	1,231 (10.3%)
	East of England	5,568 (8.9%)	464 (8.4%)	13,695 (9.8%)	1,025 (8.5%)
	South West	7,002 (11.2%)	223 (4.0%)	16,546 (11.8%)	574 (4.8%)
	South Central	10,381 (16.6%)	1,692 (30.5%)	21,435 (15.3%)	3,215 (26.8%)
	South East Coast	9,296 (14.9%)	1,090 (19.6%)	19,850 (14.2%)	2,409 (20.1%)
Ethnicity	White	52,598 (84.1%)	-	117,469 (83.8%)	-
	South Asian	4,692 (7.5%)	-	10,827 (7.7%)	-
	Black	2,583 (4.1%)	-	5,853 (4.2%)	-
	Mixed	922 (1.5%)	-	2,094 (1.5%)	-
	Other	1,742 (2.8%)	-	3,898 (2.8%)	-
Maternal age, years	<20	2,079 (3.3%)	218 (3.9%)	5,536 (4.0%)	583 (4.9%)
	20-24	8,848 (14.1%)	914 (16.5%)	21,663 (15.5%)	2,014 (16.8%)
	25-29	16,696 (26.7%)	1,391 (25.0%)	37,985 (27.1%)	3,004 (25.1%)
	30-35	20,294 (32.5%)	1,673 (30.1%)	43,777 (31.2%)	3,639 (30.3%)
	≥35	14,620 (23.4%)	1,357 (24.4%)	31,180 (22.2%)	2,751 (22.9%)
Number of children	0	26,622 (42.6%)	2,645 (47.6%)	66,112 (47.2%)	6,255 (52.2%)
	1	22,132 (35.4%)	1,675 (30.2%)	45,969 (32.8%)	3,312 (27.6%)
	2	8,645 (13.8%)	679 (12.2%)	18,192 (13.0%)	1,431 (11.9%)
	≥3	5,138 (8.2%)	554 (10.0%)	9,868 (7.0%)	993 (8.3%)
Clinical risk group	No	-	-	130,160 (92.9%)	11,238 (93.7%)
	Yes	-	-	9,981 (7.1%)	753 (6.3%)
Body mass index (BMI)	<18.5	2,063 (3.3%)	201 (3.6%)	4,865 (3.5%)	434 (3.6%)
	18.5-24.9	29,045 (46.4%)	2,489 (44.8%)	66,405 (47.4%)	5,571 (46.5%)
	25.0-29.9	14,211 (22.7%)	1,203 (21.7%)	31,855 (22.7%)	2,563 (21.4%)
	≥30	10,552 (16.9%)	785 (14.1%)	23,142 (16.5%)	1,747 (14.6%)
	Missing	6,666 (10.7%)	875 (15.8%)	13,874 (9.9%)	1,676 (14.0%)

Note: all p<0.001

For peer review only

Supplementary Table 3: 'Pertussis BMI Model' complete case analysis additionally excluding 6,666 women with missing BMI for pertussis vaccine uptake amongst pregnant women in the UK, 2012-2015

		**Minimally adjusted** for year	**Model 3 (fully adjusted in main analysis)** Adjusted for year, IMD, region, ethnicity, maternal age and number of children	**BMI Model** As Model 3 and additionally adjusted for BMI
N		55,871	55,871	55,871
Year	2012	1	1	1
	2013	1.65 (1.56, 1.75)	1.74 (1.63, 1.84)	1.74 (1.63, 1.84)
	2014	1.63 (1.54, 1.73)	1.70 (1.60, 1.81)	1.70 (1.60, 1.81)
	2015	1.95 (1.82, 2.08)	2.04 (1.90, 2.19)	2.04 (1.91, 2.19)
Index of multiple deprivation (IMD) quintile	Least deprived	1	1	1
	2	0.78 (0.73, 0.83)	0.81 (0.76, 0.86)	0.81 (0.76, 0.86)
	3	0.67 (0.64, 0.71)	0.72 (0.68, 0.77)	0.72 (0.68, 0.77)
	4	0.54 (0.51, 0.58)	0.63 (0.59, 0.67)	0.63 (0.59, 0.67)
	Most deprived	0.44 (0.41, 0.46)	0.53 (0.50, 0.57)	0.54 (0.50, 0.57)
Region	London	1	1	1
	North East	0.96 (0.84, 1.10)	1.12 (0.98, 1.29)	1.12 (0.97, 1.28)
	North West	1.30 (1.22, 1.38)	1.36 (1.28, 1.46)	1.36 (1.28, 1.46)
	Yorkshire & The Humber	1.49 (1.29, 1.72)	1.51 (1.30, 1.76)	1.51 (1.30, 1.76)
	East Midlands	1.87 (1.44, 2.42)	2.33 (1.78, 3.04)	2.31 (1.77, 3.02)
	West Midlands	1.60 (1.50, 1.71)	1.70 (1.59, 1.83)	1.70 (1.58, 1.82)
	East of England	1.73 (1.60, 1.86)	1.56 (1.44, 1.68)	1.56 (1.44, 1.68)
	South West	1.41 (1.32, 1.51)	1.42 (1.33, 1.53)	1.42 (1.33, 1.53)
	South Central	1.46 (1.37, 1.55)	1.29 (1.21, 1.37)	1.29 (1.21, 1.37)
	South East Coast	1.33 (1.26, 1.42)	1.18 (1.11, 1.26)	1.18 (1.11, 1.26)
Ethnicity	White	1	1	1
	South Asian	0.74 (0.70, 0.79)	0.83 (0.77, 0.89)	0.83 (0.77, 0.89)
	Black	0.45 (0.41, 0.49)	0.62 (0.57, 0.68)	0.62 (0.56, 0.67)
	Mixed	0.69 (0.60, 0.79)	0.75 (0.65, 0.87)	0.75 (0.65, 0.87)
	Other	0.63 (0.57, 0.70)	0.67 (0.60, 0.75)	0.68 (0.61, 0.75)
Maternal age, years	<20	0.85 (0.75, 0.96)	0.84 (0.74, 0.96)	0.85 (0.75, 0.97)
	20-24	1	1	1
	25-29	1.27 (1.20, 1.34)	1.28 (1.20, 1.36)	1.27 (1.20, 1.35)
	30-35	1.49 (1.41, 1.58)	1.51 (1.42, 1.60)	1.49 (1.41, 1.58)
	≥35	1.32 (1.24, 1.40)	1.37 (1.29, 1.46)	1.36 (1.28, 1.45)
Number of children	0	1	1	1
	1	0.67 (0.65, 0.70)	0.66 (0.63, 0.68)	0.66 (0.63, 0.68)
	2	0.47 (0.44, 0.49)	0.47 (0.45, 0.50)	0.47 (0.45, 0.50)
	≥3	0.35 (0.33, 0.38)	0.37 (0.35, 0.40)	0.37 (0.35, 0.40)
Body mass index (BMI)	<18.5	0.71 (0.64, 0.77)		0.77 (0.70, 0.85)
	18.5-24.9	1		1
	25.0-29.9	1.01 (0.96, 1.05)		1.10 (1.05, 1.15)
	≥30	0.82 (0.78, 0.86)		0.96 (0.91, 1.00)
Note: Model inclusion as per the main analysis but additionally excluding 6,666 women with missing BMI				

For peer review only - http://bmjopen.bmj.com/site/about/guidelines.xhtml

Supplementary Table 4: 'Influenza BMI Model' complete case analysis additionally excluding 13,874 women with missing BMI for influenza vaccine uptake amongst pregnant women in the UK, 2010-2015

		**Minimally adjusted** for year	**Model 4** adjusted for year, IMD,region, ethnicity, maternal age, number of children and clinical risk group	**BMI Model** as Model 4 and additionally adjusted for BMI
N		126,267	126,267	126,267
Year	2010	1	1	1
	2011	0.90 (0.87, 0.93)	0.90 (0.87, 0.93)	0.90 (0.87, 0.93)
	2012	1.63 (1.57, 1.68)	1.64 (1.59, 1.70)	1.64 (1.59, 1.70)
	2013	1.41 (1.36, 1.46)	1.41 (1.35, 1.46)	1.40 (1.35, 1.46)
	2014	1.70 (1.63, 1.77)	1.70 (1.63, 1.77)	1.70 (1.63, 1.77)
	2015	1.66 (1.58, 1.74)	1.67 (1.60, 1.76)	1.67 (1.59, 1.76)
Index of multiple deprivation (IMD) quintile	Least deprived	1	1	1
	2	0.90 (0.87, 0.94)	0.92 (0.89, 0.95)	0.92 (0.88, 0.95)
	3	0.83 (0.80, 0.86)	0.87 (0.84, 0.90)	0.86 (0.83, 0.90)
	4	0.71 (0.69, 0.74)	0.76 (0.73, 0.79)	0.75 (0.73, 0.78)
	Most deprived	0.68 (0.65, 0.70)	0.72 (0.69, 0.75)	0.71 (0.69, 0.74)
Region	London	1	1	1
	North East	1.17 (1.07, 1.28)	1.26 (1.15, 1.37)	1.25 (1.14, 1.37)
	North West	1.40 (1.34, 1.45)	1.43 (1.37, 1.49)	1.43 (1.37, 1.49)
	Yorkshire & The Humber	1.28 (1.18, 1.39)	1.26 (1.16, 1.37)	1.25 (1.15, 1.36)
	East Midlands	1.29 (1.17, 1.43)	1.35 (1.22, 1.49)	1.34 (1.21, 1.49)
	West Midlands	1.41 (1.35, 1.47)	1.44 (1.37, 1.50)	1.43 (1.37, 1.49)
	East of England	1.30 (1.24, 1.36)	1.23 (1.17, 1.28)	1.22 (1.17, 1.28)
	South West	1.29 (1.23, 1.34)	1.28 (1.22, 1.34)	1.27 (1.22, 1.33)
	South Central	1.45 (1.39, 1.51)	1.35 (1.30, 1.41)	1.35 (1.29, 1.40)
	South East Coast	1.08 (1.04, 1.12)	1.03 (0.99, 1.07)	1.03 (0.99, 1.07)
Ethnicity	White	1	1	1
	South Asian	0.90 (0.87, 0.94)	0.99 (0.95, 1.03)	0.99 (0.95, 1.04)
	Black	0.66 (0.62, 0.70)	0.83 (0.78, 0.88)	0.82 (0.77, 0.87)
	Mixed	0.85 (0.78, 0.94)	0.92 (0.84, 1.01)	0.92 (0.84, 1.01)
	Other	0.78 (0.73, 0.84)	0.86 (0.80, 0.92)	0.87 (0.80, 0.93)
Maternal age, years	<20	0.90 (0.84, 0.98)	0.90 (0.83, 0.97)	0.91 (0.84, 0.98)
	20-24	1	1	1
	25-29	1.12 (1.08, 1.16)	1.11 (1.07, 1.15)	1.11 (1.07, 1.15)
	30-35	1.20 (1.16, 1.24)	1.19 (1.15, 1.23)	1.19 (1.14, 1.23)
	≥35	1.14 (1.10, 1.18)	1.15 (1.11, 1.20)	1.15 (1.10, 1.19)
Number of children	0	1	1	1
	1	0.80 (0.78, 0.82)	0.79 (0.77, 0.81)	0.79 (0.77, 0.81)
	2	0.70 (0.68, 0.73)	0.71 (0.68, 0.73)	0.70 (0.68, 0.73)
	≥3	0.61 (0.58, 0.64)	0.62 (0.59, 0.66)	0.62 (0.59, 0.65)
Clinical risk group	No	1	1	1
	Yes	1.69 (1.62, 1.76)	1.69 (1.62, 1.77)	1.68 (1.61, 1.76)
Body mass index (BMI)	<18.5	0.85 (0.80, 0.90)		0.89 (0.84, 0.95)
	18.5-24.9	1		1
	25.0-29.9	1.04 (1.01, 1.07)		1.07 (1.04, 1.10)
	≥30	1.00 (0.97, 1.03)		1.06 (1.03, 1.09)
Note: Model inclusion as per the main analysis but additionally excluding 13,874 women with missing BMI				

For peer review only - http://bmjopen.bmj.com/site/about/guidelines.xhtml

Supplementary Table 5: Sensitivity analyses expanding definition of inclusion criteria for the pertussis vaccine uptake models: registration by end of pregnancy and ImmForm approach compared to primary analyses

		Primary analyses		Registered by end of pregnancy		ImmForm approach	
		Minimally adjusted	Fully adjusted	Minimally adjusted	Fully adjusted	Minimally adjusted	Fully adjusted
N		62,537	62,537	80,831	80,831	90,720	90,720
Year	2012	1	1	1	1	1	1
	2013	1.62 (1.53, 1.71)	1.69 (1.60, 1.79)	1.59 (1.52, 1.67)	1.65 (1.58, 1.73)	1.55 (1.48, 1.62)	1.60 (1.53, 1.67)
	2014	1.60 (1.51, 1.69)	1.66 (1.57, 1.76)	1.69 (1.61, 1.77)	1.72 (1.64, 1.81)	1.64 (1.57, 1.72)	1.67 (1.60, 1.75)
	2015	1.94 (1.82, 2.07)	2.03 (1.90, 2.17)	2.09 (1.98, 2.21)	2.13 (2.02, 2.26)	2.04 (1.94, 2.15)	2.07 (1.96, 2.19)
Index of multiple deprivation (IMD) quintile	Least deprived	1	1	1	1	1	1
	2	0.78 (0.74, 0.83)	0.81 (0.76, 0.86)	0.79 (0.76, 0.83)	0.83 (0.79, 0.87)	0.79 (0.76, 0.83)	0.83 (0.79, 0.87)
	3	0.68 (0.64, 0.71)	0.73 (0.69, 0.77)	0.71 (0.68, 0.74)	0.78 (0.74, 0.81)	0.70 (0.67, 0.73)	0.76 (0.73, 0.80)
	4	0.54 (0.52, 0.57)	0.64 (0.60, 0.67)	0.58 (0.56, 0.61)	0.69 (0.66, 0.73)	0.58 (0.56, 0.61)	0.69 (0.66, 0.72)
	Most deprived	0.43 (0.41, 0.46)	0.54 (0.51, 0.57)	0.46 (0.44, 0.49)	0.59 (0.56, 0.62)	0.46 (0.44, 0.48)	0.58 (0.55, 0.60)
Region	London	1	1	1	1	1	1
	North East	0.91 (0.81, 1.03)	1.04 (0.92, 1.19)	1.01 (0.90, 1.13)	1.17 (1.05, 1.32)	1.03 (0.93, 1.15)	1.21 (1.08, 1.35)
	North West	1.29 (1.22, 1.36)	1.36 (1.27, 1.44)	1.31 (1.25, 1.38)	1.41 (1.34, 1.49)	1.30 (1.24, 1.36)	1.40 (1.34, 1.48)
	Yorkshire & The Humber	1.51 (1.31, 1.74)	1.54 (1.33, 1.79)	1.48 (1.31, 1.68)	1.55 (1.37, 1.76)	1.44 (1.28, 1.62)	1.53 (1.35, 1.73)
	East Midlands	2.18 (1.69, 2.81)	2.38 (1.84, 3.09)	2.12 (1.70, 2.65)	2.36 (1.88, 2.96)	2.16 (1.75, 2.67)	2.43 (1.96, 3.02)
	West Midlands	1.64 (1.54, 1.75)	1.72 (1.61, 1.84)	1.61 (1.53, 1.70)	1.73 (1.63, 1.83)	1.55 (1.47, 1.63)	1.67 (1.58, 1.76)
	East of England	1.75 (1.63, 1.88)	1.57 (1.46, 1.69)	1.65 (1.55, 1.75)	1.49 (1.40, 1.58)	1.65 (1.56, 1.74)	1.49 (1.41, 1.58)
	South West	1.43 (1.34, 1.52)	1.43 (1.33, 1.52)	1.48 (1.41, 1.56)	1.49 (1.41, 1.57)	1.49 (1.42, 1.57)	1.51 (1.43, 1.59)
	South Central	1.45 (1.37, 1.53)	1.28 (1.21, 1.36)	1.54 (1.47, 1.62)	1.41 (1.34, 1.48)	1.51 (1.44, 1.58)	1.38 (1.32, 1.45)
	South East Coast	1.33 (1.26, 1.41)	1.19 (1.12, 1.26)	1.33 (1.26, 1.39)	1.24 (1.17, 1.30)	1.30 (1.24, 1.36)	1.21 (1.15, 1.27)
Ethnicity	White	1	1	1	1	1	1
	South Asian	0.76 (0.71, 0.81)	0.83 (0.78, 0.88)	0.78 (0.74, 0.83)	0.84 (0.79, 0.88)	0.78 (0.75, 0.82)	0.84 (0.80, 0.89)
	Black	0.45 (0.41, 0.48)	0.61 (0.56, 0.67)	0.46 (0.43, 0.50)	0.61 (0.57, 0.66)	0.47 (0.44, 0.51)	0.63 (0.59, 0.67)
	Mixed	0.65 (0.57, 0.74)	0.72 (0.63, 0.83)	0.64 (0.57, 0.71)	0.70 (0.62, 0.79)	0.63 (0.57, 0.70)	0.69 (0.62, 0.77)
	Other	0.65 (0.59, 0.72)	0.68 (0.62, 0.75)	0.66 (0.61, 0.71)	0.69 (0.63, 0.74)	0.65 (0.61, 0.70)	0.68 (0.63, 0.74)
Maternal age, years	<20	0.79 (0.72, 0.87)	0.81 (0.73, 0.89)	0.73 (0.67, 0.79)	0.73 (0.68, 0.79)	0.74 (0.68, 0.79)	0.74 (0.69, 0.80)
	20-24	1	1	1	1	1	1
	25-29	1.27 (1.21, 1.34)	1.29 (1.22, 1.36)	1.28 (1.23, 1.34)	1.30 (1.25, 1.37)	1.26 (1.21, 1.32)	1.28 (1.23, 1.34)
	30-35	1.54 (1.46, 1.62)	1.55 (1.47, 1.64)	1.55 (1.49, 1.62)	1.57 (1.50, 1.65)	1.54 (1.47, 1.60)	1.55 (1.48, 1.62)
	≥35	1.36 (1.29, 1.44)	1.42 (1.34, 1.51)	1.41 (1.35, 1.48)	1.48 (1.41, 1.55)	1.38 (1.32, 1.44)	1.44 (1.37, 1.51)
Number of children	0	1	1	1	1	1	1
	1	0.67 (0.65, 0.70)	0.65 (0.63, 0.68)	0.69 (0.66, 0.71)	0.67 (0.65, 0.69)	0.70 (0.67, 0.72)	0.68 (0.66, 0.70)
	2	0.47 (0.45, 0.49)	0.47 (0.45, 0.50)	0.50 (0.48, 0.52)	0.49 (0.47, 0.51)	0.50 (0.48, 0.52)	0.50 (0.48, 0.52)
	≥3	0.35 (0.33, 0.37)	0.37 (0.35, 0.40)	0.38 (0.36, 0.40)	0.39 (0.37, 0.42)	0.39 (0.37, 0.41)	0.40 (0.38, 0.42)
Body mass index (BMI)	<18.5	0.71 (0.64, 0.77)		0.69 (0.64, 0.75)		0.71 (0.66, 0.77)	
	18.5-24.9	1		1		1	
	25.0-29.9	1.01 (0.96, 1.05)		0.97 (0.94, 1.01)		0.98 (0.94, 1.01)	
	≥30	0.82 (0.78, 0.86)		0.82 (0.79, 0.85)		0.82 (0.79, 0.85)	

Note: All models include women who registered in first trimester and exclude those with missing ethnicity; minimally adjusted models of BMI excludes women with missing BMI

Abbreviations: UK, United Kingdom; IMD, Index of Multiple Deprivation; BMI, body mass index

For peer review only - http://bmjopen.bmj.com/site/about/guidelines.xhtml

Supplementary Table 6: Sensitivity analyses expanding definition of inclusion criteria for the influenza vaccine uptake models: registration by end of pregnancy, including pregnancies without known outcomes, extending influenza season to March, compared to primary analyses

		Primary analyses		Registered by end of pregnancy		Including pregnancies without known outcomes		Extending influenza season through March	
		Minimally adjusted	Fully adjusted	Minimally adjusted	Fully adjusted	Minimally adjusted	Fully adjusted	Minimally adjusted	Fully adjusted
N		140,141	140,141	153,782	153,782	191,950	191,950	140,141	140,141
Season	2010	1	1	1	1	1	1	1	1
	2011	0.89 (0.86, 0.92)	0.89 (0.86, 0.92)	0.89 (0.86, 0.92)	0.89 (0.86, 0.92)	0.92 (0.89, 0.95)	0.92 (0.89, 0.94)	0.93 (0.90, 0.96)	0.93 (0.90, 0.96)
	2012	1.63 (1.58, 1.69)	1.65 (1.60, 1.71)	1.62 (1.57, 1.67)	1.63 (1.58, 1.68)	1.55 (1.51, 1.60)	1.56 (1.52, 1.61)	1.81 (1.76, 1.87)	1.84 (1.78, 1.90)
	2013	1.40 (1.35, 1.45)	1.40 (1.35, 1.45)	1.41 (1.36, 1.45)	1.41 (1.36, 1.46)	1.36 (1.32, 1.41)	1.36 (1.32, 1.41)	1.57 (1.51, 1.62)	1.57 (1.51, 1.62)
	2014	1.69 (1.63, 1.76)	1.70 (1.63, 1.76)	1.71 (1.65, 1.77)	1.72 (1.65, 1.78)	1.64 (1.59, 1.70)	1.63 (1.58, 1.69)	1.88 (1.81, 1.95)	1.89 (1.82, 1.96)
	2015	1.65 (1.58, 1.73)	1.68 (1.60, 1.76)	1.67 (1.60, 1.75)	1.70 (1.63, 1.78)	1.61 (1.54, 1.68)	1.61 (1.55, 1.68)	1.86 (1.78, 1.94)	1.89 (1.81, 1.98)
IMD	Least deprived	1	1	1	1	1	1	1	1
	2	0.90 (0.87, 0.93)	0.92 (0.89, 0.95)	0.88 (0.86, 0.91)	0.90 (0.87, 0.94)	0.87 (0.84, 0.90)	0.89 (0.86, 0.92)	0.90 (0.87, 0.93)	0.91 (0.88, 0.95)
	3	0.84 (0.81, 0.86)	0.88 (0.85, 0.91)	0.83 (0.81, 0.86)	0.87 (0.84, 0.90)	0.83 (0.80, 0.85)	0.87 (0.85, 0.90)	0.83 (0.81, 0.86)	0.87 (0.84, 0.90)
	4	0.71 (0.69, 0.74)	0.76 (0.74, 0.79)	0.71 (0.69, 0.73)	0.76 (0.74, 0.79)	0.71 (0.69, 0.74)	0.78 (0.75, 0.80)	0.70 (0.68, 0.73)	0.75 (0.73, 0.78)
	Most deprived	0.67 (0.65, 0.70)	0.72 (0.70, 0.75)	0.67 (0.65, 0.69)	0.72 (0.69, 0.74)	0.69 (0.67, 0.71)	0.76 (0.73, 0.78)	0.67 (0.65, 0.69)	0.71 (0.69, 0.74)
Region	London	1	1	1	1	1	1	1	1
	North East	1.11 (1.02, 1.21)	1.21 (1.11, 1.31)	1.14 (1.06, 1.24)	1.24 (1.14, 1.34)	1.15 (1.07, 1.24)	1.25 (1.16, 1.35)	1.09 (1.01, 1.18)	1.19 (1.09, 1.29)
	North West	1.37 (1.32, 1.42)	1.42 (1.36, 1.47)	1.39 (1.34, 1.45)	1.44 (1.39, 1.50)	1.42 (1.38, 1.47)	1.48 (1.42, 1.53)	1.38 (1.33, 1.44)	1.44 (1.38, 1.50)
	Yorkshire & The Humber	1.27 (1.18, 1.38)	1.26 (1.16, 1.37)	1.32 (1.22, 1.43)	1.31 (1.21, 1.42)	1.33 (1.24, 1.43)	1.33 (1.23, 1.43)	1.27 (1.17, 1.38)	1.26 (1.17, 1.37)
	East Midlands	1.33 (1.21, 1.47)	1.40 (1.27, 1.55)	1.34 (1.22, 1.47)	1.41 (1.29, 1.55)	1.33 (1.23, 1.45)	1.39 (1.28, 1.52)	1.35 (1.23, 1.49)	1.43 (1.30, 1.58)
	West Midlands	1.41 (1.35, 1.46)	1.43 (1.37, 1.49)	1.42 (1.37, 1.48)	1.45 (1.39, 1.51)	1.43 (1.38, 1.48)	1.45 (1.40, 1.51)	1.47 (1.41, 1.53)	1.50 (1.44, 1.57)
	East of England	1.31 (1.26, 1.37)	1.24 (1.19, 1.30)	1.31 (1.26, 1.37)	1.24 (1.19, 1.30)	1.31 (1.26, 1.36)	1.24 (1.19, 1.29)	1.32 (1.26, 1.37)	1.25 (1.20, 1.31)
	South West	1.25 (1.20, 1.31)	1.25 (1.20, 1.31)	1.29 (1.24, 1.35)	1.29 (1.24, 1.35)	1.34 (1.29, 1.39)	1.34 (1.29, 1.39)	1.27 (1.22, 1.32)	1.27 (1.22, 1.33)
	South Central	1.42 (1.36, 1.47)	1.33 (1.28, 1.38)	1.45 (1.40, 1.50)	1.36 (1.31, 1.41)	1.47 (1.42, 1.52)	1.38 (1.33, 1.43)	1.43 (1.38, 1.48)	1.34 (1.29, 1.40)
	South East Coast	1.06 (1.02, 1.10)	1.02 (0.98, 1.06)	1.07 (1.04, 1.11)	1.03 (0.99, 1.07)	1.11 (1.07, 1.15)	1.07 (1.03, 1.11)	1.05 (1.01, 1.09)	1.01 (0.97, 1.05)
Ethnicity	White	1	1	1	1	1	1	1	1
	South Asian	0.92 (0.88, 0.95)	0.99 (0.95, 1.03)	0.92 (0.88, 0.95)	0.99 (0.95, 1.03)	0.93 (0.90, 0.96)	0.98 (0.95, 1.02)	0.94 (0.91, 0.98)	1.02 (0.98, 1.06)
	Black	0.67 (0.64, 0.71)	0.83 (0.78, 0.88)	0.68 (0.64, 0.71)	0.83 (0.78, 0.88)	0.69 (0.65, 0.72)	0.83 (0.79, 0.87)	0.67 (0.64, 0.71)	0.83 (0.79, 0.88)
	Mixed	0.84 (0.77, 0.92)	0.91 (0.83, 0.99)	0.78 (0.72, 0.85)	0.84 (0.77, 0.92)	0.79 (0.73, 0.86)	0.86 (0.79, 0.93)	0.83 (0.76, 0.91)	0.90 (0.82, 0.99)
	Other	0.79 (0.73, 0.84)	0.85 (0.79, 0.91)	0.75 (0.70, 0.80)	0.81 (0.76, 0.86)	0.77 (0.73, 0.82)	0.83 (0.78, 0.88)	0.80 (0.75, 0.85)	0.86 (0.80, 0.92)
Maternal age, years	<20	0.87 (0.81, 0.92)	0.87 (0.82, 0.93)	0.87 (0.82, 0.93)	0.87 (0.82, 0.93)	0.68 (0.65, 0.72)	0.68 (0.64, 0.71)	0.88 (0.82, 0.93)	0.88 (0.83, 0.94)
	20-24	1	1	1	1	1	1	1	1
	25-29	1.13 (1.09, 1.17)	1.12 (1.08, 1.16)	1.14 (1.10, 1.18)	1.13 (1.10, 1.17)	1.24 (1.20, 1.28)	1.25 (1.21, 1.29)	1.13 (1.09, 1.17)	1.13 (1.09, 1.17)
	30-35	1.22 (1.18, 1.26)	1.21 (1.17, 1.25)	1.24 (1.20, 1.28)	1.23 (1.19, 1.27)	1.36 (1.32, 1.41)	1.38 (1.34, 1.42)	1.23 (1.19, 1.27)	1.22 (1.17, 1.26)
	≥35	1.17 (1.12, 1.21)	1.18 (1.13, 1.22)	1.19 (1.15, 1.23)	1.20 (1.15, 1.24)	1.18 (1.14, 1.21)	1.21 (1.17, 1.25)	1.16 (1.12, 1.21)	1.18 (1.14, 1.23)
	0	1	1	1	1	1	1	1	1

For peer review only - http://bmjopen.bmj.com/site/about/guidelines.xhtml

Number of children	1	0.80 (0.78, 0.82)	0.80 (0.78, 0.82)	0.84 (0.82, 0.86)	0.83 (0.81, 0.85)	0.87 (0.85, 0.89)	0.86 (0.84, 0.88)	0.79 (0.77, 0.80)	0.78 (0.76, 0.80)
	2	0.71 (0.68, 0.73)	0.71 (0.69, 0.74)	0.75 (0.72, 0.77)	0.74 (0.72, 0.77)	0.72 (0.70, 0.75)	0.71 (0.68, 0.73)	0.69 (0.67, 0.71)	0.69 (0.67, 0.72)
	≥3	0.61 (0.58, 0.63)	0.62 (0.59, 0.65)	0.64 (0.61, 0.67)	0.65 (0.62, 0.68)	0.63 (0.61, 0.66)	0.63 (0.60, 0.66)	0.59 (0.56, 0.61)	0.60 (0.57, 0.63)
Clinical risk group	No	1	1	1	1	1	1	1	1
	Yes	1.69 (1.62, 1.76)	1.70 (1.63, 1.77)	1.73 (1.66, 1.80)	1.73 (1.66, 1.80)	1.98 (1.91, 2.06)	2.00 (1.93, 2.07)	1.59 (1.53, 1.66)	1.60 (1.54, 1.67)
BMI	<18.5	0.85 (0.80, 0.90)		0.84 (0.79, 0.89)		0.84 (0.79, 0.88)		0.93 (0.88, 0.98)	
	18.5-24.9	1		1		1		1	
	25.0-29.9	1.04 (1.01, 1.07)		1.03 (1.01, 1.06)		1.04 (1.02, 1.07)		0.98 (0.95, 1.00)	
	≥30	1.00 (0.97, 1.03)		0.99 (0.96, 1.02)		1.03 (1.00, 1.06)		0.90 (0.87, 0.92)	

Note: All models include women who registered in first trimester, and exclude those with outcome unknown and missing ethnicity; minimally adjusted model of BMI excludes women with missing BMI

Abbreviations: UK, United Kingdom; IMD, Index of Multiple Deprivation; BMI, body mass index

For peer review only

Supplementary Table 7: Secondary analysis of subsequent pertussis vaccine uptake among women who had received pertussis vaccination in their first eligible pregnancy and had a second eligible pregnancy within the study period (N=3,111)

		**Total (column %)**	**Received pertussis vaccine in second pregnancy (row %)**	**Minimally adjusted model** OR of receiving vaccine in second pregnancy (95% CI)	**Fully adjusted model** OR of receiving vaccine in second pregnancy (95% CI)
N		3,111	1,877 (60.3)		
Year of first pregnancy	2012	550 (17.7)	380 (69.1)	1	1
	2013	1,912 (61.5)	1,264 (66.1)	0.87 (0.71-1.07)	0.70 (0.56-0.87)
	2014-15	649 (20.9)	233 (35.9)	0.25 (0.20-0.32)	0.14 (0.10-0.18)
Index of multiple deprivation (IMD) quintile	Least deprived	857 (27.6)	539 (62.9)	1	1
	2	539 (17.3)	326 (60.5)	0.90 (0.71-1.13)	0.91 (0.71-1.16)
	3	604 (19.4)	381 (63.1)	1.03 (0.92-1.28)	1.06 (0.83-1.35)
	4	579 (18.6)	337 (58.2)	0.82(0.66-1.02)	0.89 (0.70-1.15)
	Most deprived	532 (17.1)	294 (55.3)	0.72 (0.57-0.90)	0.77 (0.59-1.01)
Region	London	453 (14.6)	260 (57.4)	1	1
	North East	35 (1.1)	22 (62.9)	1.25 (0.65-2.83)	2.08 (0.95-4.58)
	North West	390 (12.5)	240 (61.5)	1.16 (0.87-1.55)	1.29 (0.95-1.77)
	Yorkshire & The Humber	31 (1.0)	14 (45.2)	0.56 (0.27-1.19)	0.73 (0.33-1.62)
	East Midlands	0	0	-	-
	West Midlands	375 (12.1)	229 (61.1)	1.13 (0.85-1.51)	1.33 (0.97-1.81)
	East of England	296 (9.5)	201 (67.9)	1.57 (1.14-2.15)	1.54 (1.10-2.16)
	South West	388 (12.5)	239 (61.6)	1.19 (0.98-1.58)	1.31 (0.96-1.79)
	South Central	562 (18.1)	360 (64.1)	1.33 (1.02-1.73)	1.31 (0.99-1.74)
	South East Coast	581 (18.7)	312 (53.7)	0.90 (0.69-1.16)	0.99 (0.75-1.31)
Ethnicity	White	2,732 (87.8)	1,657 (60.7)	1	1
	South Asian	204 (6.6)	114 (55.9)	0.82 (0.61-1.10)	0.78 (0.57-1.07)
	Black	84 (2.7)	49 (58.3)	1.05 (0.66-1.67)	1.09 (0.66-1.80)
	Mixed	33 (1.1)	20 (60.6)	0.94 (0.46-1.94)	1.14 (0.63-2.07)
	Other	58 (1.9)	37 (63.8)	1.25 (0.71-2.20)	0.97 (0.46-2.06)
Maternal age, years	<20	102 (3.2)	40 (39.2)	0.48 (0.30-0.75)	0.48 (0.30-0.77)
	20-24	505 (16.2)	290 (57.4)	1	1
	25-29	1,002 (32.2)	592 (59.1)	1.07 (0.85-1.34)	1.09 (0.86-1.39)
	30-34	1,048 (33.7)	669 (63.8)	1.32 (1.05-1.65)	1.26 (0.98-1.61)
	≥35	454 (14.6)	286 (63.0)	1.29 (0.99-1.69)	1.26 (0.94-1.69)
Number of children	0	1,936 (62.2)	1,224 (63.2)	1	1
	1	714 (23.0)	405 (56.7)	0.78 (0.65-0.94)	0.75 (0.62-0.91)
	2	264 (8.5)	149 (56.4)	0.72 (0.55-0.95)	0.64 (0.48-0.85)
	≥3	197 (6.3)	99 (50.3)	0.56 (0.42-0.76)	0.50 (0.36-0.69)
Pregnancy interval (days from end of first pregnancy to start of second)	0-179	416 (13.4)	227 (54.6)	1	1
	180-359	749 (24.1)	476 (63.6)	1.25 (0.96-1.63)	1.11 (0.85-1.45)
	360-539	1,004 (32.3)	695 (69.2)	1.33 (1.03-1.71)	1.13 (0.86-1.47)
	540-719	624 (20.1)	373 (59.8)	0.65 (0.49-0.85)	0.54 (0.41-0.73)
	720+	318 (10.2)	106 (33.3)	0.19 (0.14-0.27)	0.16 (0.11-0.22)

Note: Among 3,363 women with two eligible pregnancies during follow up, excluded 2 with implausible (<0 days) spacing between the end of the first pregnancy and start of the second, and 250 with missing ethnicity data.

For peer review only - http://bmjopen.bmj.com/site/about/guidelines.xhtml

The RECORD statement – checklist of items, extended from the STROBE statement, that should be reported in observational studies using routinely collected health data.

	Item No.	STROBE items	Location in manuscript where items are reported	RECORD items	Location in manuscript where items are reported
**Title and abstract**					
	1	(a) Indicate the study's design with a commonly used term in the title or the abstract (b) Provide in the abstract an informative and balanced summary of what was done and what was found	Title and abstract	RECORD 1.1: The type of data used should be specified in the title or abstract. When possible, the name of the databases used should be included.<br><br>RECORD 1.2: If applicable, the geographic region and timeframe within which the study took place should be reported in the title or abstract.<br><br>RECORD 1.3: If linkage between databases was conducted for the study, this should be clearly stated in the title or abstract.	Title and abstract<br><br>Abstract<br><br>No new linkage conducted for the study (use of pre-linked data described in methods)
**Introduction**					
Background rationale	2	Explain the scientific background and rationale for the investigation being reported	Introduction pages 5-6		
Objectives	3	State specific objectives, including any prespecified hypotheses	Introduction page 6		
**Methods**					
Study Design	4	Present key elements of study design early in the paper	Abstract and methods page 7		
Setting	5	Describe the setting, locations, and relevant dates, including	Abstract and methods page 7		

		periods of recruitment, exposure, follow-up, and data collection			
Participants	6	*(a) Cohort study* - Give the eligibility criteria, and the sources and methods of selection of participants. Describe methods of follow-up<br>*Case-control study* - Give the eligibility criteria, and the sources and methods of case ascertainment and control selection. Give the rationale for the choice of cases and controls<br>*Cross-sectional study* - Give the eligibility criteria, and the sources and methods of selection of participants<br><br>*(b) Cohort study* - For matched studies, give matching criteria and number of exposed and unexposed<br>*Case-control study* - For matched studies, give matching criteria and the number of controls per case	Cohort – methods pages 7-8<br><br>N/A Cohort – no matching	RECORD 6.1: The methods of study population selection (such as codes or algorithms used to identify subjects) should be listed in detail. If this is not possible, an explanation should be provided.<br><br>RECORD 6.2: Any validation studies of the codes or algorithms used to select the population should be referenced. If validation was conducted for this study and not published elsewhere, detailed methods and results should be provided.<br><br>RECORD 6.3: If the study involved linkage of databases, consider use of a flow diagram or other graphical display to demonstrate the data linkage process, including the number of individuals with linked data at each stage.	Cohort – methods pages 7-8<br><br>N/A<br><br>No new data linkages
Variables	7	Clearly define all outcomes, exposures, predictors, potential confounders, and effect modifiers. Give diagnostic criteria, if applicable.	Methods pages 9-10	RECORD 7.1: A complete list of codes and algorithms used to classify exposures, outcomes, confounders, and effect modifiers should be provided. If these cannot be reported, an explanation should be provided.	Methods pages 9-10
Data sources/ measurement	8	For each variable of interest, give sources of data and details of methods of assessment (measurement).	Methods pages 9-10		

For peer review only

		Describe comparability of assessment methods if there is more than one group			
Bias	9	Describe any efforts to address potential sources of bias	Methods page 11-12		
Study size	10	Explain how the study size was arrived at	Methods page 6-8		
Quantitative variables	11	Explain how quantitative variables were handled in the analyses. If applicable, describe which groupings were chosen, and why	Methods page 10		
Statistical methods	12	(a) Describe all statistical methods, including those used to control for confounding<br>(b) Describe any methods used to examine subgroups and interactions<br>(c) Explain how missing data were addressed<br>(d) *Cohort study* - If applicable, explain how loss to follow-up was addressed<br>*Case-control study* - If applicable, explain how matching of cases and controls was addressed<br>*Cross-sectional study* - If applicable, describe analytical methods taking account of sampling strategy<br>(e) Describe any sensitivity analyses	Methods page 10-12<br>N/A<br>Methods page 12<br>Methods page 9<br>Methods page 12		
Data access and cleaning methods		..		RECORD 12.1: Authors should describe the extent to which the investigators had access to the database population used to create the study population.	Page 22 author contributions

For peer review only

				RECORD 12.2: Authors should provide information on the data cleaning methods used in the study.	Methods pages 9-10, and results page 14
Linkage		..		RECORD 12.3: State whether the study included person-level, institutional-level, or other data linkage across two or more databases. The methods of linkage and methods of linkage quality evaluation should be provided.	No data linkage – this study used pre-linked data only, as described in Methods page 9
**Results**					
Participants	13	(a) Report the numbers of individuals at each stage of the study (*e.g.*, numbers potentially eligible, examined for eligibility, confirmed eligible, included in the study, completing follow-up, and analysed)<br>(b) Give reasons for non-participation at each stage.<br>(c) Consider use of a flow diagram	Methods page 9, results page 14	RECORD 13.1: Describe in detail the selection of the persons included in the study (*i.e.,* study population selection) including filtering based on data quality, data availability and linkage. The selection of included persons can be described in the text and/or by means of the study flow diagram.	Methods page 9, results page 14
Descriptive data	14	(a) Give characteristics of study participants (*e.g.*, demographic, clinical, social) and information on exposures and potential confounders<br>(b) Indicate the number of participants with missing data for each variable of interest<br>(c) *Cohort study* - summarise follow-up time (*e.g.*, average and total amount)	Results page 14, Tables 1 and 2<br>Results page 14 and supplementary table 2<br>N/A		
Outcome data	15	*Cohort study* - Report numbers of outcome events or summary measures over time	Tables 1 and 2		

For peer review only

		Case-control study - Report numbers in each exposure category, or summary measures of exposure<br>*Cross-sectional study* - Report numbers of outcome events or summary measures			
Main results	16	(a) Give unadjusted estimates and, if applicable, confounder-adjusted estimates and their precision (e.g., 95% confidence interval). Make clear which confounders were adjusted for and why they were included<br>(b) Report category boundaries when continuous variables were categorized<br>(c) If relevant, consider translating estimates of relative risk into absolute risk for a meaningful time period	Tables 1 and 2<br><br>Tables 1 and 2<br><br>N/A		
Other analyses	17	Report other analyses done—e.g., analyses of subgroups and interactions, and sensitivity analyses	Methods page 12-13, supplementary tables 3-7		
**Discussion**					
Key results	18	Summarise key results with reference to study objectives	Discussion page 19		
Limitations	19	Discuss limitations of the study, taking into account sources of potential bias or imprecision. Discuss both direction and magnitude of any potential bias	Discussion page 19	RECORD 19.1: Discuss the implications of using data that were not created or collected to answer the specific research question(s). Include discussion of misclassification bias, unmeasured confounding, missing data, and changing eligibility over time, as they pertain to the study being reported.	Discussion page 19

For peer review only

Interpretation	20	Give a cautious overall interpretation of results considering objectives, limitations, multiplicity of analyses, results from similar studies, and other relevant evidence	Discussion pages 20-21		
Generalisability	21	Discuss the generalisability (external validity) of the study results	Discussion page 19 re other settings		
**Other Information**					
Funding	22	Give the source of funding and the role of the funders for the present study and, if applicable, for the original study on which the present article is based	Funding statement		
Accessibility of protocol, raw data, and programming code		..		RECORD 22.1: Authors should provide information on how to access any supplemental information such as the study protocol, raw data, or programming code.	Methods page 10

*Reference: Benchimol EI, Smeeth L, Guttmann A, Harron K, Moher D, Petersen I, Sørensen HT, von Elm E, Langan SM, the RECORD Working Committee. The REporting of studies Conducted using Observational Routinely-collected health Data (RECORD) Statement. *PLoS Medicine* 2015; in press.

*Checklist is protected under Creative Commons Attribution (CC BY) license.

For peer review only
